# Expanding the Structural Diversity of N–H Borazines: A Hf(OTf)_4_‑Catalyzed Synthesis from Aryl Boronates under Microwave Irradiation

**DOI:** 10.1021/acs.orglett.6c01514

**Published:** 2026-05-11

**Authors:** Ejdi Cela, Alireza Nazari Khodadadi, Fan Huang, Dario Marchionni, Luigi Vaccaro

**Affiliations:** Laboratory of Green S.O.C. - Dipartimento di Chimica, Biologia e Biotecnologie, 9309 Università degli Studi di Perugia, Via Elce di Sotto 8, 06123 Perugia, Italy

## Abstract

For decades, the synthesis of *B*,*B*′,*B*″-tri­(aryl)­borazines (N–H borazines) has been restricted to a limited number of studies with minimal structural diversity explored. Addressing this limitation, we developed a synthetic method exploiting cyclic boronates and a commercially available hafnium complex, Hf­(OTf)_4_, as a catalyst. A range of N–H borazines is formed under microwave irradiation in a biomass-derived solvent, in a short reaction time of just 45 min, with high yields. This approach improved synthetic efficiency and functional group compatibility, enabling a more sustainable process and paving the way for further exploration of borazine applications.

## Graphical abstract

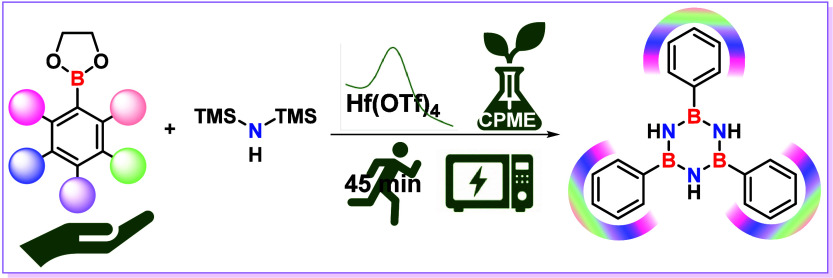

Often called “inorganic benzene”, borazine’s properties are defined by the inherent polarity of the B–N bond. Facile access to these cyclic B–N motifs is essential for the structural diversification of (two-dimensional) hybrid boron–nitrogen–carbon (BNC) materials. Compared to benzene, the borazine core exhibits reduced aromaticity^1^ and a larger bandgap^2^ that can be tuned by substitution patterns.^3^ Consequently, borazine is a key component in the development of BNC (molecular) materials for optoelectronic applications,^4,5^ gas storage,^6^ catalysis, and ceramic materials.^7,8^

Despite the interesting properties of borazine compared with those of benzene and boroxine, the library and application of organoborazines are very limited. This limitation stems from the lack of effective synthetic methodologies for accessing various functionalized borazine cores. In the case of hexaaryl-substituted borazines (HABs), significant progress has been made regarding sustainability, scalability, and the handling of air-sensitive and reactive intermediates, including chloroborazoles and organolithium species.^9,10^ Specifically, a continuous flow process has been developed by our group for the synthesis of HABs,^11^ while mechanochemistry has emerged as a powerful tool for the late-stage functionalization of the outer sphere.^12,13^

However, the situation remains significantly different for borazines featuring a substitution at the boron atom and a “free” N–H functionality (N–H borazines).

These N–H borazine molecular entities are of significant interest due to their chemical and physical properties and the potential for further modifications through the N-site. For instance, Zhu et al. demonstrated the application of *B*,*B*′,*B*″-tri­(aryl)­borazines as hole transporting materials (HTMs) in organic light-emitting diodes (OLEDs).^14^ Furthermore, borazine-incorporated arylacetylene resins (PBZA) have been utilized as precursors for a variety of advanced materials, including cross-linked polymers with low dielectric constant,^15^ h-BNC and nanocrystalline graphite,^16^ borazine-doped graphene nanosheets,^17^ and activated carbon material loaded with B_3_N_3_-doped carbon nanosheets (17 wt %) used as a catalyst for dehydrochlorination reactions.^18^

While offering significant potential, the efficient synthesis of this type of borazines remains a significant challenge. Current methodologies are hampered by unsatisfactory yields, rarely exceeding 40%,^19–21^ or prohibitively long reaction times of up to 10 days.^22^ Moreover, existing reports are often limited to a single compound or an extremely narrow substrate scope. This lack of accessible structural diversity has largely relegated these molecules to a niche concern, preventing the systematic exploration of their properties and applications.

To address these limitations and facilitate diverse substitution patterns, we developed a methodology using stable aryl boronic acid esters, *R*-B­(OR)_2_ (boronates), as safer alternatives to the highly reactive and air-sensitive RBCl_2_ (Figure 1). By employing an oxophilic transition-metal catalyst to compensate for the lower reactivity of boronates in borazine ring formation, we established a streamlined route to a broad library of functionalized derivatives.

**Figure 1:**
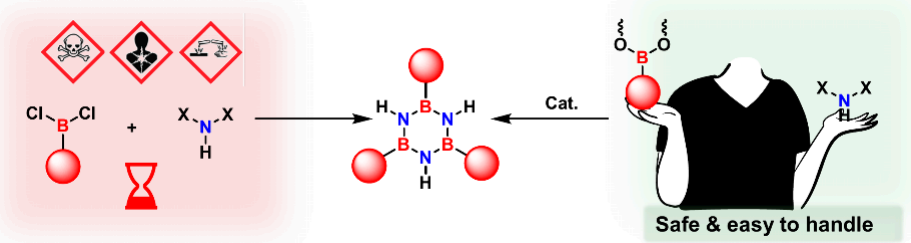
Selection of safer starting materials for the synthesis of the borazine core.

Our investigation started with representative substrates 2-[4-(trifluoromethyl)­phenyl]-1,3,2-dioxaborolane (**1a**) and hexamethyldisilazane (HMDS, **2**) as the boron and nitrogen sources, respectively. Substrate **1a** was specifically selected because the electron-withdrawing CF_3_ group in the *para*-position is anticipated to reduce electron density at the boron center, thereby enhancing its Lewis acidity.^23^

Following reagent selection, efforts focused on developing the synthetic methodology. Initial attempts using solvothermal conditions without a catalyst provided unsatisfactory results (<5% isolated yield), as detailed in Table S1. This outcome was anticipated, given the inherent acid/base properties of the boronate **1a** and the aminosilane precursors. To compensate for the lower Lewis acidity of boronates compared with that of boron halides, a Lewis acid catalyst was considered essential to promote the reaction and achieve high yields within short reaction times.

We initiated a catalyst screening using transition metals (TMs) with high oxygen affinity, a strategy designed to weaken the B–O bond and enhance the Lewis acidity of the boron center. Initial screening under conventional oil bath heating provided the desired product **3a** in moderate yields. Beyond the oxophilicity of the metal center, the ligand environment proved crucial for catalytic efficiency. This is exemplified by comparing entries 2 and 3 in Table 1. Specifically, substituting the chloride (Cl^–^) with the more electron-deficient and weakly coordinating trifluoromethanesulfonate (OTf^–^) group enhances the Lewis acidity of the hafnium center, increasing the isolated yield from 20% to 56%, respectively. Given that metal triflates like Hf­(OTf)_4_ have been used for desilylation,^24^ we propose that the catalyst plays a dual role in the desilylation of HMDS.

**Table 1:**
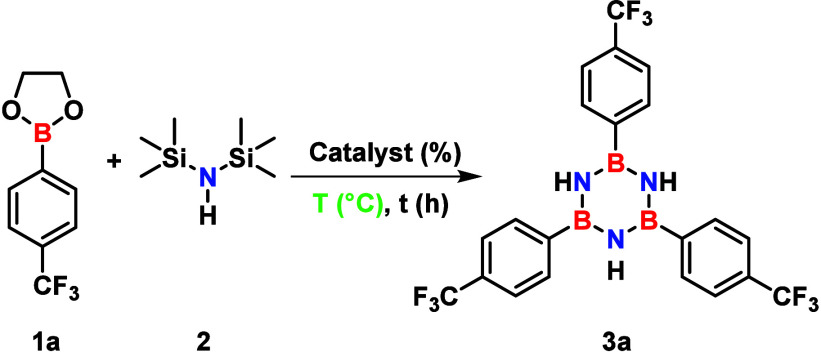
Evaluation of Oxophilic Lewis Acid Catalysts and Heating Method for the Synthesis of Borazine **3a** ^a^

#	Catalyst	Heating	Temp. (°C)	Time (h)	Yield (%)^b^
1^c^	TiCl_4_ (30%)	Oil bath	130	72	15
2	HfCl_4_ (30%)	Oil bath	130	72	20
3	Hf(OTf)_4_(30%)	Oil bath	130	72	56
4	Hf(OTf)_4_ (15%)	Oil bath	130	72	51
5	Hf(OTf)_4_ (15%)	Oil bath	130	7	17
6	Hf(OTf)_4_ (15%)	MW	150	7	75
7	Hf(OTf)_4_ (30%)	MW	150	5	83

a Reaction conditions: boronate (**1a**, 1 mmol); HMDS (**2**, 14.32 equiv, 3 mL).

b Isolated yields.

c TiCl_4_ was in DCM solution.

To address the prohibitive reaction times of 49–72 h, we steered the optimization to the use of microwave irradiation. While in microwave-assisted organic synthesis (MAOS) the specific influence of nonthermal microwave effects remains a subject of ongoing debate,^25^ the advantages of dielectric heating in accelerating reaction rates and improving outcomes are well documented.^26^ Consequently, Hf­(OTf)_4_ was selected as the optimal catalyst to investigate the influence of the heating mode on our target process. As summarized in Table 1 and Table S2, the results underscore the significant advantages of microwave irradiation, which drastically reduced reaction times while enhancing yields.

Under identical catalyst loading (15 mol %), microwave irradiation of 7 h significantly improved the yield to 75%, compared to 17% achieved under conventional heating (Table 1, entries 5 and 6). Doubling the catalyst amount to 30 mol % achieved 83% yield in 5 h, though shortening the time to 3 h (Table 2, entry 1) resulted in a substantial decrease to 32%. We therefore sought to further optimize the protocol to minimize both the time and the catalyst loading.

While oxophilicity (Θ) is frequently correlated with absolute hardness,^27^ Kepp^28^ identified electronegativity and effective nuclear charge as the primary determining factors, where lower values favor increased oxophilicity. Guided by these principles, we evaluated the use of well-known oxophilic TM catalysts. However, none outperformed Hf­(OTf)_4_, which possesses the highest computed Θ value among the metals tested (Table 2 and Table S3). Furthermore, decreasing the volume of HMDS (**2**) negatively impacted the reaction outcome, as observed with Sc­(OTf)_3_ (Table 2, entries 2 and 3). This sensitivity likely stems from reaction heterogeneity; higher volumes of **2** (14.32 equiv, 3 mL vs 4.80 equiv, 1 mL) facilitate better dispersion of boronate **1a** and the catalyst, whereas lower volumes result in poor mass transfer.

**Table 2:**
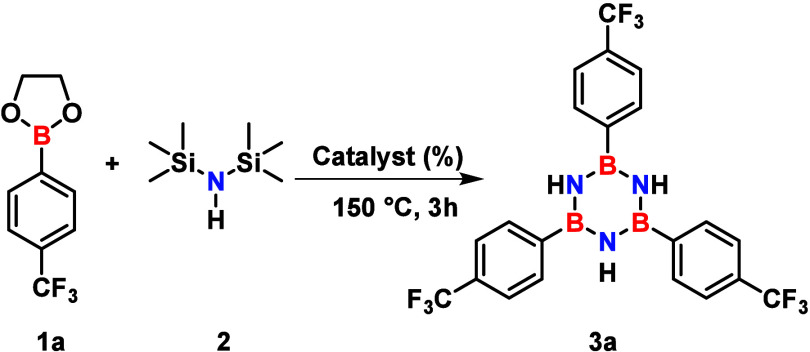
Screening of Oxophilic TM Catalysts in MW Conditions^a^

#	Catalyst (30 mol %)	Oxophilicity (Θ)	HMDS (equiv)	Yield (%)^b^
1	Hf(OTf)_4_	1.0	14.32	32
2	Sc(OTf)_3_	0.8	14.32	20
3	Sc(OTf)_3_	0.8	4.80	4
4	ZrCl_4_	0.8	14.32	15
5	Mg(OTf)_2_	0.6	14.32	7
6	AgOTf	0.2	14.32	6

a Reaction conditions: boronate (**1a**, 1 mmol), 150 °C, 3 h, MW.

b Isolated yields.

Having established microwave irradiation and Hf­(OTf)_4_ as the optimal reaction parameters, we identified the reagent solubility as the primary bottleneck for further optimization. The optimization reactions performed to this point were all under neat conditions, requiring an excess of HMDS to maintain homogeneity. However, its inefficient recovery raises sustainability concerns. Therefore, to overcome the solubility problems, we evaluated several green,^29^ microwave-compatible solvents to enhance the solubility of boronate **1a** while simultaneously reducing the required equivalents of HMDS (**2**) and catalyst. Interestingly, evaluation of catalyst loading revealed that 10 mol % of Hf­(OTf)_4_ consistently outperformed 30 mol % loading across the media tested (Table 3).

**Table 3:**
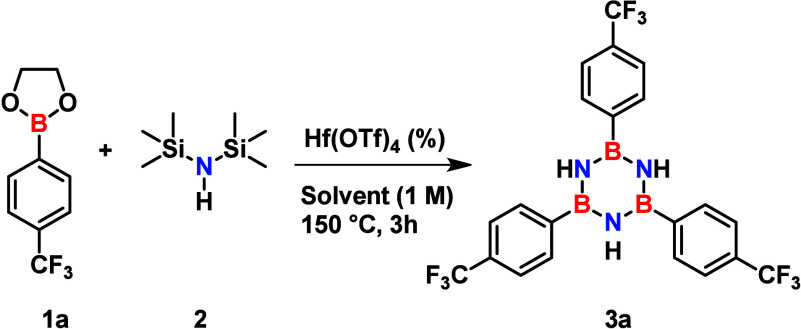
Screening of Reaction Media and Catalyst Loading for the Synthesis of Borazine **3a** ^a^

#	Hf(OTf)_4_ (mol %)	Solvent	HMDS (equiv)	Yield (%)^b^
1	10	Neat	4.80	Trace
2^c^	10	GVL	4.80	0
3	10	2-MeTHF	4.80	68
4^c^	10	PolarClean	4.80	0
5	10	CPME	4.80	87
6	10	4-MeTHP	4.80	83
7	30	Neat	4.80	72
8	30	GVL	4.80	0
9	30	2-MeTHF	4.80	0
10	30	PolarClean	4.80	0
11	30	CPME	4.80	45
12	30	4-MeTHP	4.80	65

a Reaction conditions: boronate (**1a**, 1 mmol), HMDS (**2**, 4.80 equiv, 1 mL), anhydrous solvent (1 mL, 1 M), 150 °C, 3 h, MW.

b Isolated yields.

c Product could not be separated.

As shown in Table 3, GVL and PolarClean failed to afford borazine, instead yielding a persistent oily residue. This residue presumably results from linear polymeric species originating either from solvent degradation promoted by the combined presence of the amine (HMDS) and the Lewis acid catalyst,^30^ linear silazanes, or noncyclized carbon–boron–nitrogen oligomers.^21^ In contrast, CPME and 4-MeTHP improved efficiency at both 10 and 30 mol % catalyst loadings. Higher catalyst loading at a fixed amount of solvent diminished the efficiency, possibly due to aggregation-induced deactivation or limited catalyst solubility. Given the goal of minimizing the catalyst usage, CPME was selected for further optimization. Its superior performance is attributed to several key properties, including a high boiling point and excellent compatibility with Lewis acid catalysts such as Hf­(OTf)_4_.^31^ Notably, CPME features a low peroxide formation rate and a significantly low water solubility (0.3 g/100 g),^32^ which is significantly lower compared to 2-methyltetrahydrofuran (2-MeTHF, 4.4 g/100 g). We believe that this feature is of great utility in the process under study as it facilitates the maintenance of anhydrous conditions. Given these favorable characteristics and the high yields achieved with a reduced catalyst loading of 10 mol % of Hf­(OTf)_4_, CPME was selected for the final optimization of reaction time and HMDS (**2**) stoichiometry.

To optimize the reagent stoichiometry, the amount of HMDS (**2**) was initially reduced from 4.8 equiv (1.0 mL) to 1 equivalent (0.210 mL), which led to a significant decrease in efficiency (Table 4, entry 2). Increasing the quantity of **2** to 1.55 equiv (0.325 mL) (Table 4, entry 3) restored the reaction performance, achieving an 85% isolated yield, which is comparable to the 87% obtained using 4.80 equiv of the reagent. Subsequently, a reaction time study conducted under these refined conditions identified 45 min as the optimal duration, delivering the desired borazine **3a** in an almost quantitative isolated yield of 98%.

**Table 4:**
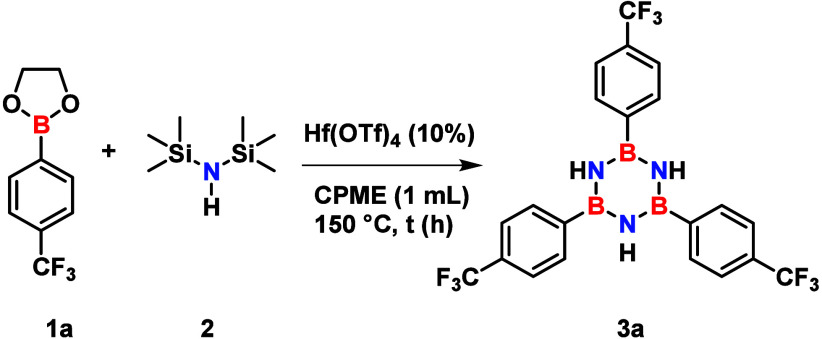
Quantity of HMDS (**2**) and Reaction Time Studies^a^

#	HMDS (**2**, equiv)	Time (h)	Yield (%)^b^
1	4.80	3	87
2	1	3	44
3	1.55	3	85
4	1.55	2	86
5	1.55	1	89
6	1.55	45 min	98
7	1.55	30 min	86
8	1.55	15 min	77

a Reaction conditions: boronate (**1a**, 1 mmol), anhydrous CPME (1 mL, 1 M), Hf­(OTf)_4_ (10 mol %), 150 °C.

b Isolated yields.

We also wish to highlight that we investigated the use of pinacol boronates instead of ethylene glycol boronates. However, we did not obtain satisfactory results, which we attribute to the increased steric hindrance of the pinacol moiety and the stronger O→B π-donation, both of which may hamper/reduce the Lewis acidity of the boron center.

With the optimized reaction conditions in hand, we investigated the influence of the boronate precursor’s electronic and steric properties on the hafnium-catalyzed cyclocondensation. All the results are reported in Scheme 1. Although the diminished Lewis acidity of boronates relative to traditional boron halides was mitigated by the oxophilic catalyst, we anticipated that electronic effects and steric hindrance would significantly impact the reaction outcome. However, our results indicate that the procedure is largely independent of the electronic properties of the substituents. Specifically, the expected correlation between electron-withdrawing groups and increased yields, driven by enhanced Lewis acidity at the boron center, was not observed.

**Scheme 1:**
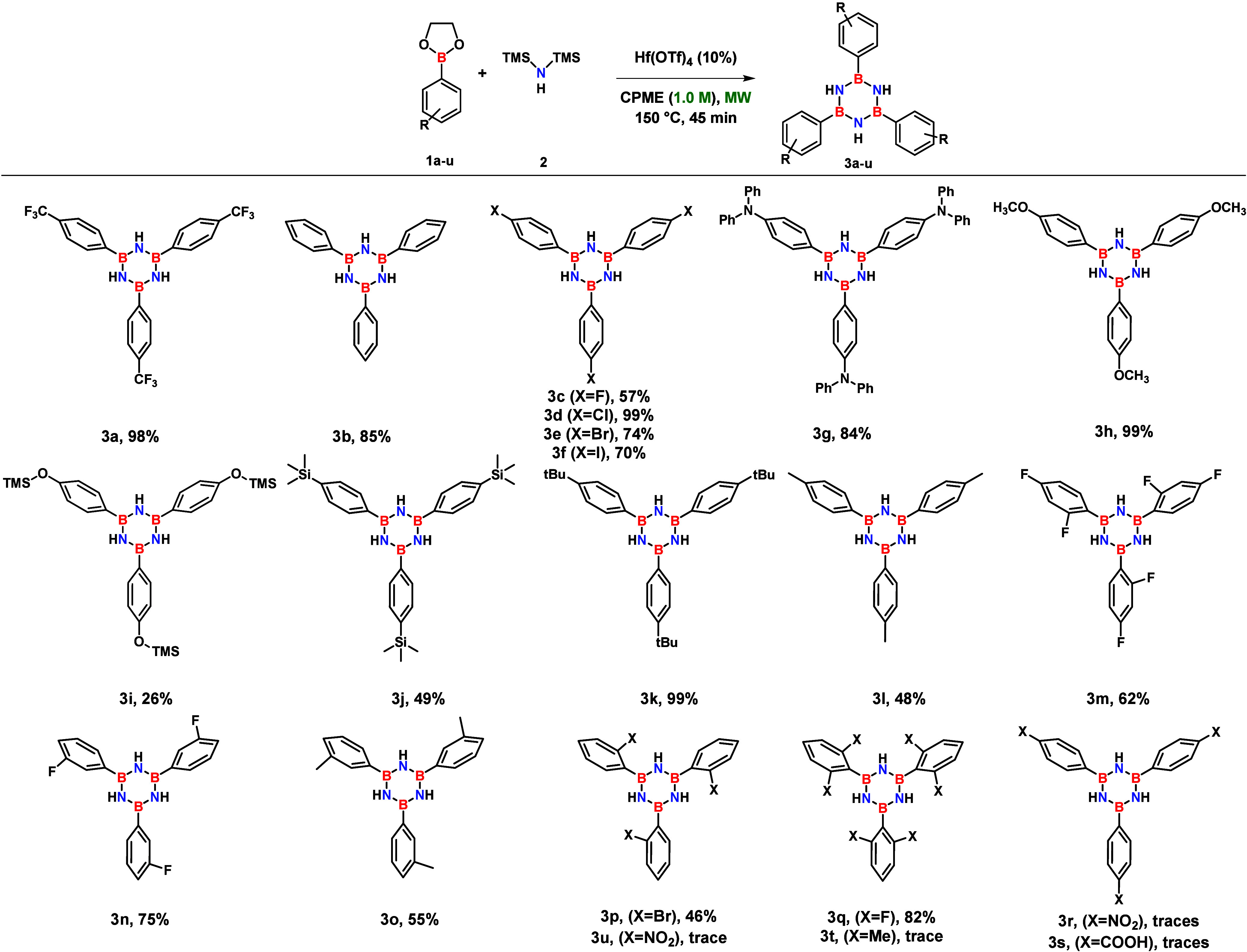
Substrate Scope of the Hf­(OTf)_4_-Catalyzed Synthesis of *N*-H-*B*,*B*′,*B*″-Tri­(aryl)-Substituted Borazines^a^

a Reaction conditions: boronate (**1a**–**1u**, 1 mmol); HMDS (**2**, 1.55 equiv, 0.325 mL), anhydrous CPME (1 mL, 1 M), HfOTf_4_ (10 mol %), 150 °C, 45 min, MW.

Our findings suggest that solubility and steric hindrance are the primary factors governing the efficiency of the process. Substrates with poor solubility in the reaction medium, such as **3r** and **3s**, failed to provide the desired products. Regarding steric effects, while *ortho*-substituents are known to confer higher hydrolytic stability, excessive bulk can inhibit the necessary B–N Lewis interaction. This limitation was confirmed by the failure to synthesize *B*,*B*′,*B*″-tris­(2,6-dimethylphenyl)­borazine (**3t**), a compound that is typically obtained from the functionalization of chloroborazole with aryl lithiums.^33^ Nevertheless, substrates with less demanding *ortho*-substituents, such as **3p** and **3q**, were successfully synthesized, likely due to their reduced steric profile compared to **3t** and **3u**.

In conclusion, the protocol demonstrates broad compatibility with a wide number of functional groups, including aryl halides (−I, −Br, −Cl, −F), ethers (−OCH_3_), and silanes (−TMS). Notably, the reaction with 2-(4-hydroxyphenyl)-1,3,2-dioxaborolane produced *N*-H-*B*,*B*′,*B″*-tris­(4-trimethylsilyloxyphenyl)­borazine (**3i**) rather than *N*-H-*B*,*B*′,*B*″-tris­(4-hydroxyphenyl)­borazine, suggesting an *in situ* silylation by HMDS during the core formation.

## Supplementary Material



## Data Availability

The data underlying this study are available in the published article and its Supporting Information.

## Notes

General remarks, experimental procedures, thermal decomposition screening, heating method optimization, oxophilic transition-metal screening, spectral data, and ^1^H NMR, ^13^C NMR, ^11^B NMR, and ^19^F NMR spectra of isolated compounds (PDF)

## References

[1] Islas R., Chamorro E., Robles J., Heine T., Santos J. C., Merino G. (2007). Borazine: To Be or Not to Be Aromatic. Struct. Chem..

[2] Wu Y., Yan X., Liu Z., Lu T., Zhao M., Xu J., Wang J. (2024). Aromaticity in Isoelectronic
Analogues of Benzene, Carborazine and
Borazine, from Electronic Structure and Magnetic Property. Chem. Eur. J..

[3] Costa A., Costa E. R., Silva A. L. P., Tanaka A. A., De Jesus
Gomes J. (2018). Theoretical Study of the Effects of Substituents (F, Cl, Br, CH3,
and CN) on the Aromaticity of Borazine. J. Mol.
Model..

[4] Chowdhury S., Wakchure V. C., Galleposo E. C., Bonifazi D., Costa R. D. (2025). Cyano-Borazine
Photosensitizers for Dye-Sensitized Solar Cells. Adv. Energy Sustain. Res..

[5] Ibarra-Barreno C. M., Chowdhury S., Crosta M., Zehra T., Fasano F., Kundu P., Verstraelen J., Bals S., Subrati M., Bonifazi D., Costa R. D., Rudolf P. (2025). Bottom-Up Fabrication
of BN-Doped Graphene Electrodes from Thiol-Terminated Borazine Molecules
Working in Solar Cells. ACS Appl. Mater. Interfaces.

[6] Jackson K. T., Rabbani M. G., Reich T. E., El-Kaderi H. M. (2011). Synthesis
of Highly Porous Borazine-Linked Polymers and Their Application to
H2, CO2, and CH4 Storage. Polym. Chem..

[7] Marchionni D., Basak S., Khodadadi A. N., Marrocchi A., Vaccaro L. (2023). Synthesis and Applications of Organic
Borazine Materials. Adv. Funct. Mater..

[8] Fu X., Feng J., Wang Z., Jiang H., Fu X., Feng J., Wang Z., Jiang H. (2025). Synthesis and Functionalization
of Polymeric Materials Based on Organic Borazine. RSC Adv..

[9] Nazari
Khodadadi A., Cela E., Marchionni D., Huang F., Ferlin F., Vaccaro L. (2024). Efficient Access to
Hexaaryl-Substituted Borazines in Batch and Continuous-Flow. Green Chem..

[10] Khodadadi A. N., Cela E., Mosaffa H., Huang F., Marchionni D., Vaccaro L. (2025). Accelerating Rational
Organoborazine Discovery: Predicted
by Machine Learning, Synthesized under Continuous Flow. Green Synth. Catal..

[11] Campana F., Zhou K., Yunda J. A., Khodadadi A. N., Bonifazi D., Melinte S., Vaccaro L. (2025). Assessing
the Energetic
and Environmental Sustainability of Organic Borazines Preparation:
A Comprehensive Life Cycle Assessment and Uncertainty Analysis. Chem. Eng. J..

[12] Marchionni D., Nazari Khodadadi A., Cela E., Huang F., Vaccaro L. (2024). Accessing
Borazine Substitution Patterns through Late-Stage Iodination. Adv. Synth. Catal..

[13] Marchionni D., Gernini D., Nazari Khodadadi A., Cela E., Huang F., Vaccaro L. (2024). A Waste-Minimized Approach
for the Synthesis of Iodinated
Organic Borazines. Green Chem..

[14] Sham I. H.
T., Kwok C.-C., Che C.-M., Zhu N. (2005). Borazine Materials
for Organic Optoelectronic Applications. Chem.
Commun..

[15] Guo K., Qi H., Wang F., Zhu Y. (2014). Borazine-Containing Arylacetylene
Resin as Low Dielectric Constant Materials. Mater. Sci. Eng., B.

[16] Guo K., Qi H., Wang F., Zhu Y. (2014). Fabrication of Boron-
and Nitrogen-Doped
Carbon Nanoparticles by Stress from Pyrolysis of Borazine-Containing
Arylacetylene. RSC Adv..

[17] Chen C., Guo K., Zhu Y., Wang F., Zhang W., Qi H. (2019). Construction
of Layered B 3 N 3 -Doped Graphene Sheets from an Acetylenic Compound
Containing B 3 N 3 by a Semisynthetic Strategy. ACS Appl. Mater. Interfaces.

[18] Chen C., Shen Z., Zhu Y., Wang F., Jiang B., Qi H. (2021). Construction of Activated
Carbon-Supported B 3 N 3 Doped Carbon as
Metal-Free Catalyst for Dehydrochlorination of 1,2-Dichloroethane
to Produce Vinyl Chloride. RSC Adv..

[19] Nöth H., Tinhof W., Taeger T. (1974). Beiträge
zur Chemie des Bors,
LXVII. Silaborazine aus Cyclosilazanen. Chem.
Ber..

[20] Gruzinova E. A., Svatikov M. Yu., Kotrelev G. V., Zhdanov A. A. (1987). Reaction
of Phenylboric
Anhydride with Hexamethylcyclotrisilazane. Bull.
Acad. Sci. USSR Div. Chem. Sci..

[21] Svatikov M. Yu., Gruzinova E. A., Kotrelev G. V. (1991). Reaction of Dibutyl Phenylborate
with Hexamethyldisilazane and Hexamethylcyclotrisilazane. Bull. Acad. Sci. USSR Div. Chem. Sci..

[22] Nöth H., Troll A. (2005). The *N*-Lithiation of 2,4,6-Triphenylborazine. Eur.
J. Inorg. Chem..

[23] Brooks W. L. A., Sumerlin B. S. (2016). Synthesis and Applications
of Boronic Acid-Containing
Polymers: From Materials to Medicine. Chem.
Rev..

[24] Zheng X.-A., Kong R., Huang H.-S., Wei J.-Y., Chen J.-Z., Gong S.-S., Sun Q. (2019). Hafnium Triflate as
a Highly Potent
Catalyst for Regio- and Chemoselective- Deprotection of Silyl Ethers. Synthesis.

[25] Kappe C. O., Pieber B., Dallinger D. (2013). Microwave
Effects in Organic Synthesis:
Myth or Reality?. Angew. Chem., Int. Ed..

[26] Kappe, C. O. ; Dallinger, D. ; Murphree, S. S. Practical Microwave Synthesis for Organic Chemists: Strategies, Instruments, and Protocols, 1st ed.; Wiley, 2008. 10.1002/9783527623907

[27] Raup D. E. A. (2010). Cooperative
Catalysis by Carbenes and Lewis Acids in a Highly Stereoselective
Route to γ-Lactams. Nat. Chem..

[28] Kepp K. P. A. (2016). Quantitative
Scale of Oxophilicity and Thiophilicity. Inorg.
Chem..

[29] Green Solvents for Organic Electronics Processing. In Sustainable Strategies in Organic Electronics; Woodhead Publishing, 2022; pp 425–462. 10.1016/B978-0-12-823147-0.00012-4

[30] Chalid M., Heeres H. J., Broekhuis A. A. (2012). Ring-opening
of Γ-valerolactone
with Amino Compounds. J. Appl. Polym. Sci..

[31] Watanabe K., Yamagiwa N., Torisawa Y. (2007). Cyclopentyl
Methyl Ether as a New
and Alternative Process Solvent. Org. Process
Res. Dev..

[32] Azzena U., Carraro M., Pisano L., Monticelli S., Bartolotta R., Pace V. (2019). Cyclopentyl Methyl Ether: An Elective
Ecofriendly Ethereal Solvent in Classical and Modern Organic Chemistry. ChemSusChem.

[33] Miyaura, N. ; Yamamoto, Y. Preparation of B-arylborazines for applications in semiconductor devices. JP2009-137319, December 16, 2010.

