# Supplementary material for: Expanding the Structural Diversity of N–H Borazines: A Hf(OTf)4‑Catalyzed Synthesis from Aryl Boronates under Microwave Irradiation
Source: Org Lett. 2026 May 11;28(20):6393–8. doi: 10.1021/acs.orglett.6c01514 (PMC13200235; doi:10.1021/acs.orglett.6c01514)
Supplement: Supplementary file 1 [file ol6c01514_si_001.pdf]

SUPPORTING INFORMATION FILE

Expanding the Structural Diversity of *N*–H Borazines: A Hf(OTf)$_4$-Catalyzed Synthesis from Aryl Boronates under Microwave Irradiation

Ejdi Cela,[a]† Alireza Nazari Khodadadi,[a]† Fan Huang,[a] Dario Marchionni,[a] Luigi Vaccaro*[a]

[a] *Laboratory of Green S.O.C. - Dipartimento di Chimica, Biologia e Biotecnologie, Università degli Studi di Perugia, Via Elce di Sotto 8, 06123 - Perugia, Italy. http://www.dcbb.unipg.it/greensoc.*

E-mail: luigi.vaccaro@unipg.it

Table of Contents

1. General remarks: 2

2. General procedure: Synthesis of differently substituted *N*-H-*B,B',B"*-tri(aryl) substituted borazines. 3

3. Optimization of reaction conditions 3

4. Synthesis and characterization of products 3a-3q 5

4. References 25

5. Copies of NMR spectra of isolated products 25

LIST OF ABBREVIATION:

Et_2O	Diethyl Ether
HMDS	Hexamethyldisilazane
THF	Tetrahydrofuran
Ar	Argon
MW	Microwave

1. General remarks:

Unless otherwise stated, chemicals were purchased from Merck, TCI and Fluorochem and used as obtained from commercial sources without further purification.

GLC analyses were performed using an Agilent 6850 Series GC System equipped with a BB-5MS capillary column (30 m, 0.32 mm), FID detector and helium as the gas carrier. GC-EIMS analyses were carried out using an Agilent 9890 GC System/5977C MSD equipped with an electron impact ionizer at 70 eV.

Nuclear magnetic resonance (NMR) spectra were recorded on a Bruker DRX-ADVANCE 400 MHz spectrometer (^{1}H at 400 MHz,^{13}C at 100.6 MHz, ^{19}F at 376.4 MHz, and ^{11}B NMR spectra at 128 MHz) using the solvent residual signal as an internal reference ($CDCl_3$: δH = 7.26 ppm, δC = 77.16 ppm and DMSO: δH = 2.50 ppm, δC = 39.7 ppm). Boron chemical shifts are reported in ppm, referenced to the external standard boron signal of $BF_3 \cdot Et_2O$ (δB = 0 ppm). Chemical shifts are reported in ppm (δ), coupling constant (*J*) in hertz (Hz) and multiplicity is abbreviated as follows: s = singlet, bs = broad singlet, d = doublet, dd = double of doublets, td = triplet of doublets, t = triplet, t = triplet of triplets, m = multiplet.

The elemental composition of borazines was determined using Elementar UNICUBE® elemental analyzer.

Microwave-assisted reactions were performed in a CEM Discover SP microwave oven using 10 mL vessels in sealed reaction vessel mode, the temperature was monitored via an external surface IR sensor. The "Dynamic" method was applied with the following parameters: T = 150°C, t = 1 h, μλ = 150 W, Pressure = 300 psi, Mixing = high, PowerMax = On.

Boronic esters (**1a-1q**) were synthesized according to literature available procedures.[1] New compounds (**1f, 1k, 1m, 1q**) were fully characterized.

Solvents were dried over activated molecular sieves (4Å, 20% w/w) for 3 days.

All catalysts were dried at 110°C overnight before use.

2. General procedure: Synthesis of differently substituted *N*-H-*B,B',B"*-tri(aryl) substituted borazines.

In an oven-dried MW vessel, after applying vacuum and successively Ar, the substituted boronic ester (1 mmol, 1.0 eq) and dry $Hf(OTf)_4$ (10 mol%, 0.1 eq, 77 mg) were placed. The vessel was then evacuated and backfilled with argon. Subsequently, anhydrous CPME (1 mL, 1.0 M) and HMDS (**2**, 1.55 mmol, 1.55 eq, 325 μL) were added via syringe under argon. The reaction vessel was placed in the MW synthesizer and irradiated at 150°C for 45 min. After cooling to room temperature, the volatile components were removed under reduced pressure. The resulting solid was treated with Et_2O and stirred for 1 h at room temperature to ensure product solubilization. The catalyst was removed by filtration and the filtrate was concentrated under reduced pressure. The pure product was obtained by precipitation from hexane.

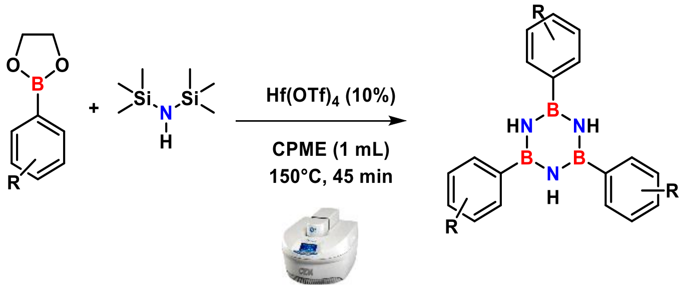

1a-q 2 3a-q

Scheme S1. General procedure for the synthesis of *N*-H-*B,B',B"*-tri(aryl) substituted borazines.

3. Optimization of reaction conditions

Table S1. Screening of thermal decomposition conditions. [a]

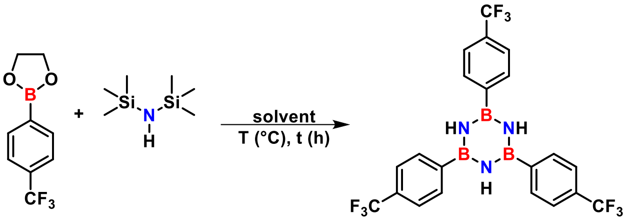

1a 2 3a

#	Amine/Boron	Solvent	Temp. (°C)	Time (h)	Yield (%)[b]
1	1.0	Toluene	110	24	-
2	6.2	Toluene	150	72	-
3	6.2	CPME	150	72	-
4	14.32	-	150	72	<5

[a]Reaction conditions: Boronate (**1a**, 1 mmol); solvent 2 mL. [b]Isolated yields

Table S2. Evaluation of oxophilic Lewis acid catalysts and heating method. [a]

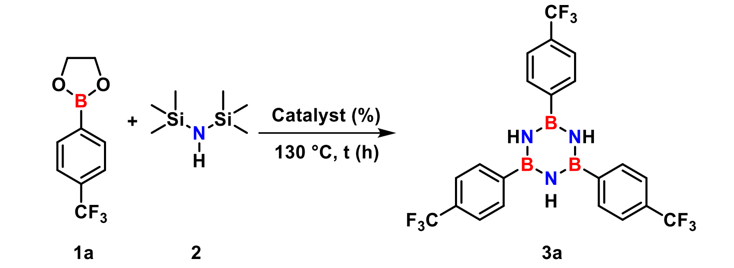

#	Catalyst (30%)	Heating	Temp. (°C)	Time (h)	Yield (%)[b]
1[d]	$TiCl_4$	Oil bath	130	72	15
2[d]	$TiCl_4$	Oil bath	130	49[c]	29
3	$HfCl_4$	Oil bath	130	72	20
4	$Hf(OTf)_4$	Oil bath	130	72	56
5	$Hf(OTf)_4$ (15%)	Oil bath	130	72	51
6	$Hf(OTf)_4$ (15%)	Oil bath	130	48	40
7	$Hf(OTf)_4$ (15%)	Oil bath	130	24	27
8	$Hf(OTf)_4$ (15%)	Oil bath	130	7	17
9	$Hf(OTf)_4$ (15%)	MW	150	7	75
10	$Hf(OTf)_4$	MW	150	5	83
11	$Hf(OTf)_4$	MW	150	3	32

[a]Reaction conditions: Boronate (**1a**, 1 mmol); HMDS (**2**, 14.32 eq, 3 mL). [b]Isolated yields [c]Reaction performed in autoclave [d]$TiCl_4$ is in DCM solution.

Table S3. Screening of oxophilic transition-metal catalysts under MW irradiation. [a]

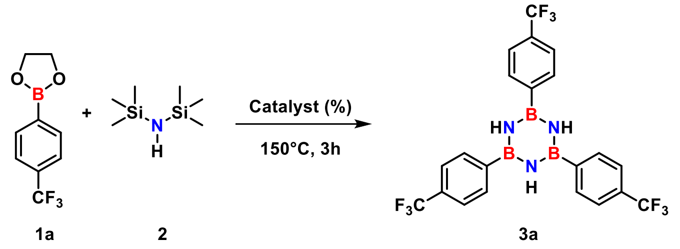

#	Catalyst (30 mol%)	Oxophilicity (Θ)	HMDS (**2**)	Yield (%)[b]
1	$Hf(OTf)_4$	1.0	14.32 eq	32
2	$CpTiCl_3$	1.0	14.32 eq	Traces
3	$Sc(OTf)_3$	0.8	14.32 eq	20
4	$Sc(OTf)_3$	0.8	4.80 eq	4
5	Yb_2O_3	0.9	14.32 eq	0
6	$ZrCl_4$	0.8	14.32 eq	15
7	$C_9H_{30}O_4Si_3V$ [c]	0.8	14.32 eq	0
8	$Mg(OTf)_2$	0.6	14.32 eq	7
9	$In(OTf)_3$	0.4	14.32 eq	0
10	$Cu(OTf)_2$	0.2	14.32 eq	Traces
11	AgOTf	0.2	14.32 eq	6

[a]Reaction conditions: Boronate (**1a**, 1 mmol), 150°C, 3 h, MW [b]Isolated yields [c]The product couldn't be isolated

4. Synthesis and characterization of products 3a-3q

Chem. Name	*N*-H-*B,B′,B″*-tri(4-(trifluoromethyl)phenyl)borazine (3a)

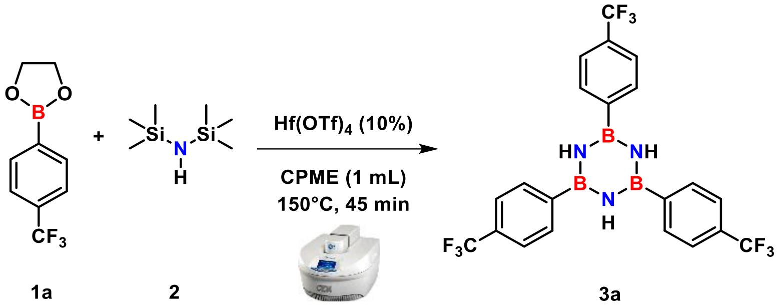

METHOD: In an oven-dried MW vessel, after applying vacuum and successively Ar, 2-(4-(trifluoromethyl)phenyl)-1,3,2-dioxaborolane (**1a**, 1 mmol, 1.0 eq, 216 mg) and dry $Hf(OTf)_4$ (10 mol%, 0.1 eq, 77 mg) were placed. The vessel was then evacuated and backfilled with argon. Subsequently, anhydrous CPME (1 mL, 1 M) and HMDS (**2**, 1.55 mmol, 1.55 eq, 325 μL) were added via syringe under argon. The reaction vessel was placed in the MW synthesizer and irradiated at 150°C for 45 min. After cooling to room temperature, the volatile components were removed under reduced pressure. The resulting solid was treated with Et_2O and stirred for 1 h at room temperature to ensure product solubilization. The catalyst was removed by filtration and the filtrate was concentrated under reduced pressure. The pure product was obtained by precipitation from hexane.
166 mg, isolated yield 98%, white powder

**Mol Formula**	$C_{21}H_{15}B_3N_3F_9$	**m.p.=** 244°C

^{1}H NMR ($CDCl_3$, 400 MHz): δ 7.88 (d, *J*= 7.8 Hz, 6H), 7.73 (d, *J*= 7.8 Hz, 6H), 5.94 (s, 3H).

^{13}C NMR ($CDCl_3$, 100.6 MHz): δ 132.2, 132.1 (q, $^{2}J_{C-F}$=32.8 Hz), 124.9 (q, $^{3}J_{C-F}$ = 3.8 Hz), 124.1 (q, $^{1}J_{C-F}$=272.2 Hz). The signal for the carbon atom attached to boron atom was not detected because of the quadrupolar relaxation of the boron atom.

^{11}B NMR ($CDCl_3$,128 MHz): δ 33.42

^{19}F NMR ($CDCl_3$, 376.4 MHz): δ -62.88

Anal. Calcd. for ($C_{21}H_{15}B_3N_3F_9$): C, 49.19%; H, 2.95%; N, 8.19%. **Found:** C, 49.03%; H, 2.85%; N, 8.05%.

Chem. Name	***N*-H-*B,B′,B″*-tri(phenyl)borazine (3b)**

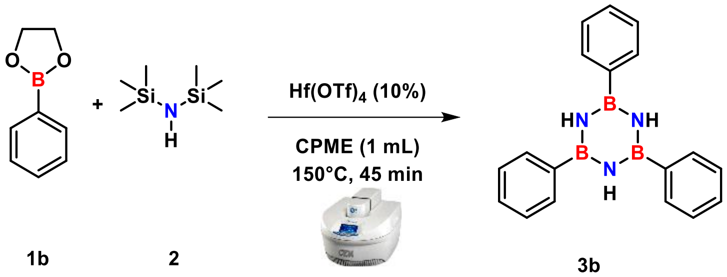

METHOD: In an oven-dried MW vessel, after applying vacuum and successively Ar, 2-phenyl-1,3,2-dioxaborolane (**1b**, 1 mmol, 1.0 eq, 148 mg) and dry $Hf(OTf)_4$ (10 mol%, 0.1 eq, 77 mg) were placed. The vessel was then evacuated and backfilled with argon. Subsequently, anhydrous CPME (1 mL, 1 M) and HMDS (**2**, 1.55 mmol, 1.55 eq, 325 µL) were added via syringe under argon. The reaction vessel was placed in the MW synthesizer and irradiated at 150°C for 45 min. After cooling to room temperature, the volatile components were removed under reduced pressure. The resulting solid was treated with Et_2O and stirred for 1 h at room temperature to ensure product solubilization. The catalyst was removed by filtration and the filtrate was concentrated under reduced pressure. The pure product was obtained by precipitation from hexane.
87 mg, isolated yield 85%, white powder

**Mol Formula**	$C_{18}H_{18}B_3N_3$	**m.p.=** 175°C

^{1}**H NMR** ($CDCl_3$, 400 MHz): δ 7.78-7.80 (m, 6H), 7.47-7.48 (m, 9H), 5.91 (s, 3H).

^{13}**C NMR** ($CDCl_3$, 100.6 MHz): δ 132.1, 130.1, 128.3. The signal for the carbon atom attached to boron atom was not detected because of the quadrupolar relaxation of the boron atom.

^{11}**B NMR** ($CDCl_3$, 128 MHz): δ 33.55

Anal. Calcd. for ($C_{18}H_{18}B_3N_3$): C, 70.01%; H, 5.88%; N, 13.61%. **Found:** C, 69.87%; H, 5.91%; N, 13.51%.

Chem. Name	***N*-H-*B,B',B''*-tri(4-fluorophenyl)borazine (3c)**

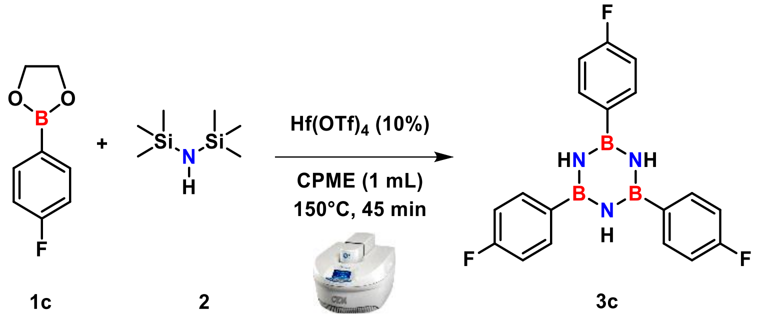

METHOD: In an oven-dried MW vessel, after applying vacuum and successively Ar, 2-(4-fluorophenyl)-1,3,2-dioxaborolane (**1c**, 1 mmol, 1.0 eq, 166 mg) and dry $Hf(OTf)_4$ (10 mol%, 0.1 eq, 77 mg) were placed. The vessel was then evacuated and backfilled with argon. Subsequently, anhydrous CPME (1 mL, 1 M) and HMDS (**2**, 1.55 mmol, 1.55 eq, 325 µL) were added via syringe under argon. The reaction vessel was placed in the MW synthesizer and irradiated at 150°C for 45 min. After cooling to room temperature, the volatile components were removed under reduced pressure. The resulting solid was treated with Et_2O and stirred for 1 h at room temperature to ensure product solubilization. The catalyst was removed by filtration and the filtrate was concentrated under reduced pressure. The pure product was obtained by precipitation from hexane.
68 mg, isolated yield 57%, white powder

**Mol Formula**	$C_{18}H_{15}B_3F_3N_3$	**m.p.=** 269°C

^{1}H NMR ($CDCl_3$, 400 MHz): δ 7.77-7.74 (m, 6H), 7.18-7.14 (m, 6H), 5.78 (s, 3H).

^{13}C NMR ($CDCl_3$, 100.6 MHz): δ 164.5 (d, $^1J_{C\text{-}F}$=249.24Hz), 134.0 (d, $^3J_{C\text{-}F}$=7.82Hz), 115.4 (d, $^2J_{C\text{-}F}$=20.13Hz). The signal for the carbon atom attached to boron atom was not detected because of the quadrupolar relaxation of the boron atom.

^{11}B NMR ($CDCl_3$, 128 MHz): δ 33.03

^{19}F NMR ($CDCl_3$, 376.4 MHz): δ -110.56

Anal. Calcd. for ($C_{18}H_{15}B_3N_3F_3$): C, 59.60%; H, 4.17%; N, 11.58%. **Found:** C, 59.51%; H, 4.12%; N, 11.52%.

Chem. Name	***N*-H-*B,B',B''*-tri(4-chlorophenyl)borazine (3d)**

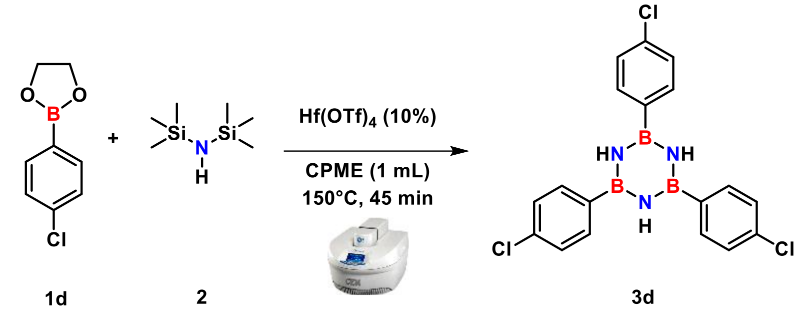

1d 2 3d

METHOD: In an oven-dried MW vessel, after applying vacuum and successively Ar, 2-(4-chlorophenyl)-1,3,2-dioxaborolane (**1d**, 1 mmol, 1.0 eq, 182.4 mg) and dry $Hf(OTf)_4$ (10 mol%, 0.1 eq, 77 mg) were placed. The vessel was then evacuated and backfilled with argon. Subsequently, anhydrous CPME (1 mL, 1 M) and HMDS (**2**, 1.55 mmol, 1.55 eq, 325 µL) were added via syringe under argon. The reaction vessel was placed in the MW synthesizer and irradiated at 150°C for 45 min. After cooling to room temperature, the volatile components were removed under reduced pressure. The resulting solid was treated with Et_2O and stirred for 1 h at room temperature to ensure product solubilization. The catalyst was removed by filtration and the filtrate was concentrated under reduced pressure. The pure product was obtained by precipitation from hexane.
135 mg, isolated yield 99%, white powder

**Mol Formula**	$C_{18}H_{15}B_3Cl_3N_3$	**m.p.=** 260°C

^{1}H NMR ($CDCl_3$, 400 MHz): δ 7.69 (d, *J*=8.3 Hz, 6H), 7.44 (d, *J*=8.2 Hz, 6H), 5.80 (s, 3H).

^{13}C NMR ($CDCl_3$, 100.6 MHz): δ 136.5, 133.4, 128.6. The signal for the carbon atom attached to boron atom was not detected because of the quadrupolar relaxation of the boron atom.

^{11}B NMR ($CDCl_3$, 128 MHz): δ 33.39

Anal. Calcd. for ($C_{18}H_{15}B_3N_3Cl_3$): C, 52.46%; H, 3.67%; N, 10.20%. **Found:** C, 52.42%; H, 3.61%; N, 10.16%.

Chem. Name	*N*-H-*B,B′,B″*-tri(4-bromophenyl)borazine (3e)

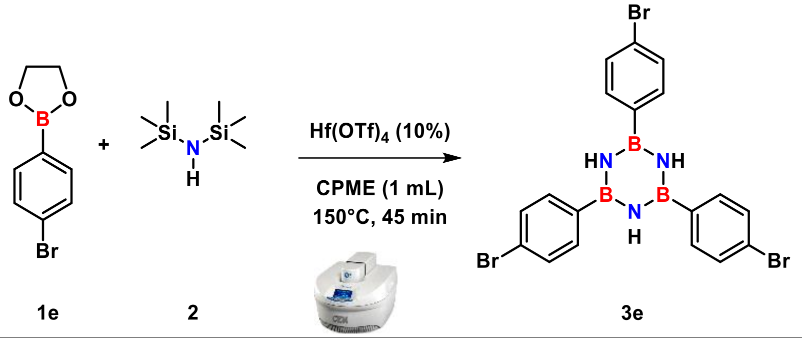

METHOD: In an oven-dried MW vessel, after applying vacuum and successively Ar, 2-(4-bromophenyl)-1,3,2-dioxaborolane (**1e**, 1 mmol, 1.0 eq, 227 mg) and dry $Hf(OTf)_4$ (10 mol%, 0.1 eq, 77 mg) were placed. The vessel was then evacuated and backfilled with argon. Subsequently, anhydrous CPME (1 mL, 1 M) and HMDS (**2**, 1.55 mmol, 1.55 eq, 325 µL) were added via syringe under argon. The reaction vessel was placed in the MW synthesizer and irradiated at 150°C for 45 min. After cooling to room temperature, the volatile components were removed under reduced pressure. The resulting solid was treated with Et_2O and stirred for 1 h at room temperature to ensure product solubilization. The catalyst was removed by filtration and the filtrate was concentrated under reduced pressure. The pure product was obtained by precipitation from hexane.

134 mg, isolated yield 74%, white powder

**Mol Formula**	$C_{18}H_{15}B_3Br_3N_3$	**m.p.=** 275°C

^{1}H NMR ($CDCl_3$, 400 MHz): δ 7.63-7.59 (m, 12H), 5.80 (s, 3H).

^{13}C NMR ($CDCl_3$, 100.6 MHz): δ 133.6, 131.5, 125.0. The signal for the carbon atom attached to boron atom was not detected because of the quadrupolar relaxation of the boron atom.

^{11}B NMR ($CDCl_3$**,** 128 MHz): δ 33.09

Anal. Calcd. for ($C_{18}H_{15}B_3N_3Br_3$): C, 39.63%; H, 2.77%; N, 7.70%. **Found:** C, 39.59%; H, 2.74%; N, 7.65%.

<table>
<tr><td>Chem. Name</td><td colspan="3">2-(4-iodophenyl)-1,3,2-dioxaborolane (1f)</td></tr>
<tr><td colspan="4">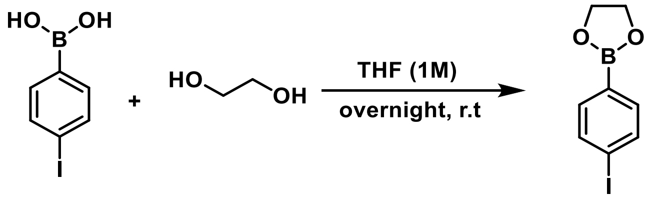

1f</td></tr>
<tr><td colspan="4">METHOD: In an oven-dried round bottom flask were added 5 mmol of 4-iodophenylboronic acid (1.239 g) and 4 mL of dry THF. After solubilization ethylene glycol (17.88 mmol,1 mL) was added and the reaction mixture was allowed to stir overnight at room temperature. Afterwards, the mixture was concentrated under reduced pressure and 5 mL of diethyl ether were added. The resulting solution was transferred to 1 g of dry $CaCl_2$ powder, and after filtration and evaporation under vacuum, the product (1f) was collected.
1.394 g, 99% isolated yield, pale brown solid</td></tr>
<tr><td>Mol Formula</td><td>$C_8H_8BIO_2$</td><td colspan="2">m.p.= 100°C</td></tr>
<tr><td colspan="4">^{1}H NMR ($CDCl_3$, 400 MHz): δ 7.74 (d, J=8.09 Hz, 2H), 7.52 (d, J=8.13 Hz, 2H), 4.38 (s, 4H).</td></tr>
<tr><td colspan="4">^{13}C NMR ($CDCl_3$, 100.6 MHz): δ 137.2, 136.4, 99.2, 66.2. The signal for the carbon atom attached to boron atom was not detected because of the quadrupolar relaxation of the boron atom.</td></tr>
<tr><td colspan="4">^{11}B NMR ($CDCl_3$, 128 MHz): δ 31.65</td></tr>
<tr><td colspan="4">GC-EIMS (m/z, %): 275 (M+ +1, 19), 274 (M+, 100), 273 (M+ -1, 41), 217 (13), 1147.1 (42), 146.1 (11), 121 (19), 117.1 (14), 103.1 (49), 77.1 (31), 76.1 (11)</td></tr>
</table>

**Chem. Name**	***N*-H-*B,B′,B″*-tri(4-iodophenyl)borazine (3f)**

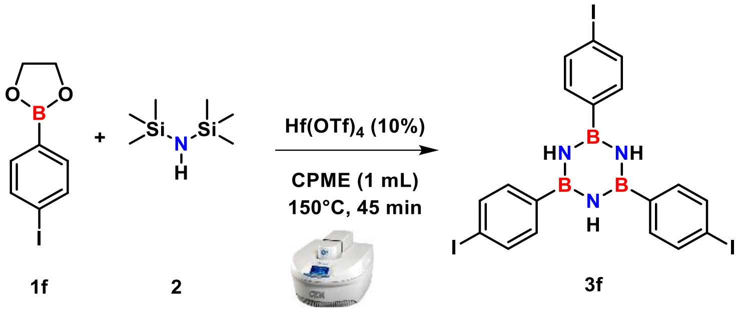

1f 2 3f

METHOD: In an oven-dried MW vessel, after applying vacuum and successively Ar, 2-(4-iodophenyl)-1,3,2-dioxaborolane (**1f**, 1 mmol, 1.0 eq, 166 mg) and dry $Hf(OTf)_4$ (10 mol%, 0.1 eq, 77 mg) were placed. The vessel was then evacuated and backfilled with argon. Subsequently, anhydrous CPME (1 mL, 1 M) and HMDS (**2**, 1.55 mmol, 1.55 eq, 325 µL) were added via syringe under argon. The reaction vessel was placed in the MW synthesizer and irradiated at 150°C for 45 min. After cooling to room temperature, the volatile components were removed under reduced pressure. The resulting solid was treated with Et_2O and stirred for 1 h at room temperature to ensure product solubilization. The catalyst was removed by filtration and the filtrate was concentrated under reduced pressure. The pure product was obtained by precipitation from hexane.
160 mg, isolated yield 70%, white powder

**Mol Formula**	$C_{18}H_{15}B_3I_3N_3$	**m.p.=** 260°C

^{1}H NMR ($CDCl_3$, 400 MHz): δ 7.81 (d, *J*=8.04 Hz, 6H), 7.47 (d, *J*=7.97 Hz, 6H), 5.79 (s, 3H).

^{13}C NMR ($CDCl_3$, 100.6 MHz): δ 137.5, 133.7, 97.2. The signal for the carbon atom attached to boron atom was not detected because of the quadrupolar relaxation of the boron atom.

^{11}B NMR ($CDCl_3$, 128 MHz): δ 33.05

Anal. Calcd. for ($C_{18}H_{15}B_3N_3I_3$): C, 31.49%; H, 2.20%; N, 6.12%. **Found:** C, 31.42%; H, 2.15%; N, 6.08%.

Chem. Name	***N*-H-*B,B′,B″*-tri(4- diphenylaminophenyl)borazine (3g)**

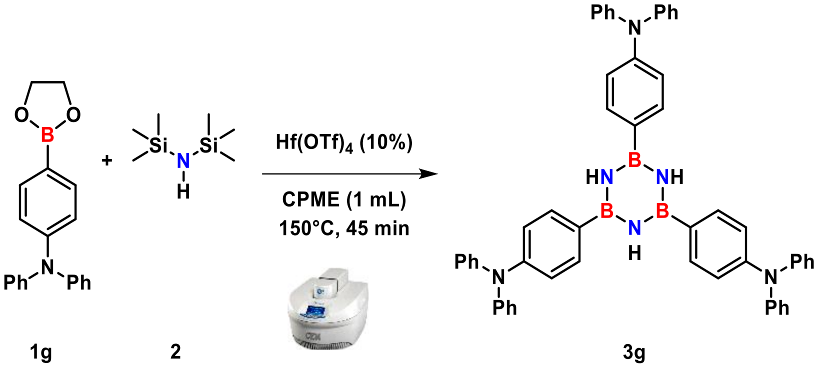

METHOD: In an oven-dried MW vessel, after applying vacuum and successively Ar, 2-(4-(diphenylamino)phenyl)-1,3,2-dioxaborolane (**1g**, 1 mmol, 1.0 eq, 315 mg) and dry $Hf(OTf)_4$ (10 mol%, 0.1 eq, 77 mg) were placed. The vessel was then evacuated and backfilled with argon. Subsequently, anhydrous CPME (1 mL, 1 M) and HMDS (**2**, 1.55 mmol, 1.55 eq, 325 μL) were added via syringe under argon. The reaction vessel was placed in the MW synthesizer and irradiated at 150°C for 45 min. After cooling to room temperature, the volatile components were removed under reduced pressure. The resulting solid was treated with Et_2O and stirred for 1 h at room temperature to ensure product solubilization. The catalyst was removed by filtration and the filtrate was concentrated under reduced pressure. The pure product was obtained by precipitation from hexane.
224 mg, isolated yield 84%, white powder

**Mol Formula**	$C_{54}H_{45}B_3N_6$	**m.p.=** 268°C

^{1}H NMR ($CDCl_3$, 400 MHz): δ 7.63 (d, *J*=8.39 Hz, 6H), 7.29-7.25 (m, 12H), 7.14-7.03 (m, 24H), 5.75 (s, 3H).

^{13}C NMR ($CDCl_3$, 100.6 MHz): δ 149.6, 147.6, 133.1, 129.4, 124.9, 123.3, 122.6. The signal for the carbon atom attached to boron atom was not detected because of the quadrupolar relaxation of the boron atom.

Anal. Calcd. for ($C_{54}H_{45}B_3N_6$): C, 80.03%; H, 5.60%; N, 10.37%. **Found:** C, 79.98%; H, 5.65%; N, 10.35%.

Chem. Name	*N*-H-*B,B',B''*-tri(4-methoxyphenyl)borazine (3h)

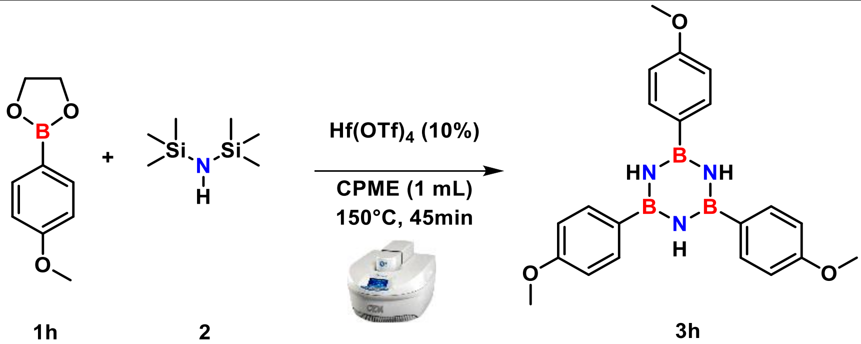

METHOD: In an oven-dried MW vessel, after applying vacuum and successively Ar, 2-(4-methoxyphenyl)-1,3,2-dioxaborolane (**1h**, 1 mmol, 1.0 eq, 178 mg) and dry $Hf(OTf)_4$ (10 mol%, 0.1 eq, 77 mg) were placed. The vessel was then evacuated and backfilled with argon. Subsequently, anhydrous CPME (1 mL, 1 M) and HMDS (**2**, 1.55 mmol, 1.55 eq, 325 µL) were added via syringe under argon. The reaction vessel was placed in the MW synthesizer and irradiated at 150°C for 45 min. After cooling to room temperature, the volatile components were removed under reduced pressure. The resulting solid was treated with Et_2O and stirred for 1 h at room temperature to ensure product solubilization. The catalyst was removed by filtration and the filtrate was concentrated under reduced pressure. The pure product was obtained by precipitation from hexane.
131 mg, isolated yield 99%, white powder

**Mol Formula**	$C_{21}H_{24}B_3N_3O_3$	**m.p.=** 160°C

^{1}H NMR ($CDCl_3$, 400 MHz): δ 7.73 (d, *J*=8.55 Hz, 6H), 7.00 (d, *J*=8.6 Hz, 6H), 5,75 (s, 3H), 3.87 (s, 9H).

^{13}C NMR ($CDCl_3$, 100.6 MHz): δ 161.3, 133.6, 113.8, 55.3. The signal for the carbon atom attached to boron atom was not detected because of the quadrupolar relaxation of the boron atom.

^{11}B NMR ($CDCl_3$, 128 MHz): δ 33.04

Anal. Calcd. for ($C_{21}H_{24}B_3N_3O_3$): C, 63.24%; H, 6.06%; N, 10.53%; O, 12.03%. **Found:** C, 63.20%; H, 6.09%; N, 10.49%; O, 12.00%.

Chem. Name	***N*-H-*B,B′,B″*-tri(4-trimethylsilyloxyphenyl)borazine (3i)**

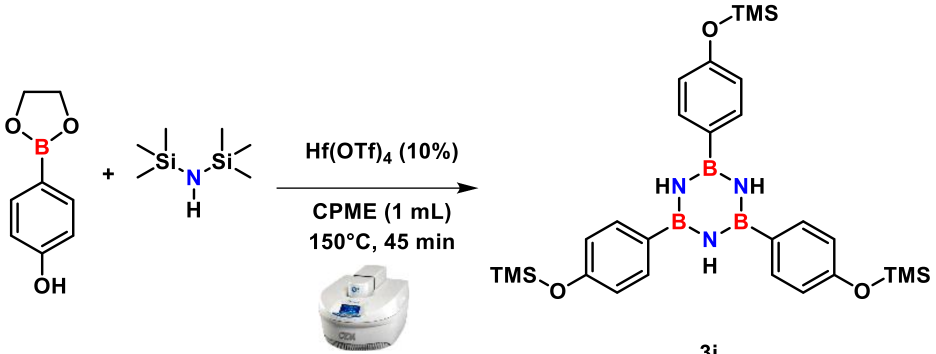

1i **2** **3i**

METHOD: In an oven-dried MW vessel, after applying vacuum and successively Ar, 2-(4-hydroxyphenyl)-1,3,2-dioxaborolane (**1i**, 1 mmol, 1.0 eq, 164 mg) and dry $Hf(OTf)_4$ (10 mol%, 0.1 eq, 77 mg) were placed. The vessel was then evacuated and backfilled with argon. Subsequently, anhydrous CPME (1 mL, 1 M) and HMDS (**2**, 1.55 mmol, 1.55 mmol, 325 μL) were added via syringe under argon. The reaction vessel was placed in the MW synthesizer and irradiated at 150°C for 45 min. After cooling to room temperature, the volatile components were removed under reduced pressure. The resulting solid was treated with Et_2O and stirred for 1 h at room temperature to ensure product solubilization. The catalyst was removed by filtration and the filtrate was concentrated under reduced pressure. The pure product was obtained by precipitation from hexane.
50 mg, isolated yield 26%, white powder

**Mol Formula**	$C_{27}H_{42}B_3N_3O_3Si_3$	**m.p.=** 170°C

^{1}H NMR ($CDCl_3$, 400 MHz): δ 7.67 (d, *J*=8.31 Hz, 6H), 6.93 (d, *J*=8.29 Hz, 6H), 5,74 (s, 3H), 0.30 (s, 27H)

^{13}C NMR ($CDCl_3$, 100.6 MHz): δ 157.2, 133.6, 120.0, 0.4. The signal for the carbon atom attached to boron atom was not detected because of the quadrupolar relaxation of the boron atom.

^{11}B NMR ($CDCl_3$**,** 128 MHz): δ 32.95

Anal. Calcd. for ($C_{27}H_{42}B_3N_3O_3Si_3$): C, 56.56%; H, 7.38%; N, 7.33%; O, 8.37%. **Found:** C, 56.49%; H, 7.33%; N, 7.48%; O, 8.35%.

Chem. Name	***N*-H-*B,B′,B″*-tri(4-trimethylsilylphenyl)borazine (3j)**

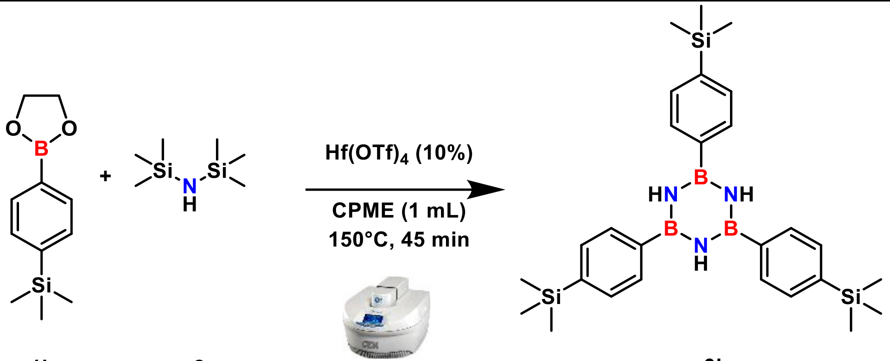

1j **2** **3j**

METHOD: In an oven-dried MW vessel, after applying vacuum and successively Ar, 2-(4-(trimethylsilyl)phenyl)-1,3,2-dioxaborolane (**1*J***, 1 mmol, 1.0 eq, 220 mg) and dry $Hf(OTf)_4$ (10 mol%, 0.1 eq, 77 mg) were placed. The vessel was then evacuated and backfilled with argon. Subsequently, anhydrous CPME (1 mL, 1 M) and HMDS (**2**, 1.55 mmol, 1.55 eq, 325 μL) were added via syringe under argon. The reaction vessel was placed in the MW synthesizer and irradiated at 150°C for 45 min. After cooling to room temperature, the volatile components were removed under reduced pressure. The resulting solid was treated with Et_2O and stirred for 1 h at room temperature to ensure product solubilization. The catalyst was removed by filtration and the filtrate was concentrated under reduced pressure. The pure product was obtained by precipitation from hexane.
85 mg, isolated yield 49%, pale-brown powder

**Mol Formula**	$C_{27}H_{42}B_3N_3Si_3$	**m.p.=** 185°C

^{1}H NMR ($CDCl_3$, 400 MHz): δ 7.75 (d, *J*=7.4 Hz, 6H), 7.63 (d, *J*=7.4 Hz, 6H), 5.89 (s, 3H), 0.31 (s, 9H).

^{13}C NMR ($CDCl_3$, 100.6 MHz): δ 142.7, 133.2, 131.3, -1.0. The signal for the carbon atom attached to boron atom was not detected because of the quadrupolar relaxation of the boron atom.

^{11}B NMR ($CDCl_3$**,** 128 MHz): δ 33.59

Anal. Calcd. for ($C_{27}H_{42}B_3N_3Si_3$): C, 61.73%; H, 8.06%; N, 8.00%. **Found:** C, 61.47%; H, 8.01%; N, 7.97%.

Chem. Name	2-(4-tert-butylphenyl)-1,3,2-dioxaborolane (1k)

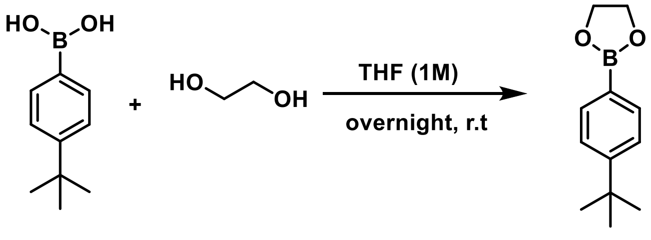

1k

METHOD: In an oven-dried round bottom flask were added 5 mmol of 4-tert-butylphenylboronic acid (890 mg) and 4 mL of dry THF. After solubilization ethylene glycol (17.88 mmol,1 mL) was added and the reaction mixture was allowed to stir overnight at room temperature. Afterwards, the mixture was concentrated under reduced pressure and 5 mL of diethyl ether were added. The resulting solution was transferred to 1 g of dry $CaCl_2$ powder and, after filtration and evaporation under vacuum, the product (**1k**) was collected
863 mg, 85% isolated yield, white solid

**Mol Formula**	$C_{12}H_{17}BO_2$	**m.p.=** 80°C

^{1}H NMR ($CDCl_3$, 400 MHz): δ 7.76 (d, *J*=8.2 Hz, 6H), 7.42 (d, *J*=8.2 Hz, 6H), 4.37 (s, 4H), 1.33 (s, 9H).

^{13}C NMR ($CDCl_3$, 100.6 MHz): δ 154.8, 134.9, 124.9, 66.1, 35.0, 31.3. The signal for the carbon atom attached to boron atom was not detected because of the quadrupolar relaxation of the boron atom.

^{11}B NMR ($CDCl_3$, 128 MHz): δ 31.63

GC-EIMS (m/z, %): 204.1 (M+, 20), 190.1 (17), 189.1 (100), 188.1 (22), 184 (11), 161.1 (25), 117.1 (20), 105.1 (13), 91.1 (26)

Chem. Name	***N*-H-*B,B',B''*-tri(4-tert-butylphenyl)borazine (3k)**

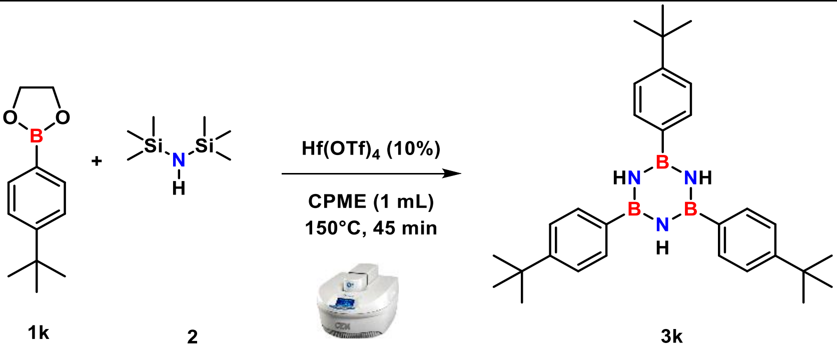

1k **2** **3k**

METHOD: In an oven-dried MW vessel, after applying vacuum and successively Ar, 2-(4-tert-butylphenyl)-1,3,2-dioxaborolane (**1k**, 1 mmol, 1.0 eq, 204 mg) and dry $Hf(OTf)_4$ (10 mol%, 0.1 eq, 77 mg) were placed. The vessel was then evacuated and backfilled with argon. Subsequently, anhydrous CPME (1 mL, 1 M) and HMDS (**2**, 1.55 mmol, 1.55 eq, 325 µL) were added via syringe under argon. The reaction vessel was placed in the MW synthesizer and irradiated at 150°C for 45 min. After cooling to room temperature, the volatile components were removed under reduced pressure. The resulting solid was treated with Et_2O and stirred for 1 h at room temperature to ensure product solubilization. The catalyst was removed by filtration and the filtrate was concentrated under reduced pressure. The pure product was obtained by precipitation from hexane.

158 mg, isolated yield 99%, white powder

**Mol Formula**	$C_{30}H_{42}B_3N_3$	**m.p.=** 230°C

^{1}H NMR ($CDCl_3$, 400 MHz): δ 7.72 (d, *J*= 8.0 Hz, 6H), 7.50 (d, *J*= 8.1 Hz, 6H), 5.85 (s, 3H), 1.37 (s, 27H).

^{13}C NMR ($CDCl_3$, 100.6 MHz): δ 153.2, 131.9, 125.9, 34.9, 31.4. The signal for the carbon atom attached to boron atom was not detected because of the quadrupolar relaxation of the boron atom.

^{11}B NMR ($CDCl_3$, 128 MHz): δ 33.39

Anal. Calcd. for ($C_{30}H_{42}B_3N_3$): C, 75.52%; H, 8.87%; N, 8.81%. **Found:** C, 75.49%; H, 8.82%; N, 8.77%.

Chem. Name	*N*-H-*B,B′,B″*-tri(4-methylphenyl)borazine (3l)

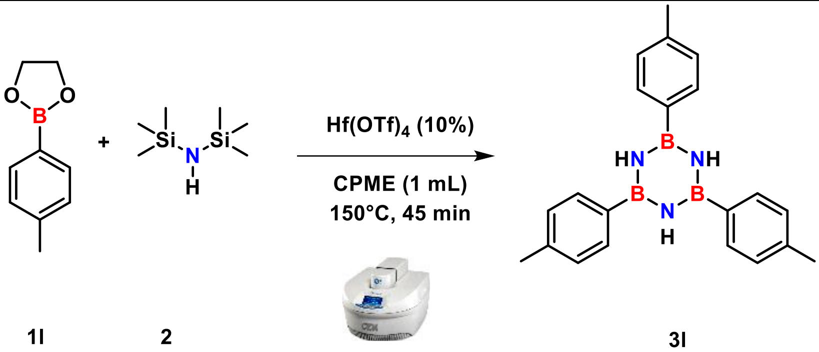

1l 2 3l

METHOD: In an oven-dried MW vessel, after applying vacuum and successively Ar, 2-(4-methylphenyl)-1,3,2-dioxaborolane (**1l**, 1 mmol, 1.0 eq, 162 mg) and dry $Hf(OTf)_4$ (10 mol%, 0.1 eq, 77 mg) were placed. The vessel was then evacuated and backfilled with argon. Subsequently, anhydrous CPME (1 mL, 1 M) and HMDS (**2**, 1.55 mmol, 1.55 eq, 325 µL) were added via syringe under argon. The reaction vessel was placed in the MW synthesizer and irradiated at 150°C for 45 min. After cooling to room temperature, the volatile components were removed under reduced pressure. The resulting solid was treated with Et_2O and stirred for 1 h at room temperature to ensure product solubilization. The catalyst was removed by filtration and the filtrate was concentrated under reduced pressure. The pure product was obtained by precipitation from hexane.
56 mg, isolated yield 48%, white powder

**Mol Formula**	$C_{21}H_{24}B_3N_3$	**m.p.=** 197°C

^{1}H NMR ($CDCl_3$, 400 MHz): δ 7.69 (d, *J*=7.65 Hz, 6H), 7.28 (d, *J*=7.56 Hz, 6H), 5.84 (s, 3H), 2.41 (s, 9H).

^{13}C NMR ($CDCl_3$, 100.6 MHz): δ 140.1, 132.1, 129.1, 21.7. The signal for the carbon atom attached to boron atom was not detected because of the quadrupolar relaxation of the boron atom.

^{11}B NMR ($CDCl_3$, 128 MHz): δ 33.41

Anal. Calcd. for ($C_{21}H_{24}B_3N_3$): C, 71.89%; H, 6.89%; N, 11.98%. **Found:** C, 71.85%; H, 6.86%; N, 11.95%.

Chem. Name	**2-(2,4-difluorophenyl)-1,3,2-dioxaborolane (1m)**

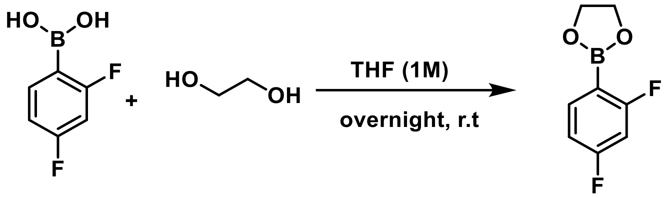

1m

METHOD: In an oven-dried round bottom flask were added 5 mmol of 2,4-diflurophenylboronic acid (790 mg) and 4 mL of dry THF. After solubilization ethylene glycol (17.88 mmol,1 mL) was added and the reaction mixture was allowed to stir overnight at room temperature. Afterwards, the mixture was concentrated under reduced pressure and 5 mL of diethyl ether were added. The resulting solution was transferred to 1 g of dry $CaCl_2$ powder and, after filtration and evaporation under vacuum, the product (**1m**) was collected.
809 mg, 88% isolated yield, white solid

**Mol Formula**	$C_8H_7BF_2O_2$	**m.p.=** 90°C

^{1}**H NMR** ($CDCl_3$, 400 MHz): δ 7.78-7.72 (m, 1H), 6.92-6.87 (m, 1H), 6.82-6.77 (m, 1H), 4.40 (s, 4H).

^{13}**C NMR** ($CDCl_3$, 100.6 MHz): δ 168.3 (dd, $^1J_{C-F}$=225.6 Hz, $^4J_{C-F}$=12.3 Hz), 165.7 (dd, $^1J_{C-F}$=225.3 Hz, $^4J_{C-F}$=12.2 Hz), 138.4 (t, $^2J_{C-F}$=9.9 Hz), 111.5 (dd, $^3J_{C-F}$=20.4 Hz, $^5J_{C-F}$=3.5 Hz), 104.0 (dd,$^2J_{C-F}$=27.9 Hz, $^2J_{C-F}$=24.4 Hz), 66.1. The signal for the carbon atom attached to boron atom was not detected because of the quadrupolar relaxation of the boron atom.

^{11}**B NMR** ($CDCl_3$, 128 MHz): δ 30.67

^{19}**F NMR** ($CDCl_3$, 376.4 MHz): δ -99.04, -104.37

GC-EIMS (m/z, %): 185.1 (M+ +1, 12), 184 (M+, 100), 183 (M + -1, 46), 166 (38), 165.1 (16), 154 (28), 153 (69), 152 (10), 147.1 (37), 136 (20), 135 (18), 127 (29), 125 (22), 124 (14), 109 (46), 108 (42), 107 (29), 94 (13), 75 (15), 74 (13), 73 859), 71.1 (10), 57.1 (12), 51 (12)

Chem. Name	***N*-H-*B,B′,B″*-tri(2,4- difluorophenyl)borazine (3m)**

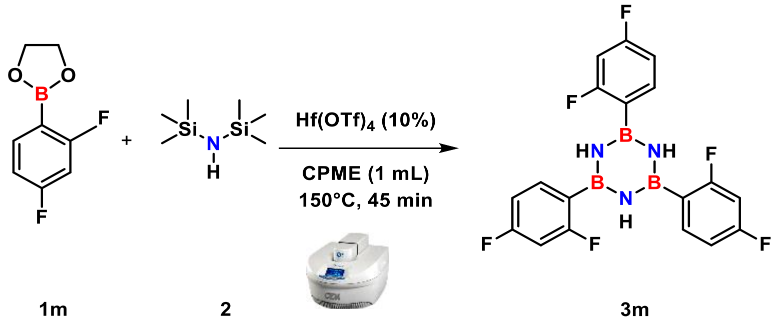

1m **2** **3m**

METHOD: In an oven-dried MW vessel, after applying vacuum and successively Ar, 2-(2,4-difluorophenyl)-1,3,2-dioxaborolane (**1m**, 1 mmol, 1.0 eq, 184 mg) and dry $Hf(OTf)_4$ (10 mol%, 0.1 eq, 77 mg) were placed. The vessel was then evacuated and backfilled with argon. Subsequently, anhydrous CPME (1 mL, 1 M) and HMDS (**2**, 1.55 mmol, 1.55 eq, 325 μL) were added via syringe under argon. The reaction vessel was placed in the MW synthesizer and irradiated at 150°C for 45 min. After cooling to room temperature, the volatile components were removed under reduced pressure. The resulting solid was treated with Et_2O and stirred for 1 h at room temperature to ensure product solubilization. The catalyst was removed by filtration and the filtrate was concentrated under reduced pressure. The pure product was obtained by precipitation from hexane.
85 mg, isolated yield 62%, off-white powder

**Mol Formula**	$C_{18}H_{12}B_3F_6N_3$	**m.p.=** 210°C

^{1}H NMR ($CDCl_3$, 400 MHz): δ 7.68-7.62 (m, 3H), 7.01-6.96 (m, 3H), 6.89-6.84 (m, 3H), 6.29 (s, 3H).

^{11}B NMR ($CDCl_3$, 128 MHz): δ 31.69

^{19}F NMR ($CDCl_3$, 376.4 MHz): δ -106.89, -103.05

Anal. Calcd. for ($C_{18}H_{12}B_3N_3F_6$): C, 51.88%; H, 2.90%; N, 10.08%. **Found:** C, 51.82%; H, 2.92%; N, 10.01%.

Chem. Name	***N*-H-*B,B′,B″*-tri(3-fluorophenyl)borazine (3n)**

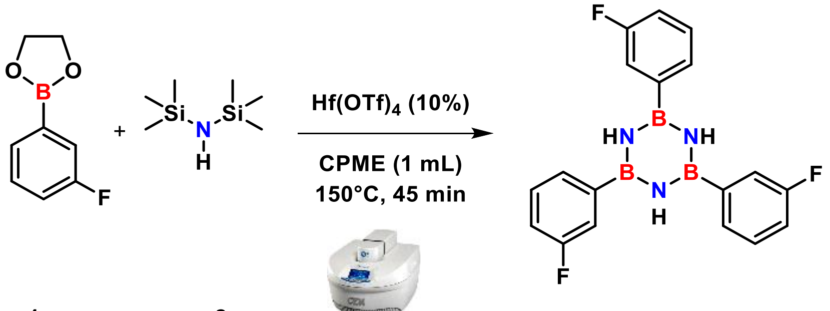

1n 2 3n

METHOD: In an oven-dried MW vessel, after applying vacuum and successively Ar, 2-(3-fluorophenyl)-1,3,2-dioxaborolane (**1n**, 1 mmol, 1.0 eq, 166 mg) and dry $Hf(OTf)_4$ (10 mol%, 0.1 eq, 77 mg) were placed. The vessel was then evacuated and backfilled with argon. Subsequently, anhydrous CPME (1 mL, 1 M) and HMDS (**2**, 1.55 mmol, 1.55 eq, 325 µL) were added via syringe under argon. The reaction vessel was placed in the MW synthesizer and irradiated at 150°C for 45 min. After cooling to room temperature, the volatile components were removed under reduced pressure. The resulting solid was treated with Et_2O and stirred for 1 h at room temperature to ensure product solubilization. The catalyst was removed by filtration and the filtrate was concentrated under reduced pressure. The pure product was obtained by precipitation from hexane.
90 mg, isolated yield 75%, off-white powder

**Mol Formula**	$C_{18}H_{15}B_3F_3N_3$	**m.p.=** 190°C

^{1}H NMR ($CDCl_3$, 400 MHz): δ 7.54-7.52 (m, 3H), 7.48-7.41 (m, 6H), 7.19-7.14 (m, 3H), 5.84 (s, 3H).

^{13}C NMR ($CDCl_3$, 100.6 MHz): δ 163.1 (d, $^1J_{C\text{-}F}$= 247.4 Hz), 130.2 (d, $^4J_{C\text{-}F}$ = 7.3 Hz), 127.6 (d, $^3J_{C\text{-}F}$ = 2.9 Hz), 118.4 (d, $^2J_{C\text{-}F}$ = 18.9 Hz), 117.1 (d, $^2J_{C\text{-}F}$ = 21.1 Hz). The signal for the carbon atom attached to boron atom was not detected because of the quadrupolar relaxation of the boron atom.

^{11}B NMR ($CDCl_3$**,** 128 MHz): δ 33.19

^{19}F NMR ($CDCl_3$, 376.4 MHz): δ -113.38

Anal. Calcd. for ($C_{18}H_{15}B_3N_3F_3$): C, 59.60%; H, 4.17%; N, 11.58%. **Found:** C, 59.51%; H, 4.12%; N, 11.52%.

Chem. Name	***N*-H-*B,B',B''*-tri(m-tolyl)borazine (3o)**

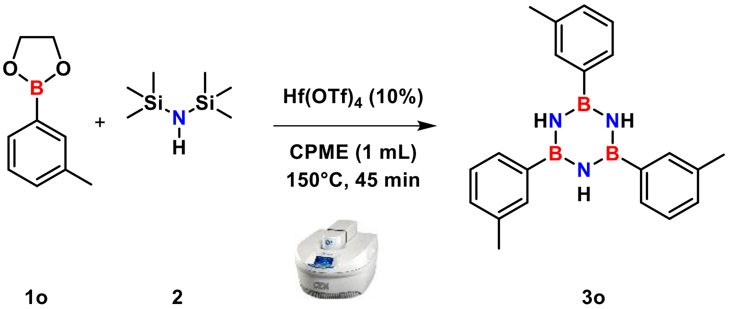

1o **2** **3o**

METHOD: In an oven-dried MW vessel, after applying vacuum and successively Ar, 2-(m-tolyl)-1,3,2-dioxaborolane (**1o**, 1 mmol, 1.0 eq, 162 mg) and dry Hf(OTf)$_4$ (10 mol%, 0.1 eq, 77 mg) were placed. The vessel was then evacuated and backfilled with argon. Subsequently, anhydrous CPME (1 mL, 1 M) and HMDS (**2**, 1.55 mmol, 1.55 eq, 325 μL) were added via syringe under argon. The reaction vessel was placed in the MW synthesizer and irradiated at 150°C for 45 min. After cooling to room temperature, the volatile components were removed under reduced pressure. The resulting solid was treated with Et$_2$O and stirred for 1 h at room temperature to ensure product solubilization. The catalyst was removed by filtration and the filtrate was concentrated under reduced pressure. The pure product was obtained by precipitation from hexane.
64 mg, isolated yield 55%, white powder

**Mol Formula**	$C_{21}H_{24}B_3N_3$	**m.p.=** 140°C

^{1}H NMR ($CDCl_3$, 400 MHz): δ 7.59 (s, 6H), 7.39-7.29 (m, 6H), 5.87 (s, 3H), 2.45 (s, 9H).

^{13}C NMR ($CDCl_3$, 100.6 MHz): δ 137.6, 132.8, 130.8, 129.1, 128.2, 21.7. The signal for the carbon atom attached to boron atom was not detected because of the quadrupolar relaxation of the boron atom.

^{11}B NMR ($CDCl_3$, 128 MHz): δ 33.59

Anal. Calcd. for ($C_{21}H_{24}B_3N_3$): C, 71.89%; H, 6.89%; N, 11.98%. **Found:** C, 71.85%; H, 6.87%; N, 11.95%.

Chem. Name	***N*-H-*B,B',B''*-tri(2-bromophenyl)borazine (3p)**

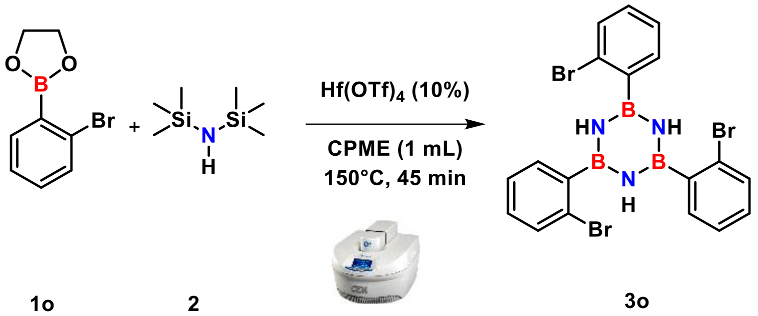

1o 2 3o

METHOD: In an oven-dried MW vessel, after applying vacuum and successively Ar, 2-(2-bromophenyl)-1,3,2-dioxaborolane (**1p**, 1 mmol, 1.0 eq, 227 mg) and dry $Hf(OTf)_4$ (10 mol%, 0.1 eq, 77 mg) were placed. The vessel was then evacuated and backfilled with argon. Subsequently, anhydrous CPME (1 mL, 1 M) and HMDS (**2**, 1.55 mmol, 1.55 eq, 325 μL) were added via syringe under argon. The reaction vessel was placed in the MW synthesizer and irradiated at 150°C for 45 min. After cooling to room temperature, the volatile components were removed under reduced pressure. The resulting solid was treated with Et_2O and stirred for 1 h at room temperature to ensure product solubilization. The catalyst was removed by filtration and the filtrate was concentrated under reduced pressure. The pure product was obtained by precipitation from hexane.
83 mg, isolated yield 46%, white powder

**Mol Formula**	$C_{18}H_{15}B_3Br_3N_3$	

^{1}H NMR ($CDCl_3$, 400 MHz): δ 7.60 7.58 (m, 3H), 7.56-7.54 (m 3H), 7.37-7.33 (m, 3H), 7.27-7.23 (m, 3H), 5.99 (s, 3H)

^{13}C NMR ($CDCl_3$, 100.6 MHz): δ 134.6, 132.8, 130.8, 127.0, 126.9. The signal for the carbon atom attached to boron atom was not detected because of the quadrupolar relaxation of the boron atom.

^{11}B NMR ($CDCl_3$, 128 MHz): δ 33.77

Anal. Calcd. for ($C_{18}H_{15}B_3N_3Br_3$): C, 39.63%; H, 2.77%; N, 7.70%. **Found:** C, 39.59%; H, 2.74%; N, 7.65%.

Chem. Name	2-(2,6-difluorophenyl)-1,3,2-dioxaborolane (1q)

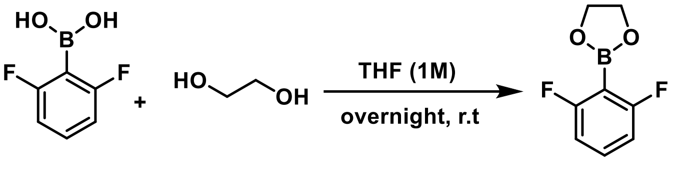

METHOD: In an oven-dried round bottom flask were added 5 mmol of 2,6-dilfuorophenylboronic acid (790 mg) and 4 mL of dry THF. After solubilization ethylene glycol (17.88 mmol,1 mL) was added and the reaction mixture was allowed to stir overnight at room temperature. Afterwards, the mixture was concentrated under reduced pressure and 5 mL of diethyl ether were added. The resulting solution was transferred to 1 g of dry $CaCl_2$ powder and, after filtration and evaporation under vacuum, the product (**1q**) was collected.
641 mg, 70% isolated yield, white solid

**Mol Formula**	$C_8H_7BF_2O_2$	**m.p.=** 55°C

^{1}H NMR ($CDCl_3$, 400 MHz): δ 7.44-7.37 (m, 1H), 6.90-6.86 (m, 2H), 4.43 (s, 4H).

^{13}C NMR ($CDCl_3$, 100.6 MHz): δ 167.2 (dd, $^1J_{C-F}$= 252.5 Hz, $^3J_{C-F}$ = 12.3 Hz), 133.9 (t, $^3J_{C-F}$ = 10.9 Hz), 111.4 (m), 66.1. The signal for the carbon atom attached to boron atom was not detected because of the quadrupolar relaxation of the boron atom.

^{11}B NMR ($CDCl_3$, 128 MHz): δ 30.25

^{19}F NMR ($CDCl_3$, 376.4 MHz): δ -99.92

GC-EIMS (m/z, %): 185.1 (M+ +1, 15), 184.1 (M+, 100), 183.1 (M+ -1, 73), 182.1 (11), 154.1 (19), 153.1 (78), 152 (17), 127 (13), 125 (37), 124 (12), 109.1 (15), 108.1 (32), 107.1 (22), 98 (13), 57.1 (11), 50.1 (11)

Chem. Name	*B,B',B''*-tri(2,6-difluorophenyl)borazine (3q)

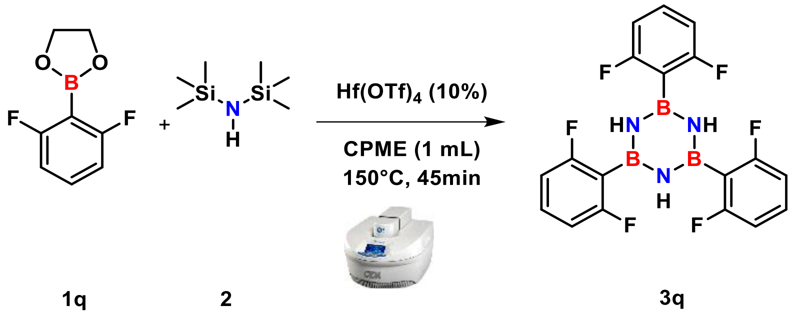

1q 2 3q

METHOD: In an oven-dried MW vessel, after applying vacuum and successively Ar, 2-(2,6-difluorophenyl)-1,3,2-dioxaborolane (**1q**, 1 mmol, 1.0 eq, 184 mg) and dry $Hf(OTf)_4$ (10 mol%, 0.1 eq, 77 mg) were placed. The vessel was then evacuated and backfilled with argon. Subsequently, anhydrous CPME (1 mL, 1 M) and HMDS (**2**, 1.55 mmol, 1.55 eq, 325 μL) were added via syringe under argon. The reaction vessel was placed in the MW synthesizer and irradiated at 150°C for 45 min. After cooling to room temperature, the volatile components were removed under reduced pressure. The resulting solid was treated with Et_2O and stirred for 1 h at room temperature to ensure product solubilization. The catalyst was removed by filtration and the filtrate was concentrated under reduced pressure. The pure product was obtained by precipitation from hexane.
113 mg, isolated yield 82%, white powder

**Mol Formula**	$C_{18}H_{12}B_3F_6N_3$

^{1}H NMR ($CDCl_3$, 400 MHz): δ 7.42 7.34 (m, 3H), 6.96-6.90 (m, 6H), 5.38 (s, 3H, br).

^{13}C NMR ($CDCl_3$, 100.6 MHz): δ 167.2 (dd, $^1J_{C\text{-}F}$= 247.0 Hz, $^3J_{C\text{-}F}$ = 13.2 Hz), 132.3 (t, $^3J_{C\text{-}F}$ = 11.7 Hz), 111.7 (m). The signal for the carbon atom attached to boron atom was not detected because of the quadrupolar relaxation of the boron atom.

^{11}B NMR ($CDCl_3$, 128 MHz): δ 30.93

^{19}F NMR ($CDCl_3$, 376.4 MHz): δ -103.79

GC-EIMS (m/z, %): 418.27 (M+ +1, 32), 417.28 (M+, 100), 416.29 (M+ -1, 90), 415.31 (32), 323.17 (28), 322.18 (28), 304.18 (28), 303.18 (24), 208.93 (23), 208.29 (25), 188.1 (12).

Anal. Calcd. for ($C_{18}H_{12}B_3N_3F_6$): C, 51.88%; H, 2.90%; N, 10.08%. **Found:** C, 51.82%; H, 2.92%; N, 10.01%.

4. References

(1) RanJani, G.; NagaraJan, R. Insight into Copper Catalysis: In Situ Formed Nano Cu $_2$ O in Suzuki–Miyaura Cross-Coupling of Aryl/Indolyl Boronates. *Org. Lett.* **2017**, *19* (15), 3974–3977.

5. Copies of NMR spectra of isolated products

***N*-H-*B,B′,B″*-tri(4-(trifluoromethyl)phenyl)borazine**

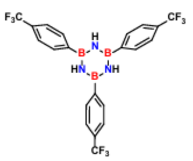

3a, 98%

^{1}H NMR ($CDCl_3$, 400 MHz)

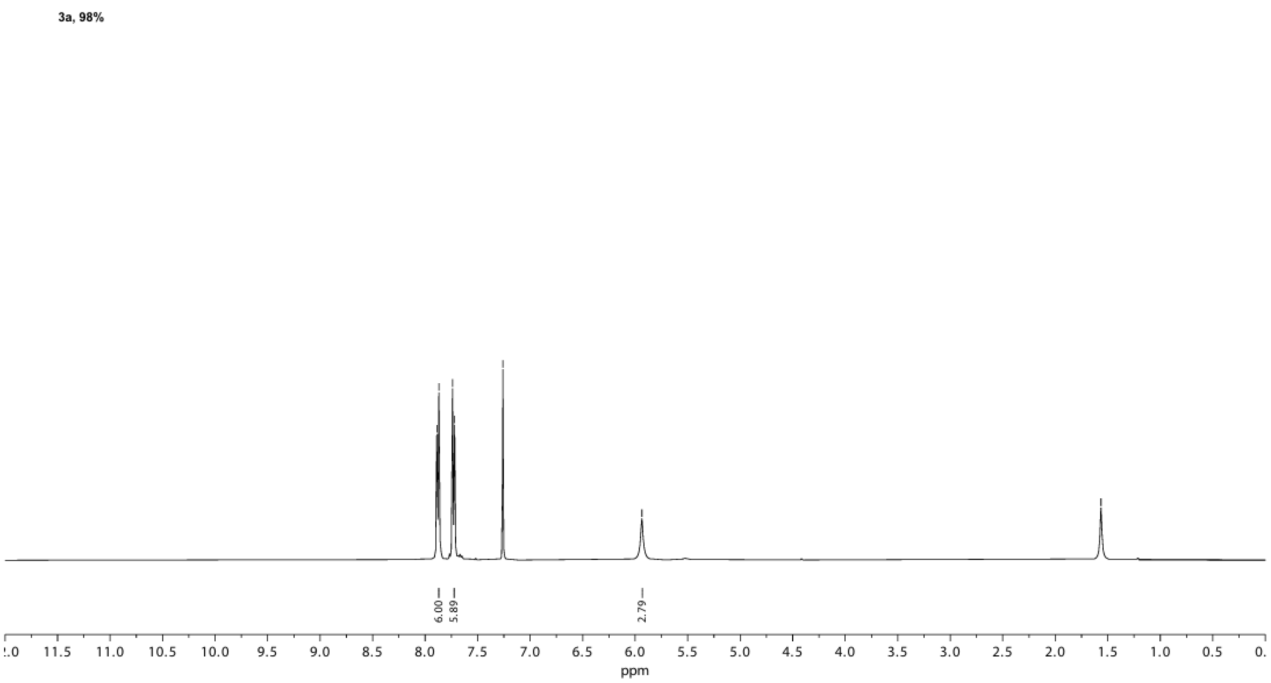

***N*-H-*B,B′,B″*-tri(4-(trifluoromethyl)phenyl)borazine**

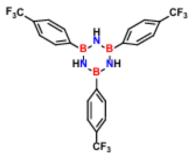

3a, 98%

^{11}B NMR ($CDCl_3$**,** 128 MHz)

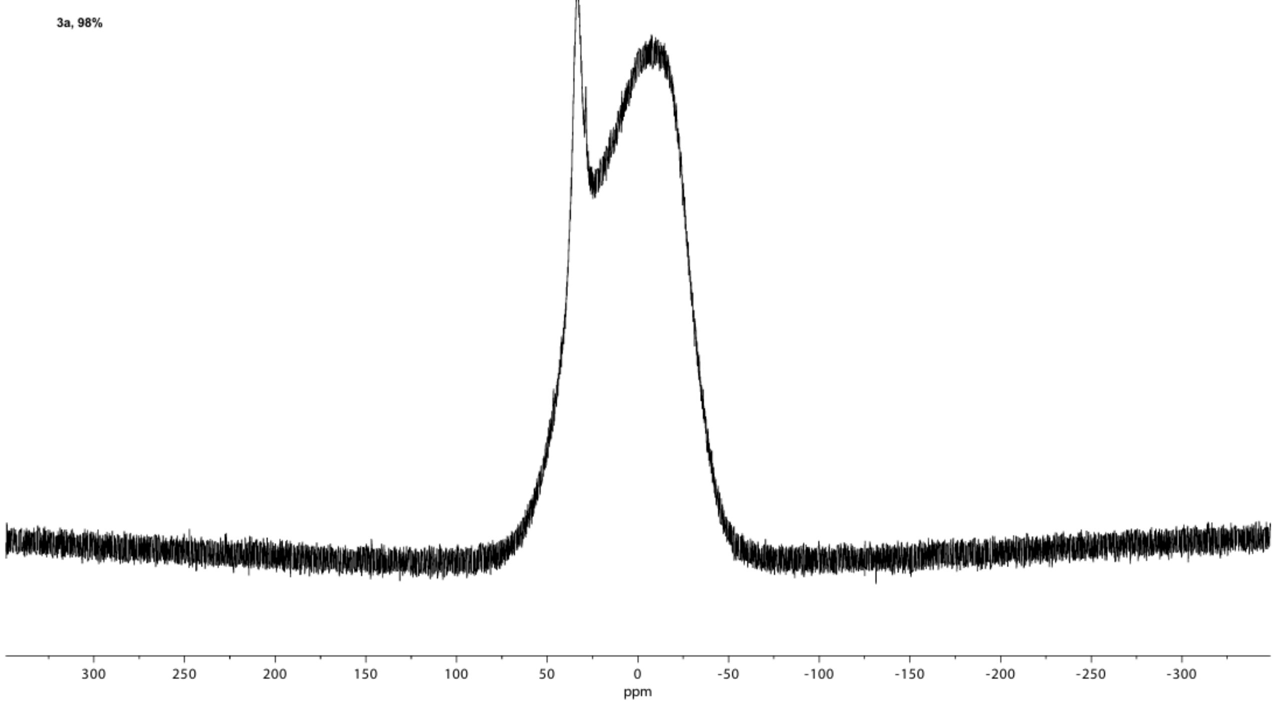

***N*-H-*B,B′,B″*-tri(4-(trifluoromethyl)phenyl)borazine**

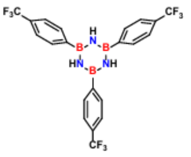

3a, 98%

^{13}C NMR (CDCl$_3$, 100.6 MHz)

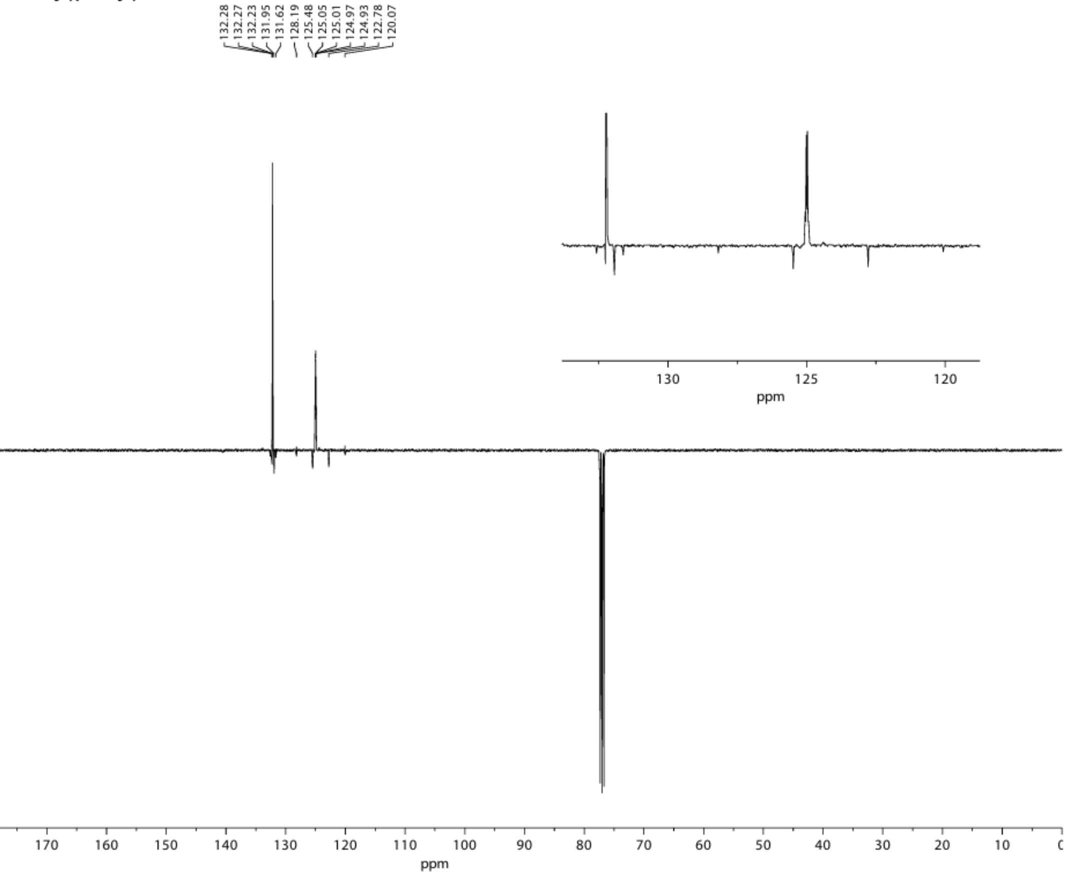

***N*-H-*B,B′,B″*-tri(4-(trifluoromethyl)phenyl)borazine**

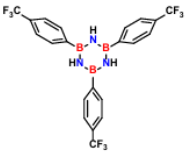

3a, 98%

^{19}F NMR (CDCl$_3$, 376.4 MHz)

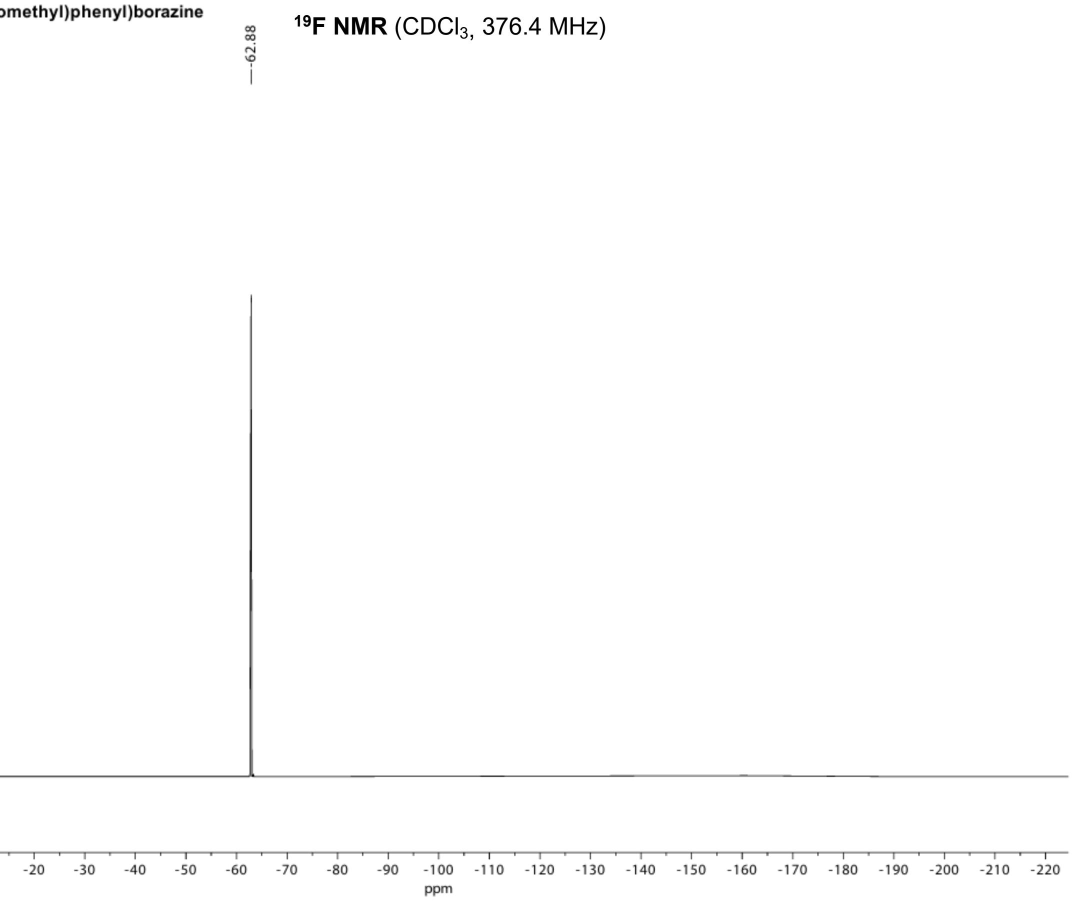

N-H-*B,B′,B′′*-tri(phenyl)borazine

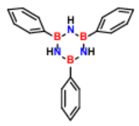

3b, 85%

^{1}H NMR ($CDCl_3$, 400 MHz)

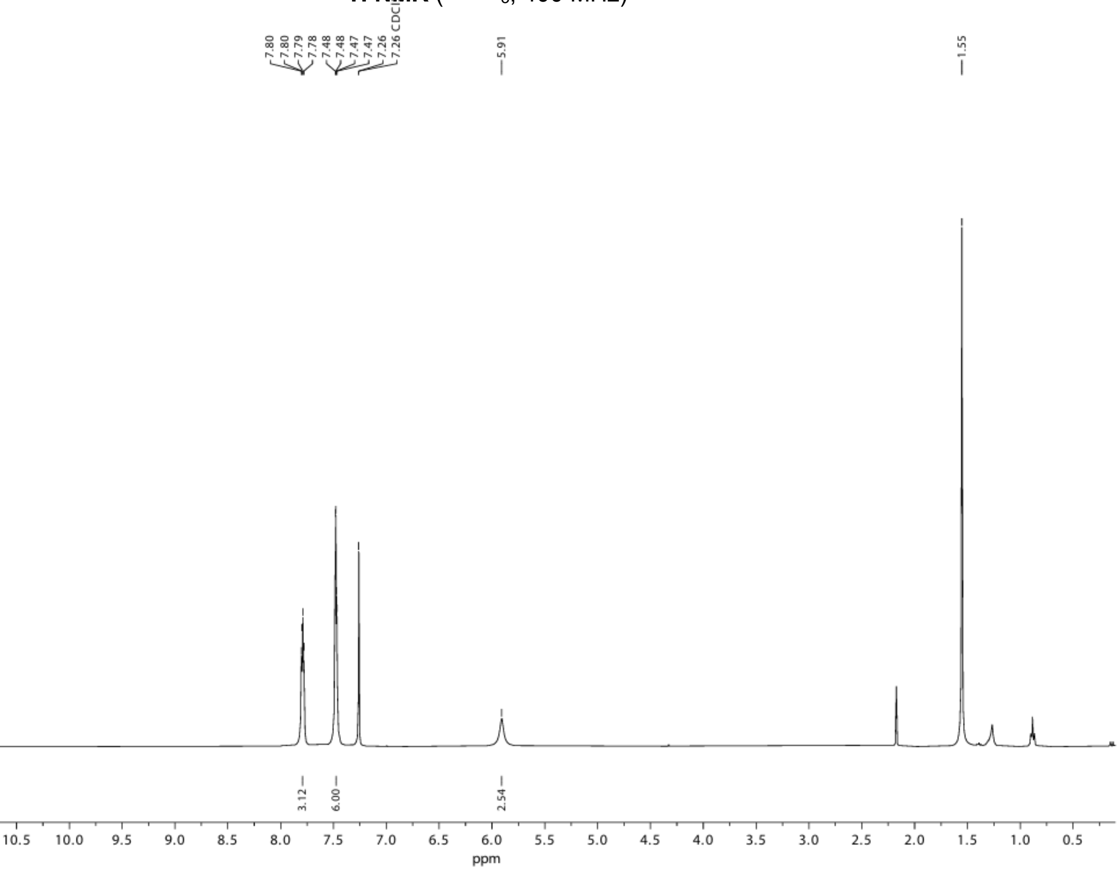

N-H-*B,B′,B′′*-tri(phenyl)borazine

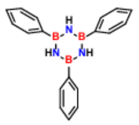

3b, 85%

^{11}B NMR ($CDCl_3$, 128 MHz)

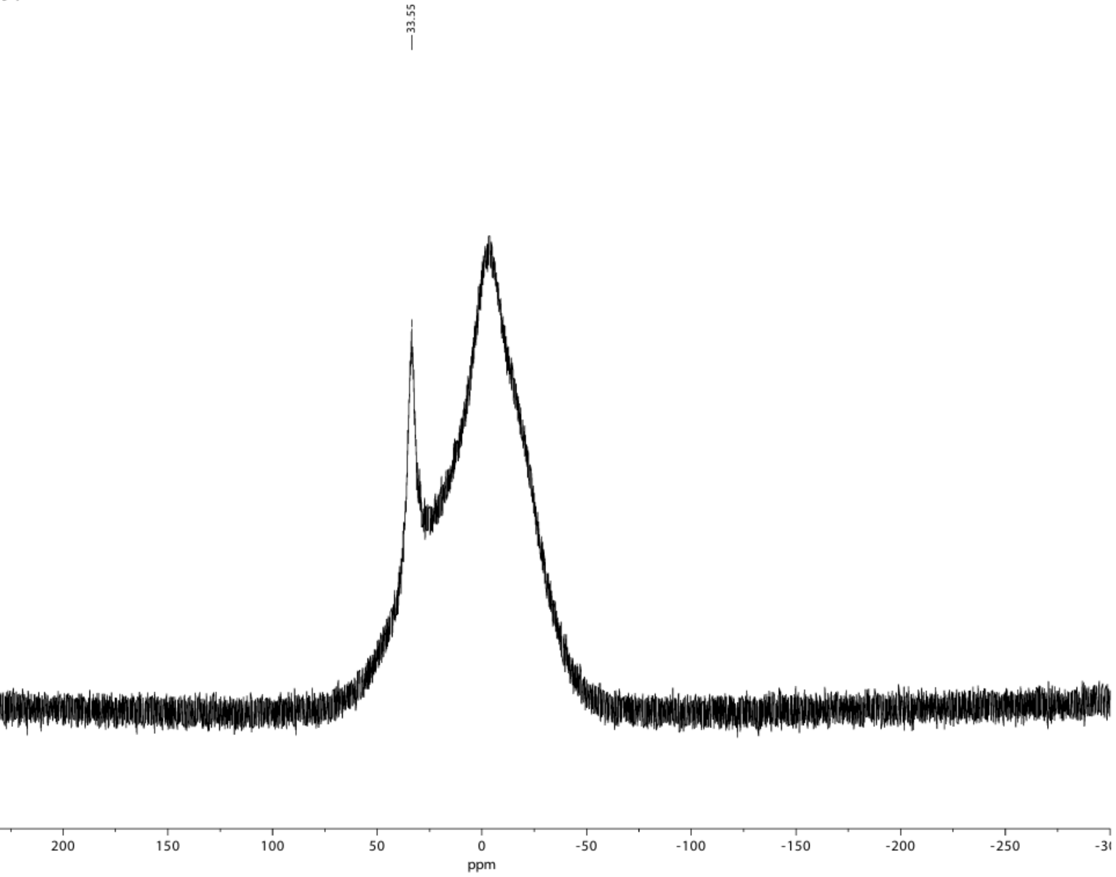

***N*-H-*B,B′,B″*-tri(phenyl)borazine**

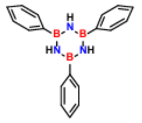

3b, 85%

^{13}C NMR ($CDCl_3$, 100.6 MHz)

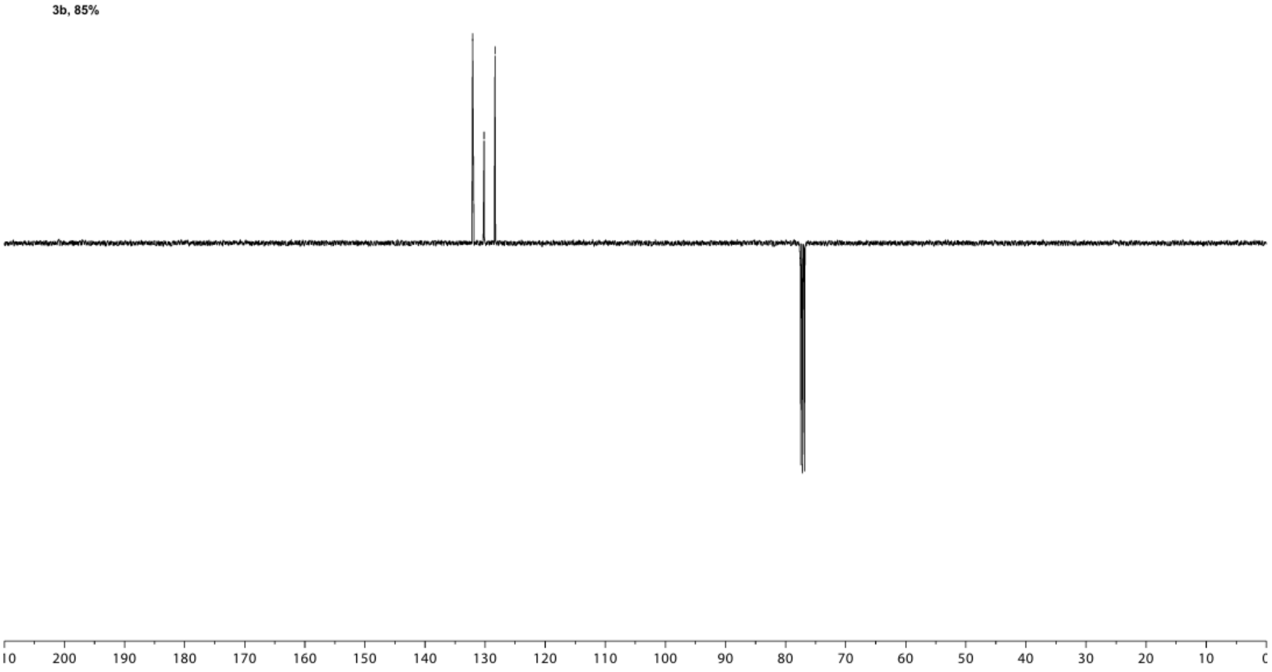

^{1}H NMR ($CDCl_3$, 400 MHz)

***N*-H-*B,B′,B″*-tri(4-fluorophenyl)borazine**

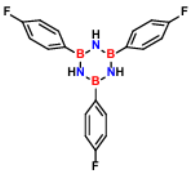

3c, 57%

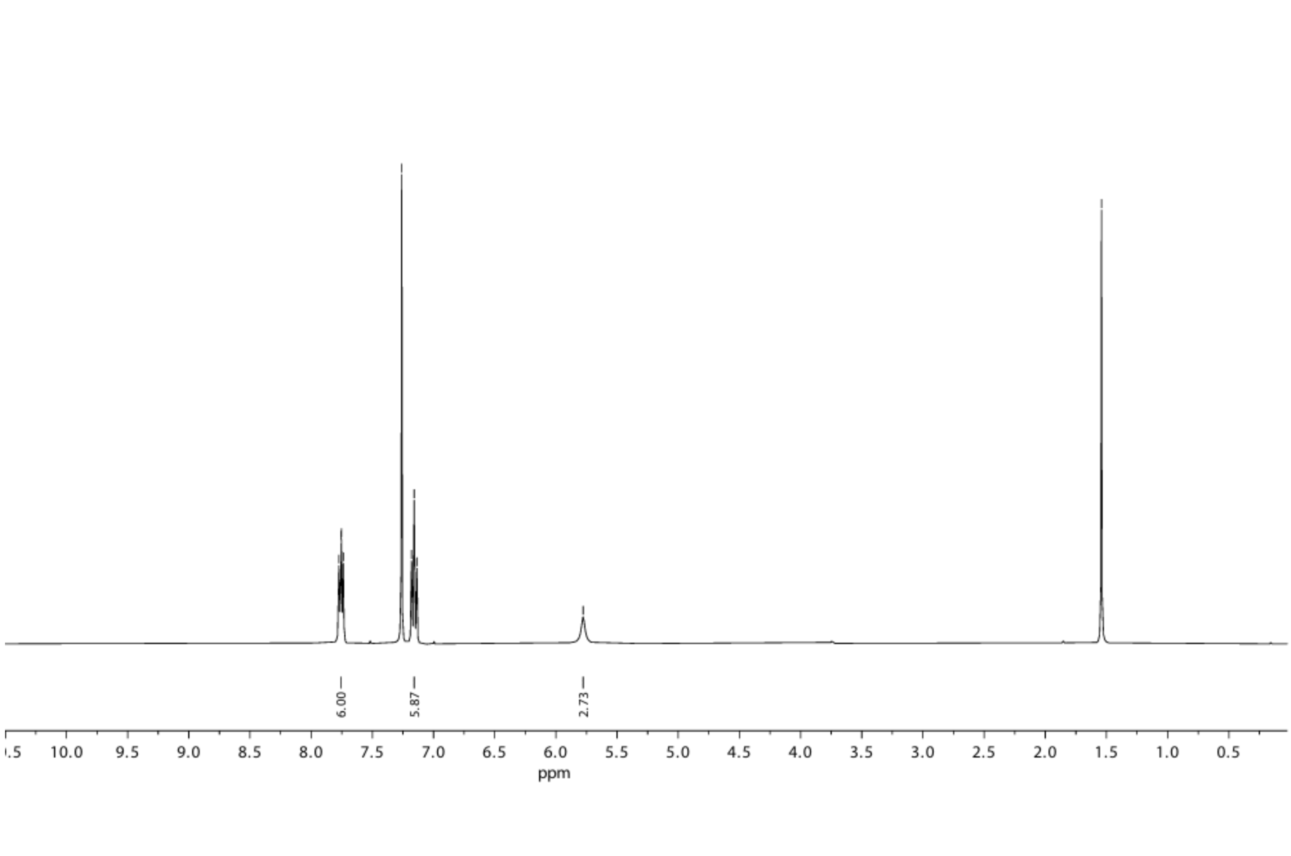

^{11}B NMR (CDCl$_3$, 128 MHz)

***N*-H-*B,B′,B′′*-tri(4-fluorophenyl)borazine**

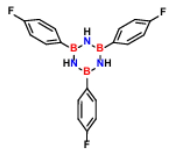

3c, 57%

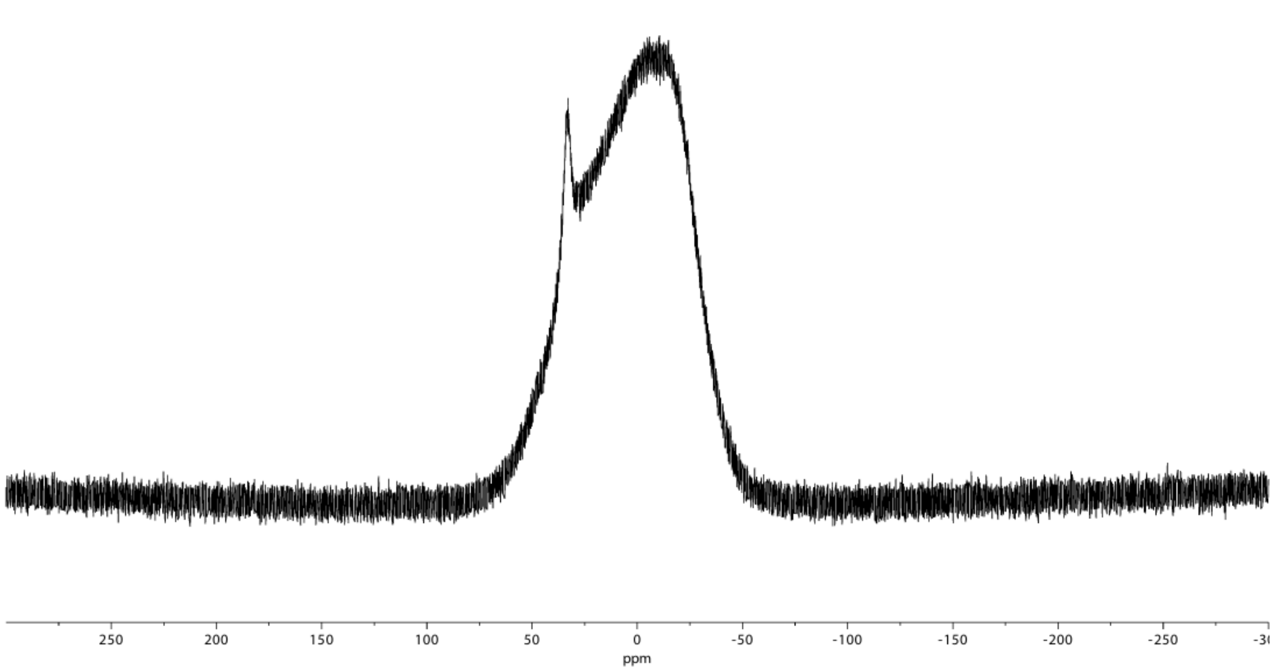

^{13}C NMR (CDCl$_3$, 100.6 MHz)

***N*-H-*B,B′,B′′*-tri(4-fluorophenyl)borazine**

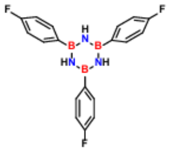

3c, 57%

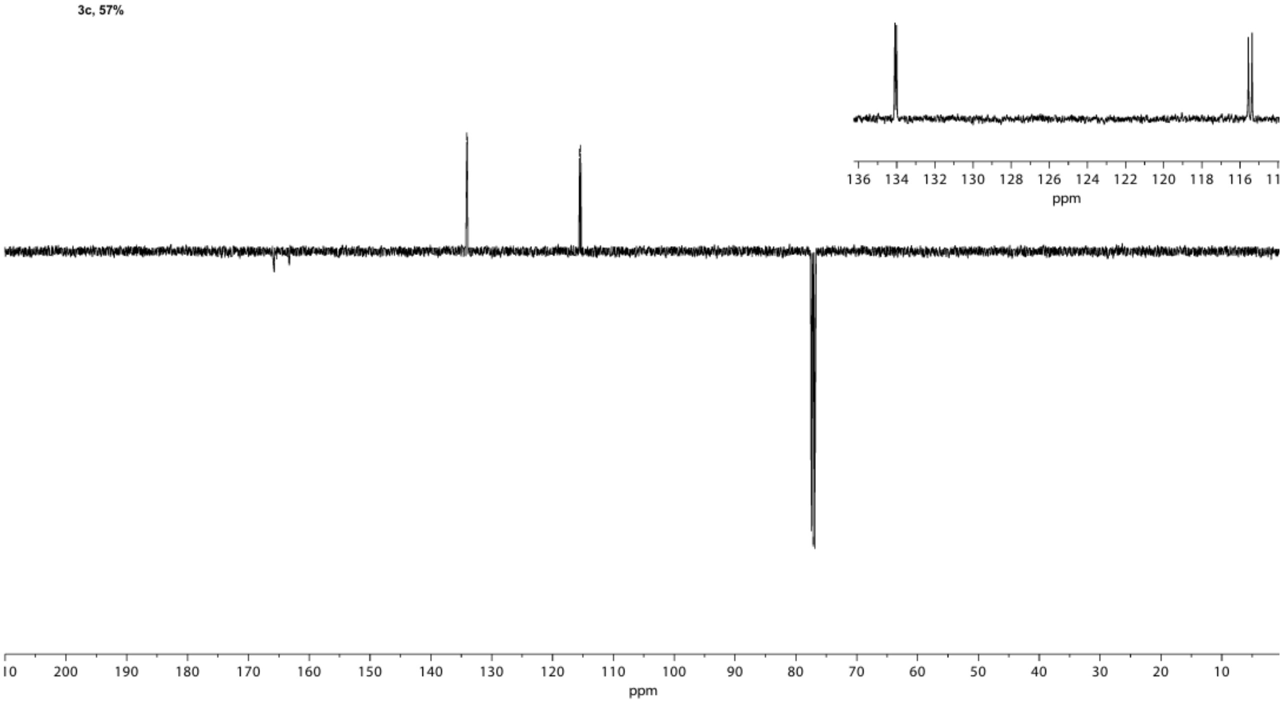

^{19}F NMR ($CDCl_3$, 376.4 MHz)

***N*-H-*B*,*B'*,*B''*-tri(4-fluorophenyl)borazine**

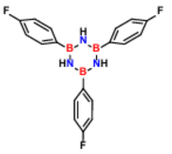

3c, 57%

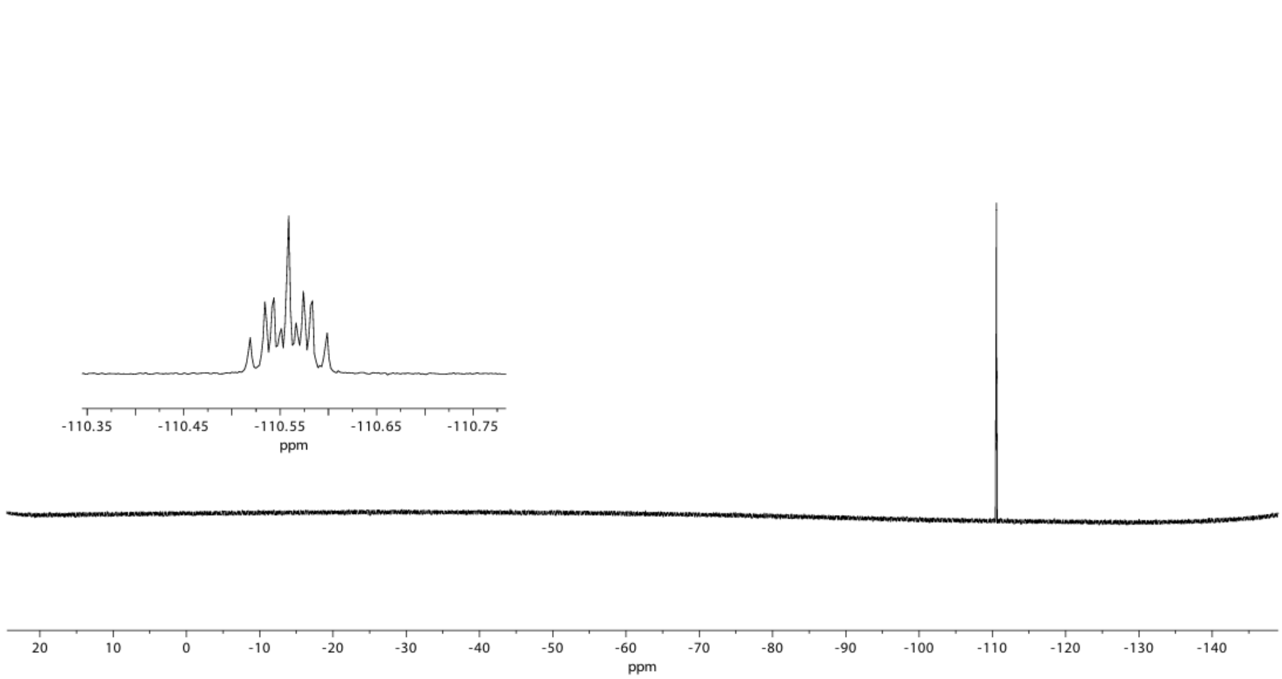

^{1}H NMR ($CDCl_3$, 400 MHz)

***N*-H-*B*,*B'*,*B''*-tri(4-chlorophenyl)borazine**

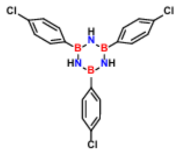

3d, 99%

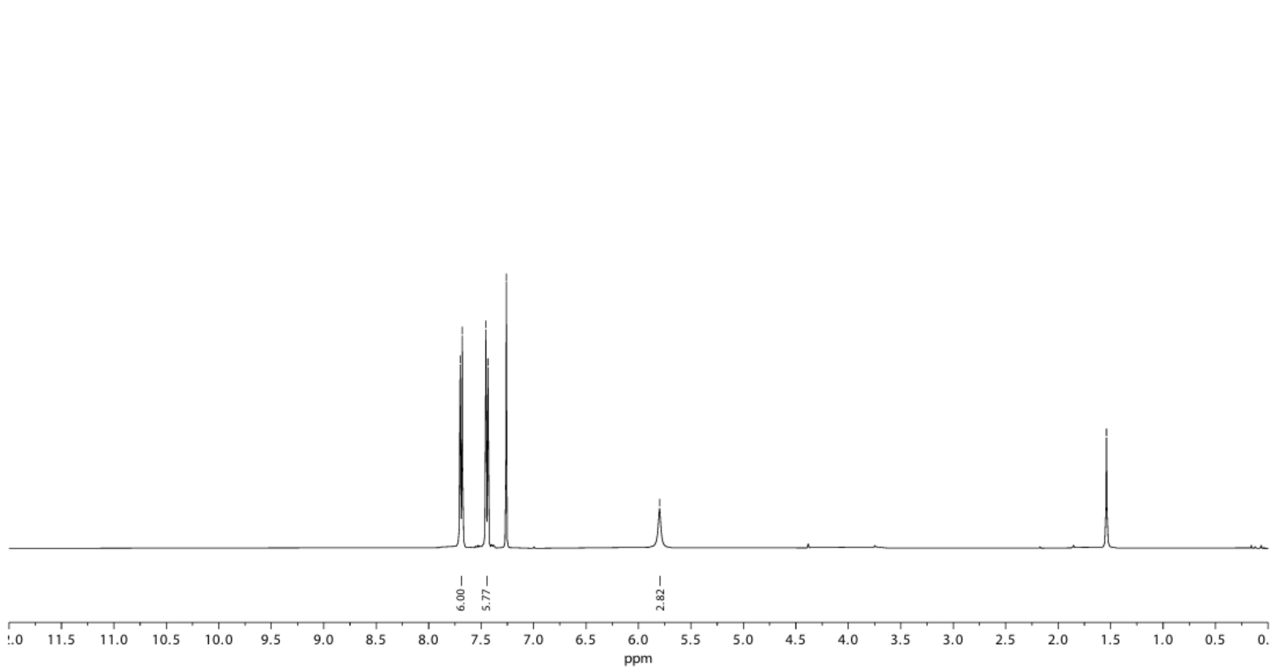

***N*-H-*B*,*B′*,*B″*-tri(4-chlorophenyl)borazine**

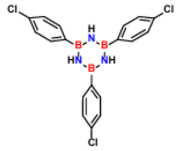

3d, 99%

^{11}B NMR (CDCl$_3$**,** 128 MHz)

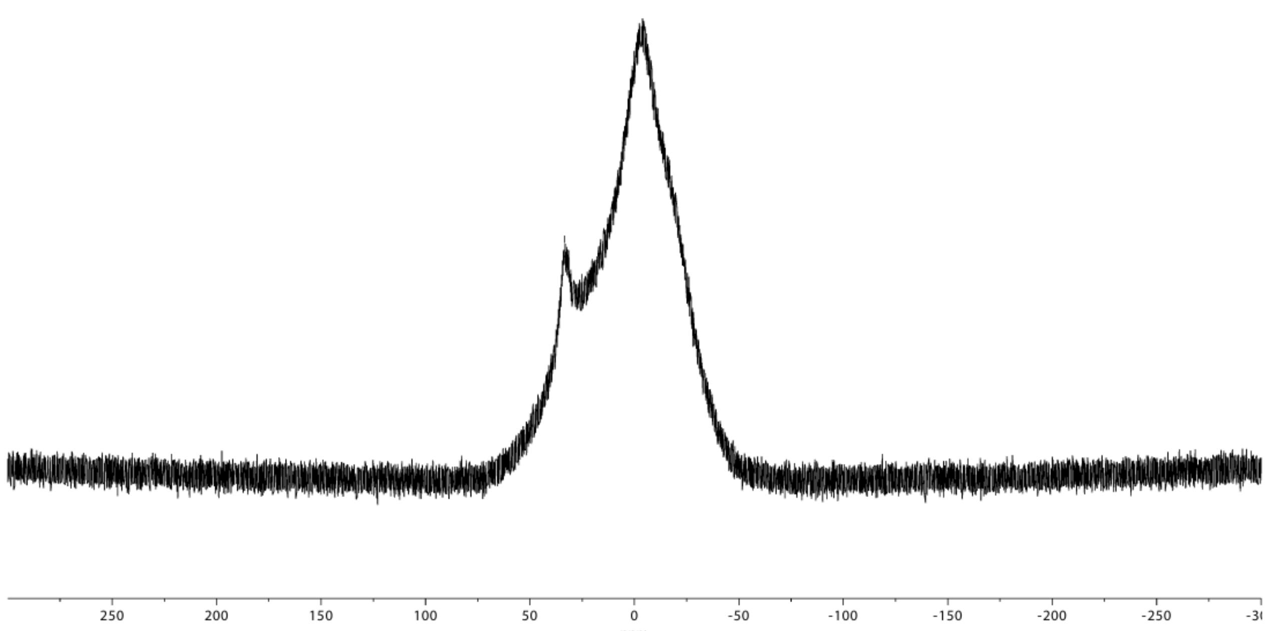

***N*-H-*B*,*B′*,*B″*-tri(4-chlorophenyl)borazine**

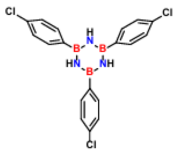

3d, 99%

^{13}C NMR (CDCl$_3$, 100.6 MHz)

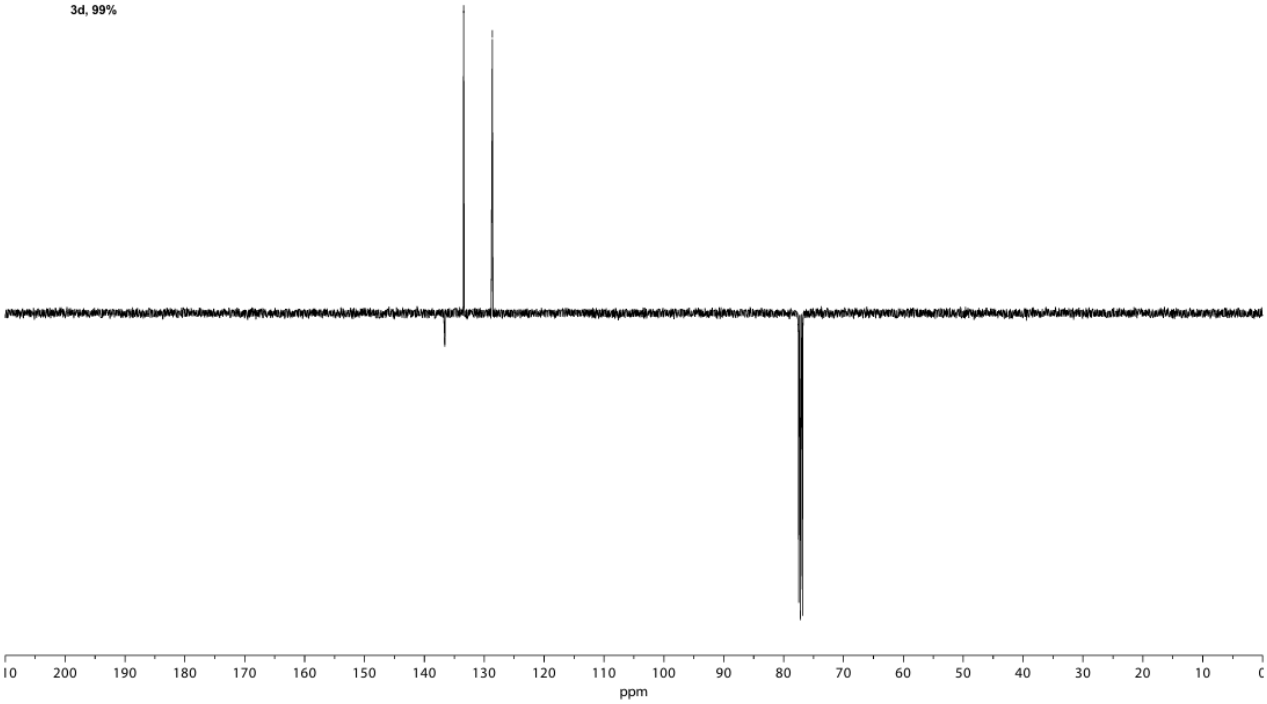

***N*-H-*B,B′,B′′*-tri(4-bromophenyl)borazine**

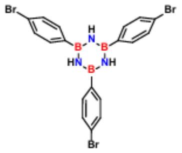

3e, 74%

^{1}H NMR ($CDCl_3$, 400 MHz)

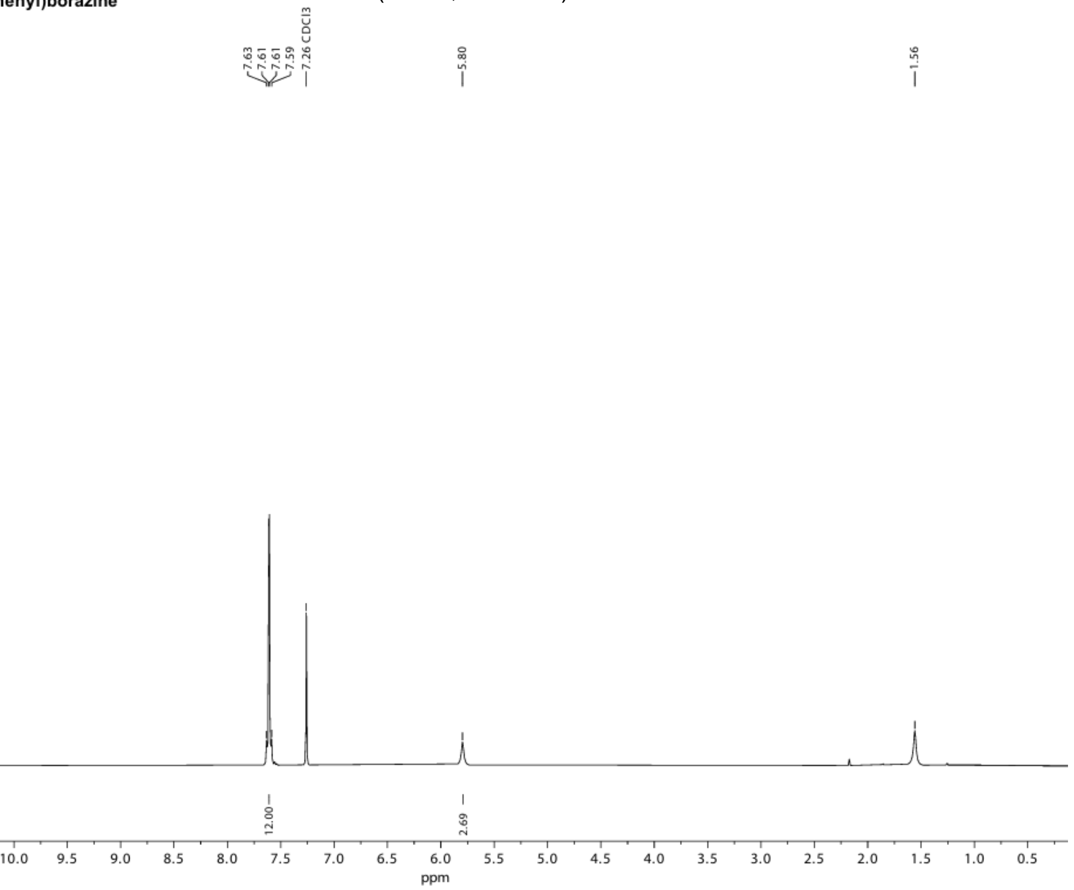

***N*-H-*B,B′,B′′*-tri(4-bromophenyl)borazine**

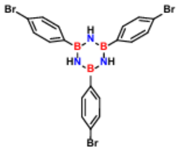

3e, 74%

^{11}B NMR ($CDCl_3$**,** 128 MHz)

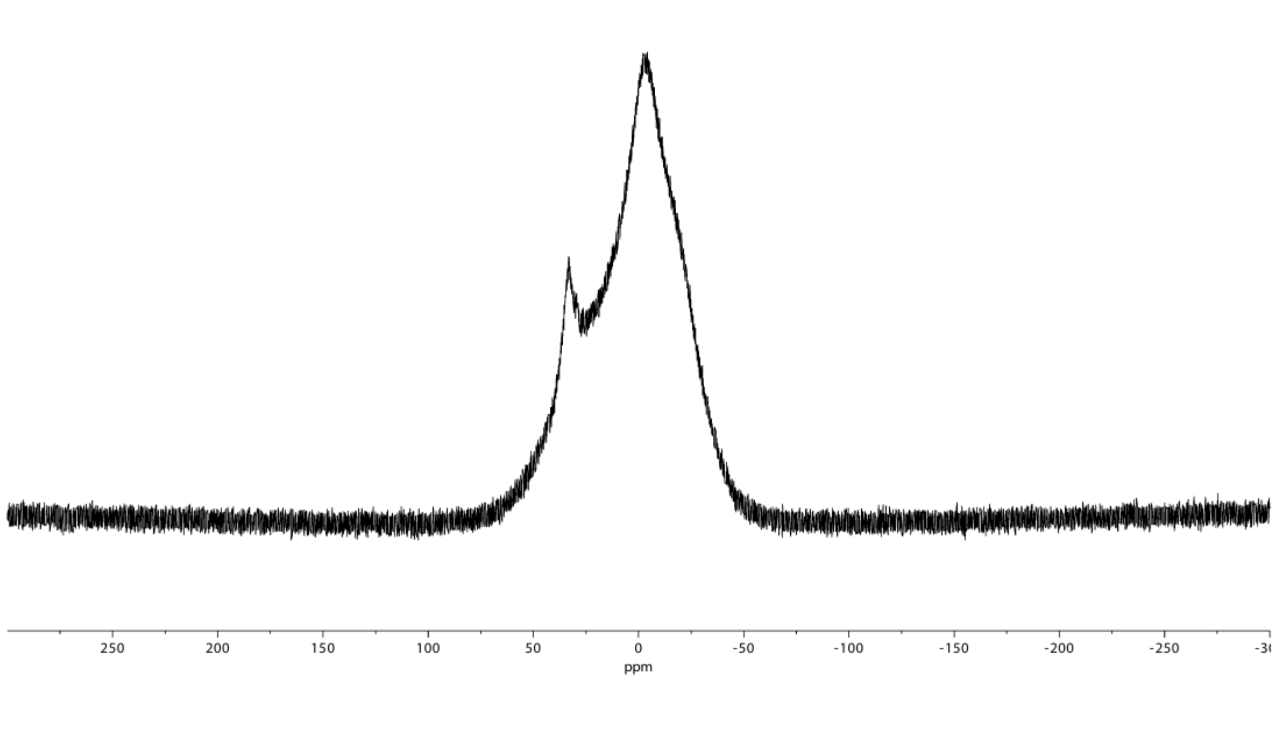

***N*-H-*B,B′,B′′*-tri(4-bromophenyl)borazine**

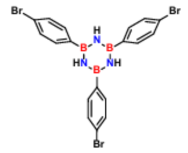

3e, 74%

^{13}C NMR ($CDCl_3$, 100.6 MHz)

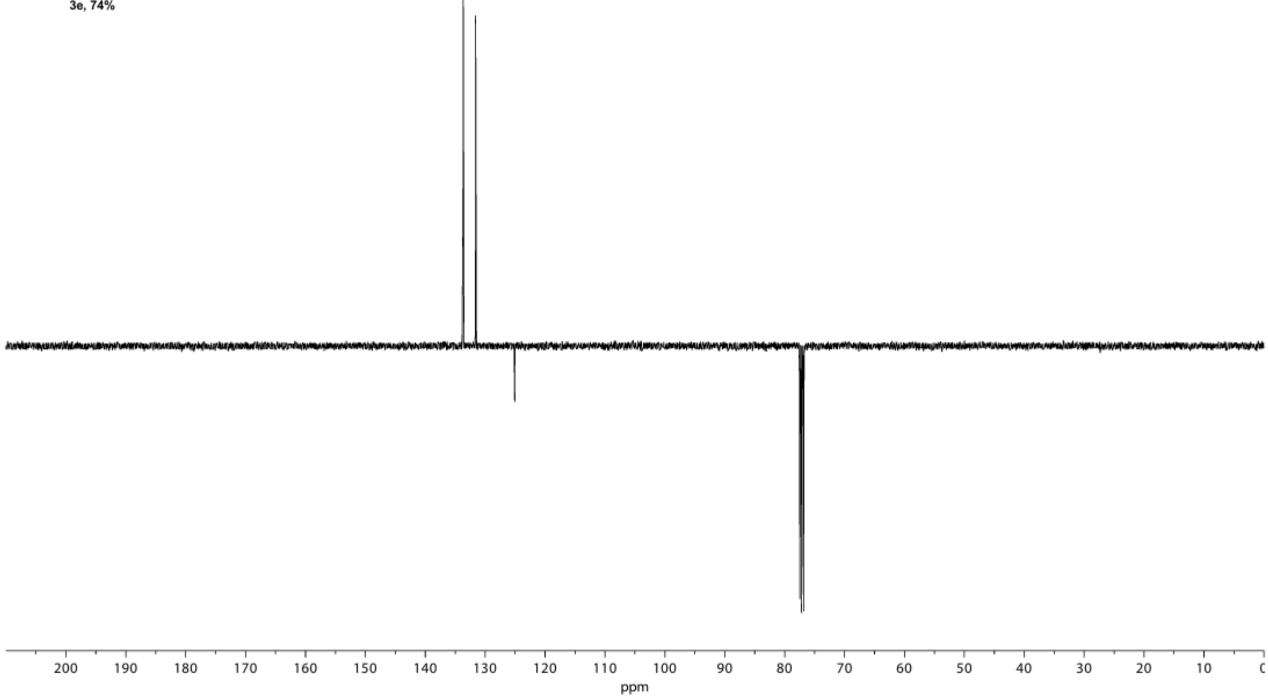

^{1}H NMR ($CDCl_3$, 400 MHz)

2-(4-iodophenyl)-1,3,2-dioxaborolane

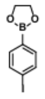

1f, 99%

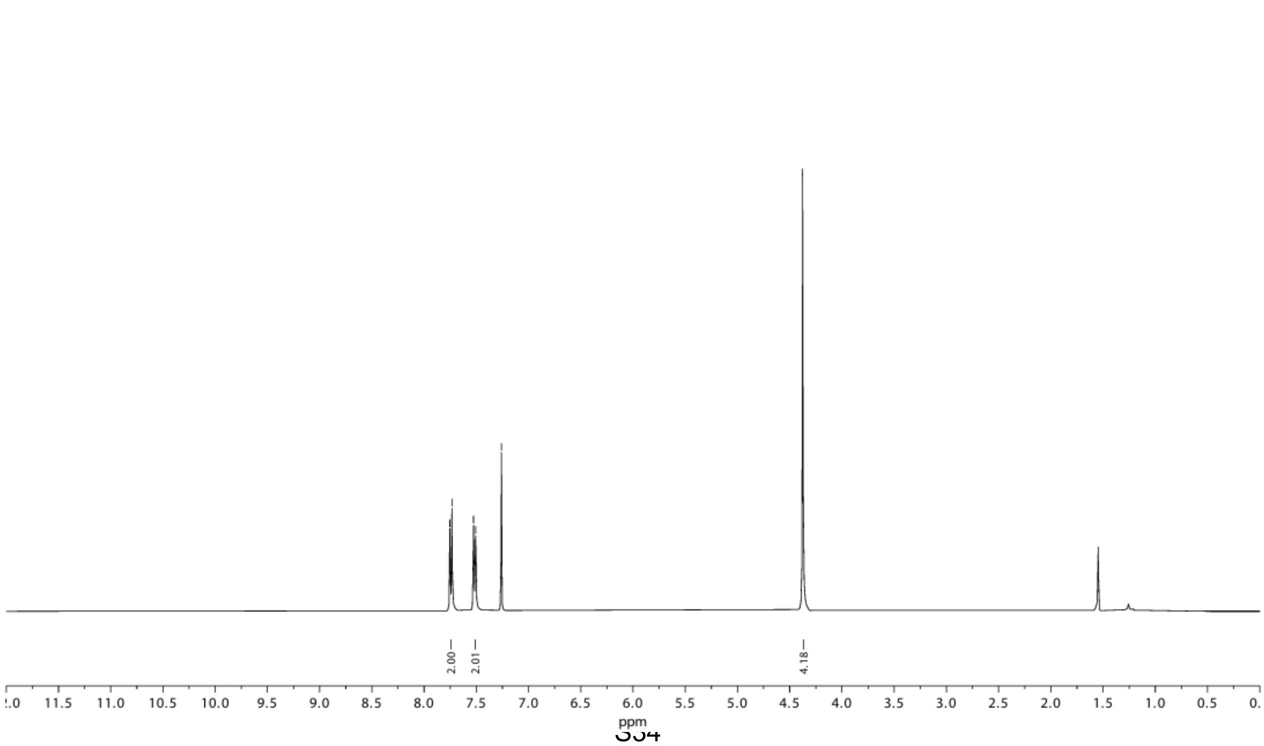

2-(4-iodophenyl)-1,3,2-dioxaborolane

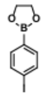

1f, 99%

^{11}B NMR (CDCl$_3$, 128 MHz)

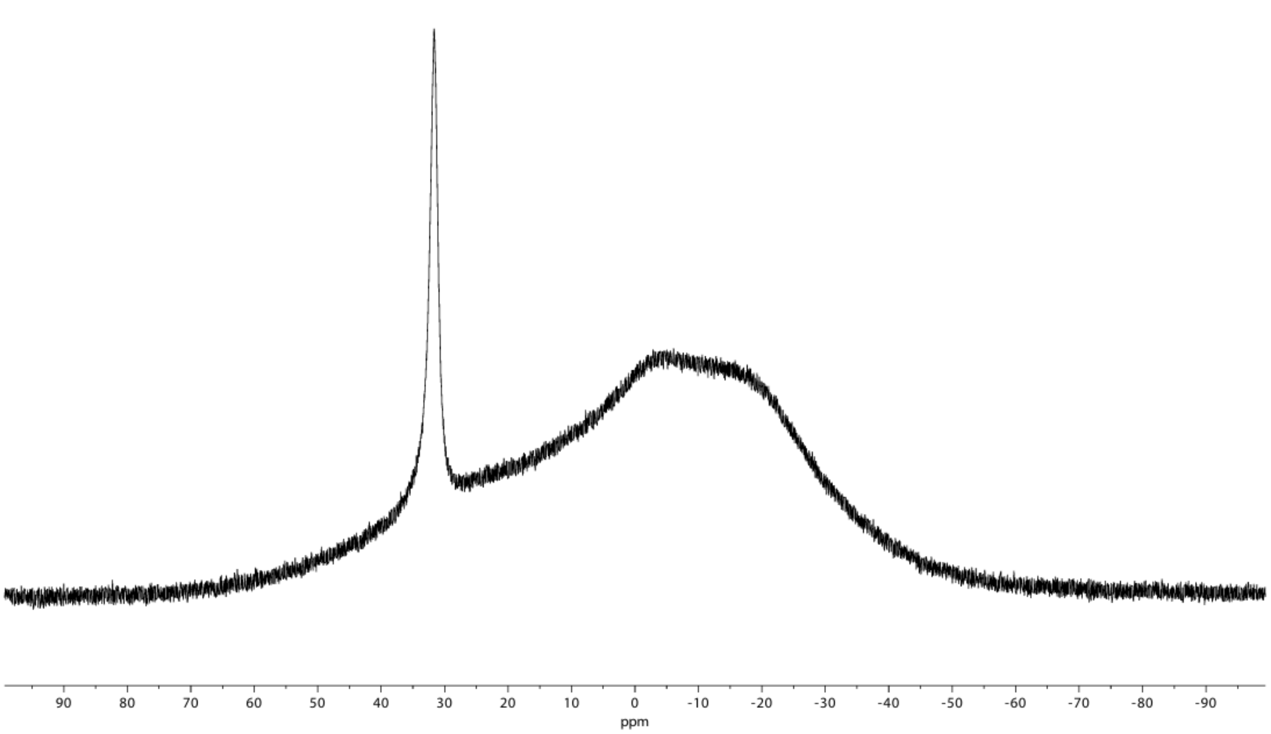

2-(4-iodophenyl)-1,3,2-dioxaborolane

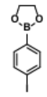

1f, 99%

^{13}C NMR (CDCl$_3$, 100.6 MHz)

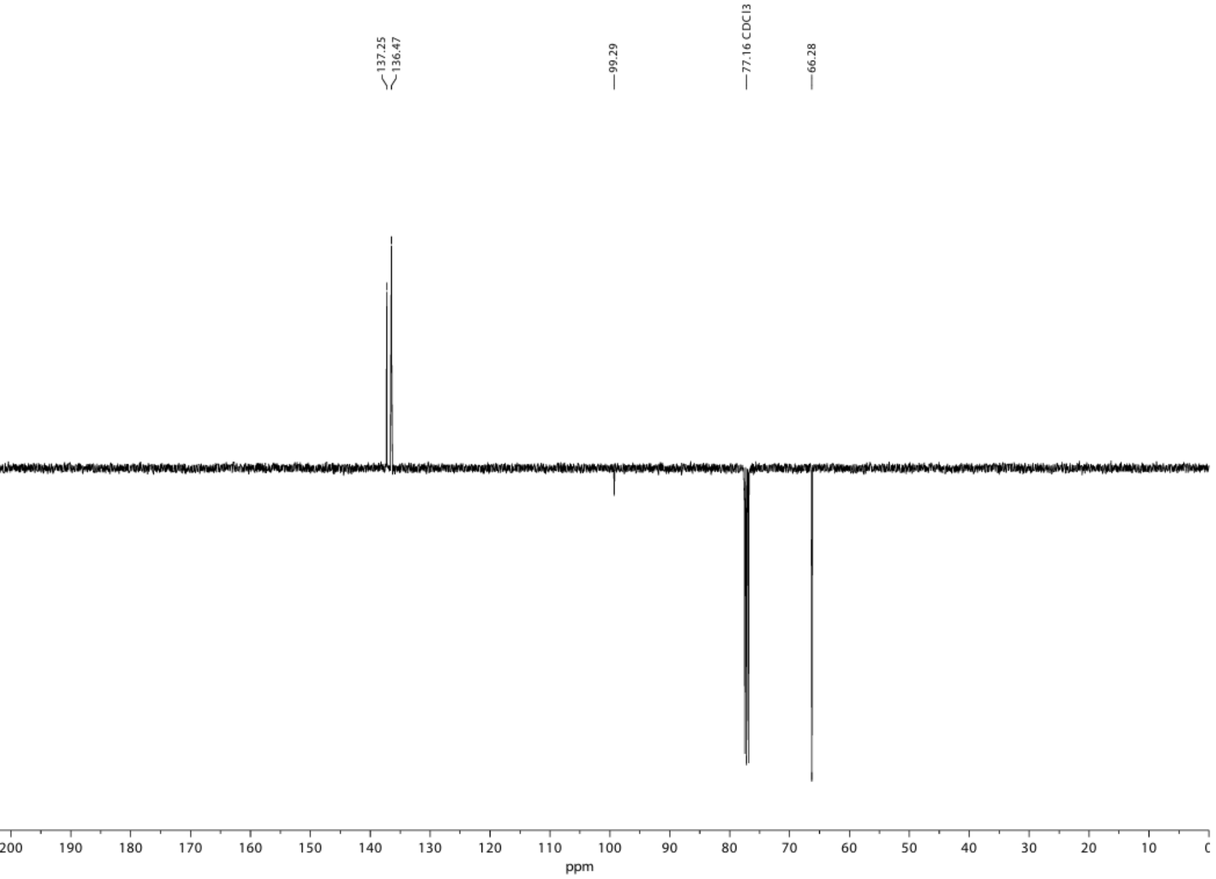

N-H-B,B′,B″-tri(4-iodophenyl)borazine

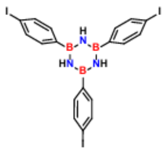

3f, 70%

^{1}H NMR (CDCl$_3$, 400 MHz)

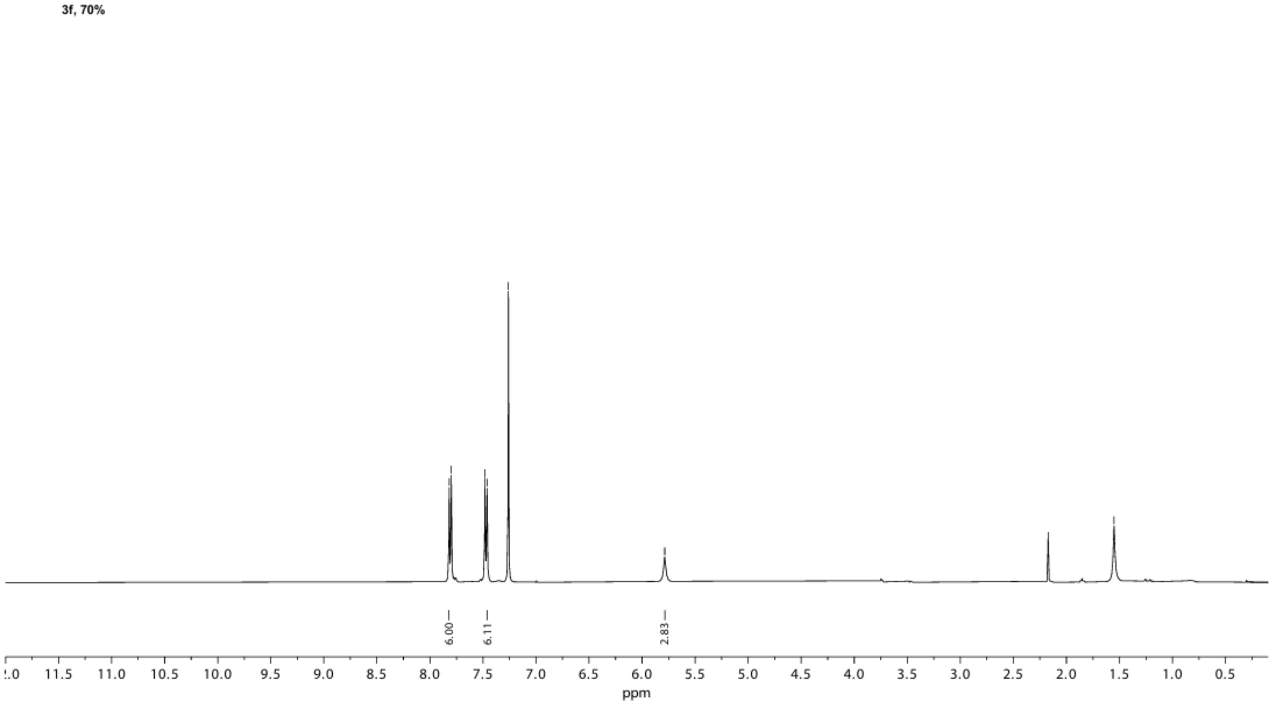

N-H-B,B′,B″-tri(4-iodophenyl)borazine

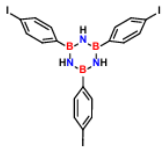

3f, 70%

^{11}B NMR (CDCl$_3$, 128 MHz)

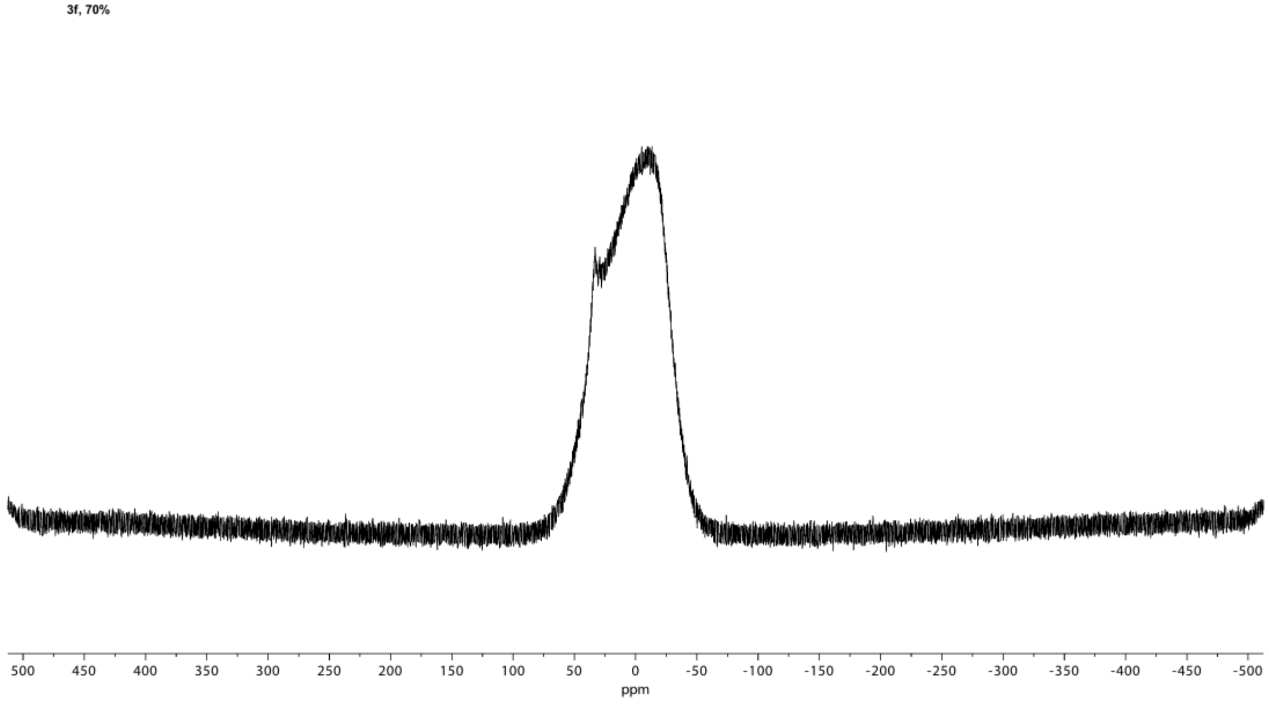

***N*-H-*B*,*B′*,*B″*-tri(4-iodophenyl)borazine**

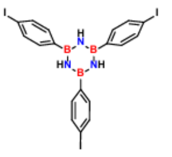

3f, 70%

^{13}C NMR (CDCl$_3$, 100.6 MHz)

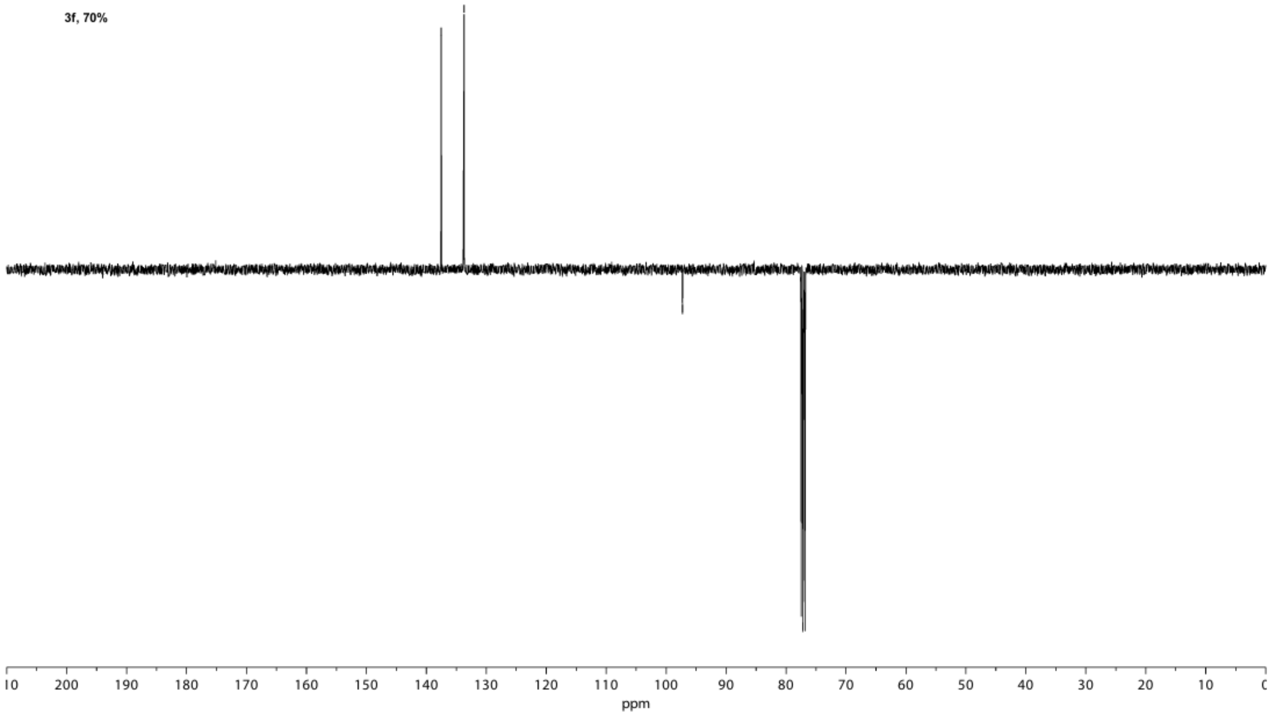

***N*-H-*B*,*B′*,*B″*-tri(4- diphenylaminophenyl)borazine**

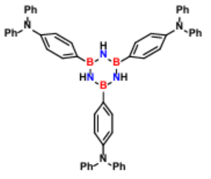

3g, 84%

^{1}H NMR (CDCl$_3$, 400 MHz)

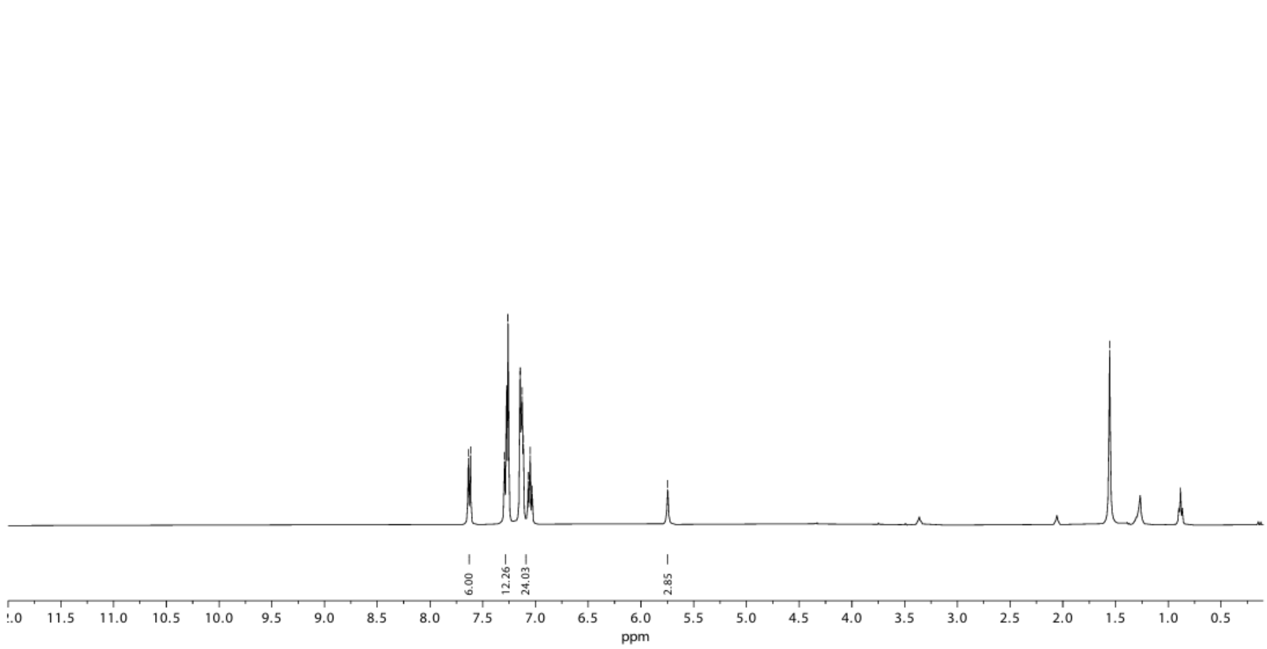

***N*-H-*B*,*B′*,*B′′*-tri(4- diphenylaminophenyl)borazine**

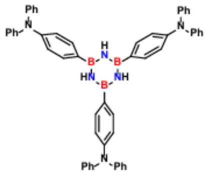

3g, 84%

^{13}C NMR (CDCl$_3$, 100.6 MHz)

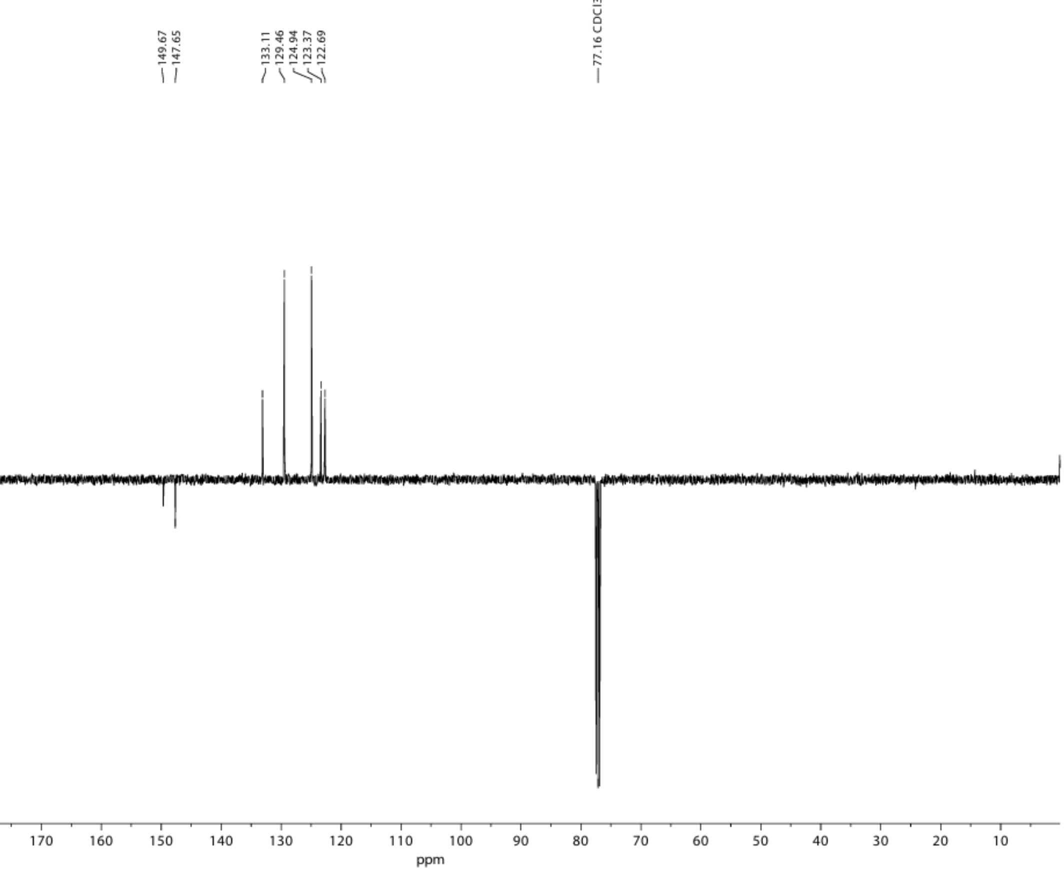

***N*-H-*B*,*B′*,*B′′*-tri(4-methoxyphenyl)borazine**

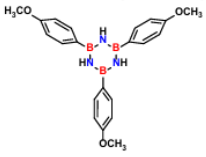

3h, 99%

^{1}H NMR (CDCl$_3$, 400 MHz)

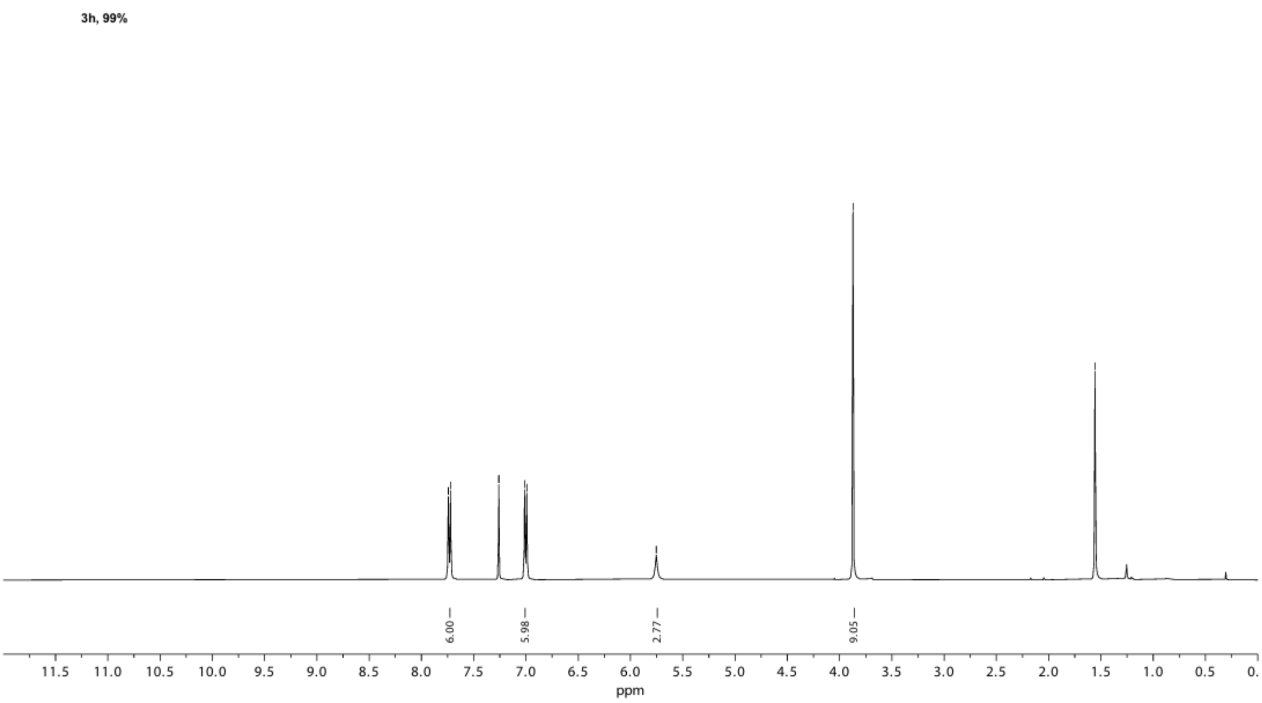

***N*-H-*B,B′,B″*-tri(4-methoxyphenyl)borazine**

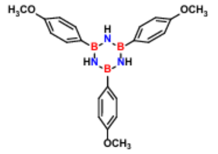

3h, 99%

^{11}B NMR (CDCl$_3$, 128 MHz)

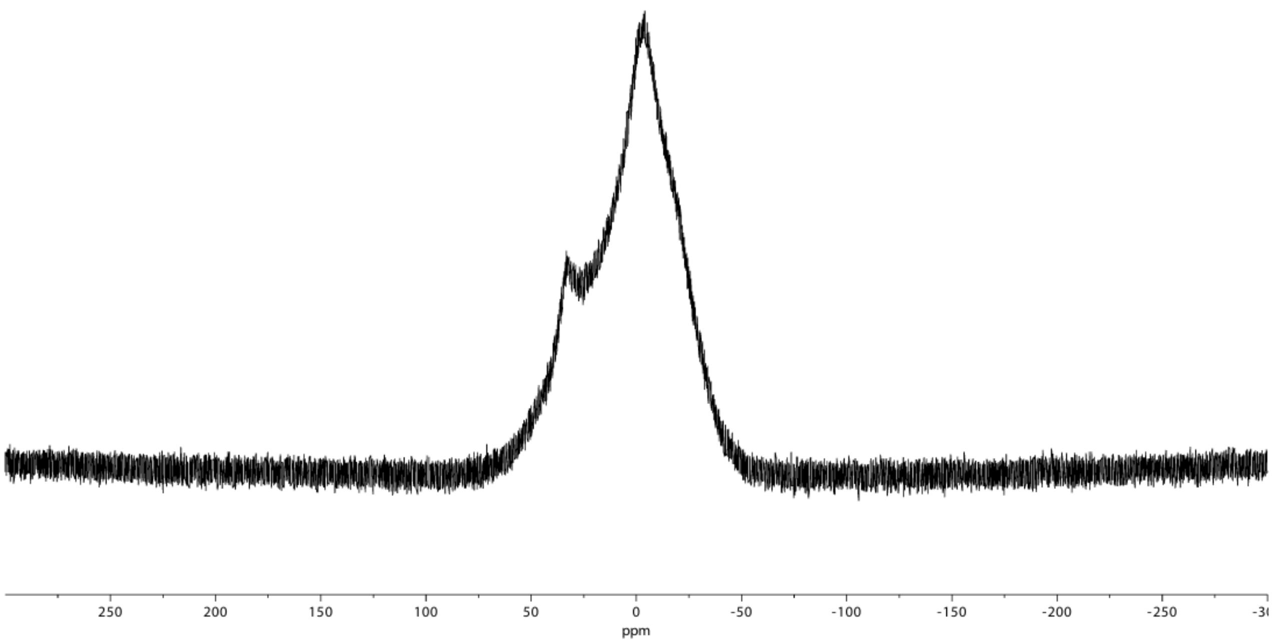

***N*-H-*B,B′,B″*-tri(4-methoxyphenyl)borazine**

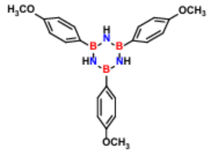

3h, 99%

^{13}C NMR (CDCl$_3$, 100.6 MHz)

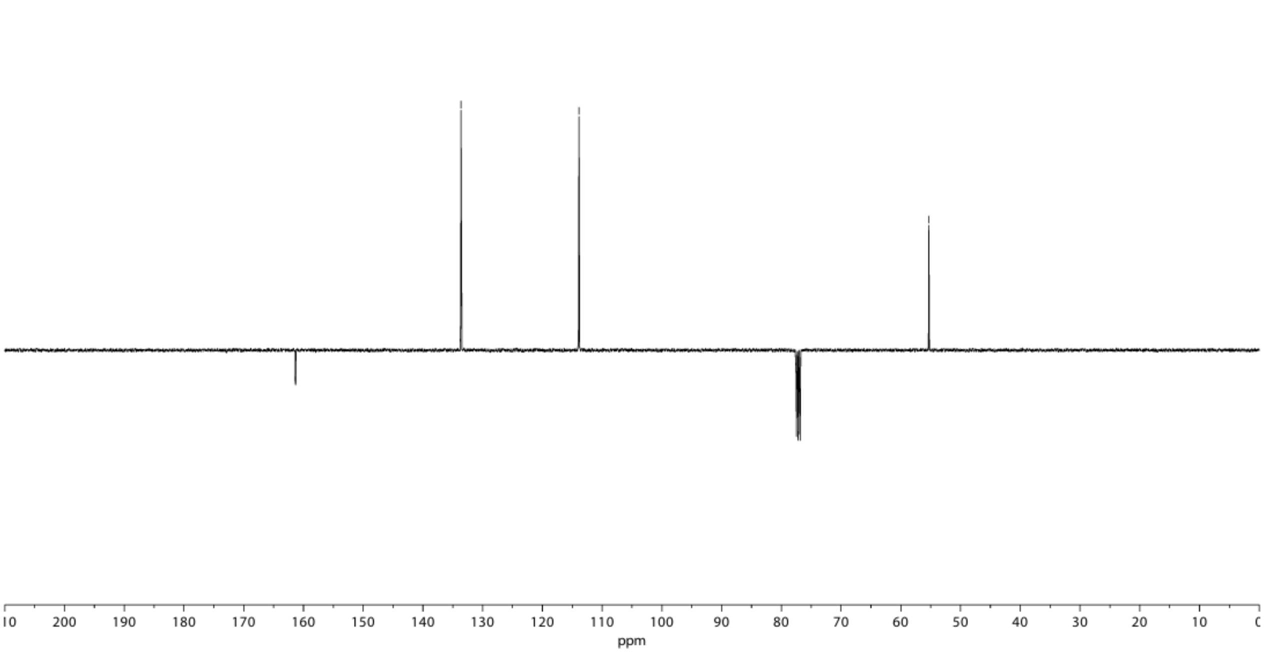

***N*-H-*B*,*B′*,*B″*-tri(4-trimethylsilyloxyphenyl)borazine**

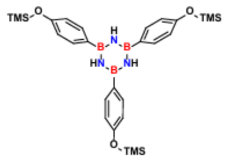

3i, 26%

^{1}H NMR ($CDCl_3$, 400 MHz)

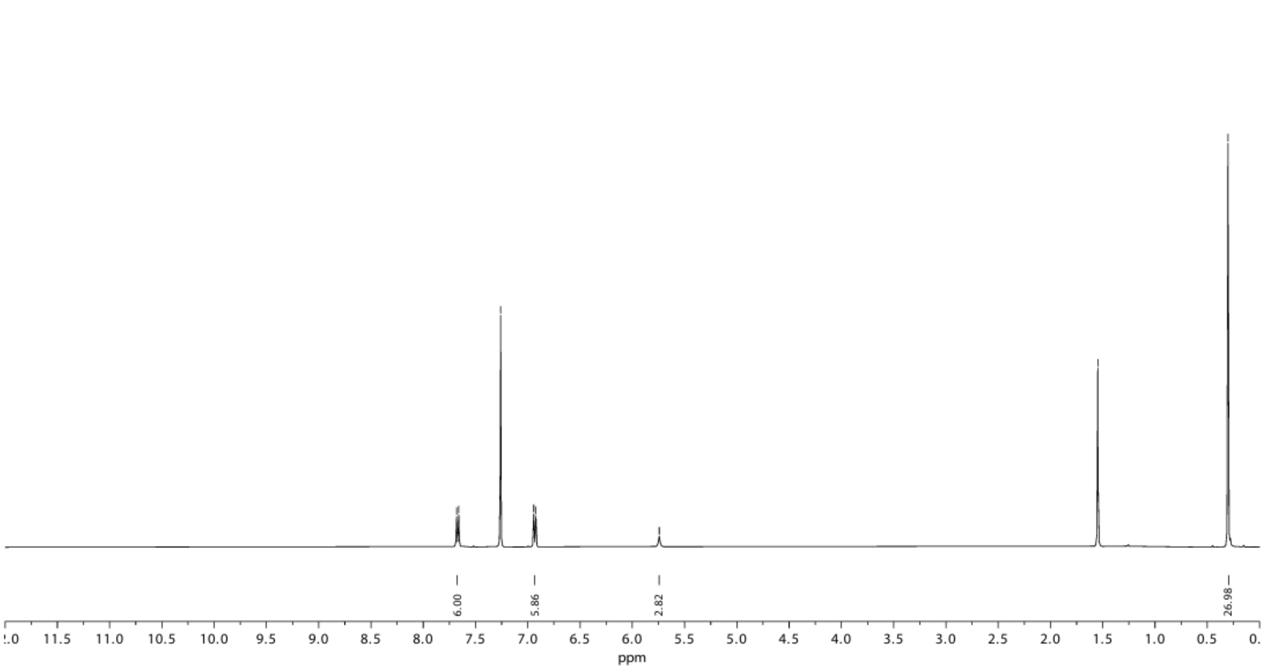

***N*-H-*B*,*B′*,*B″*-tri(4-trimethylsilyloxyphenyl)borazine**

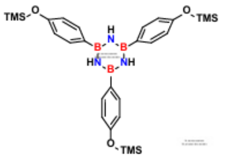

3i, 26%

^{11}B NMR ($CDCl_3$, 128 MHz)

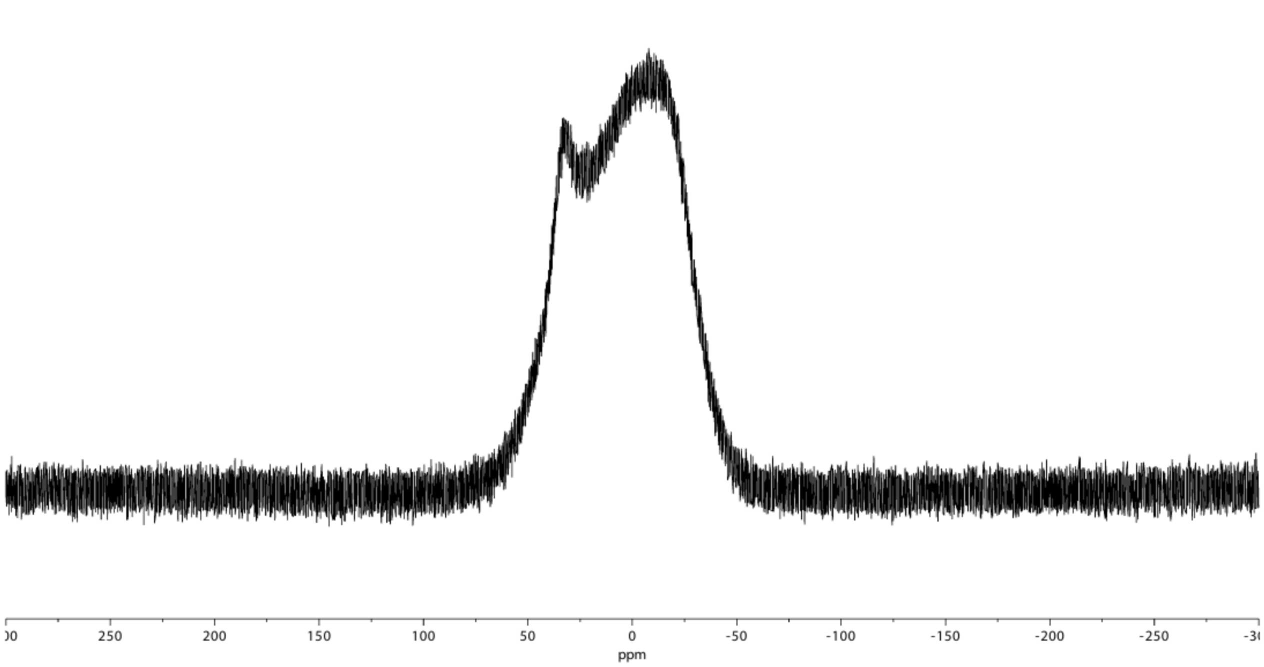

***N*-H-*B,B′,B″*-tri(4-trimethylsilyloxyphenyl)borazine**

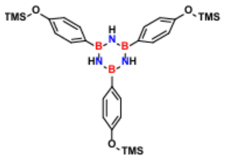

3i, 26%

^{13}C NMR ($CDCl_3$, 100.6 MHz)

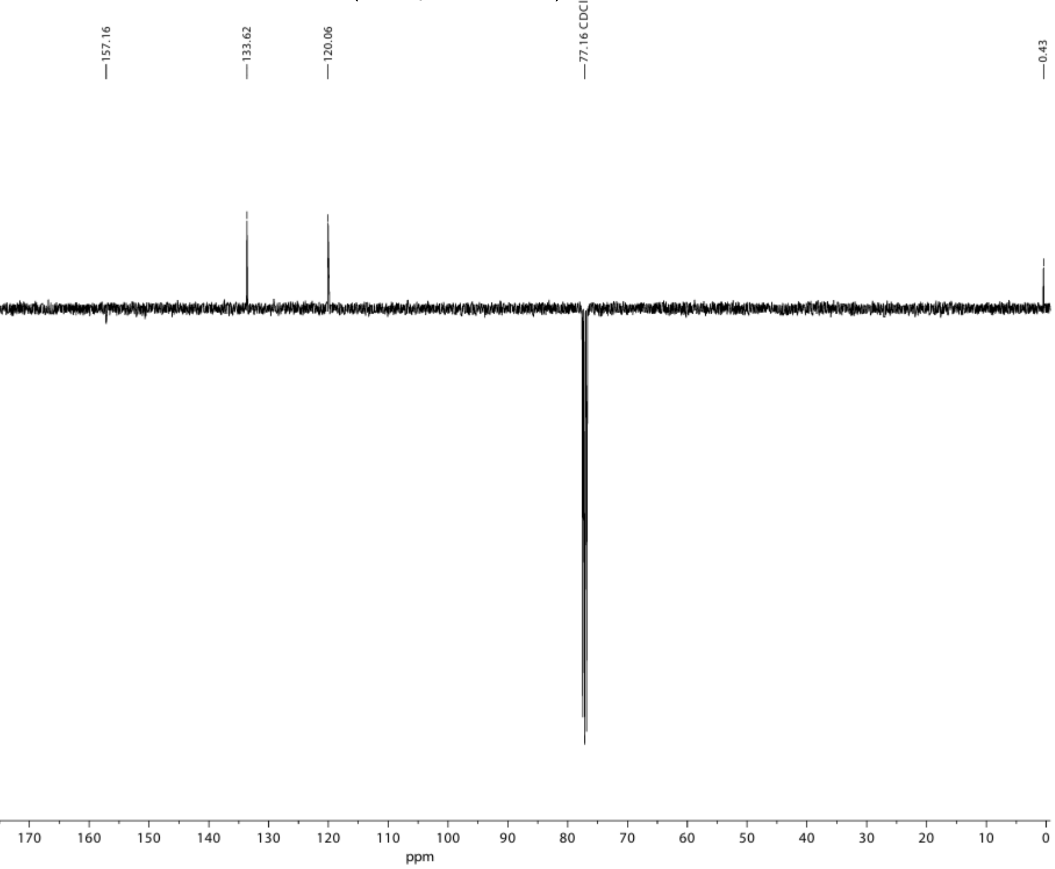

***N*-H-*B,B′,B″*-tri(4-trimethylsilylphenyl)borazine**

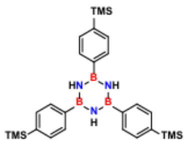

3j, 49%

^{1}H NMR ($CDCl_3$, 400 MHz)

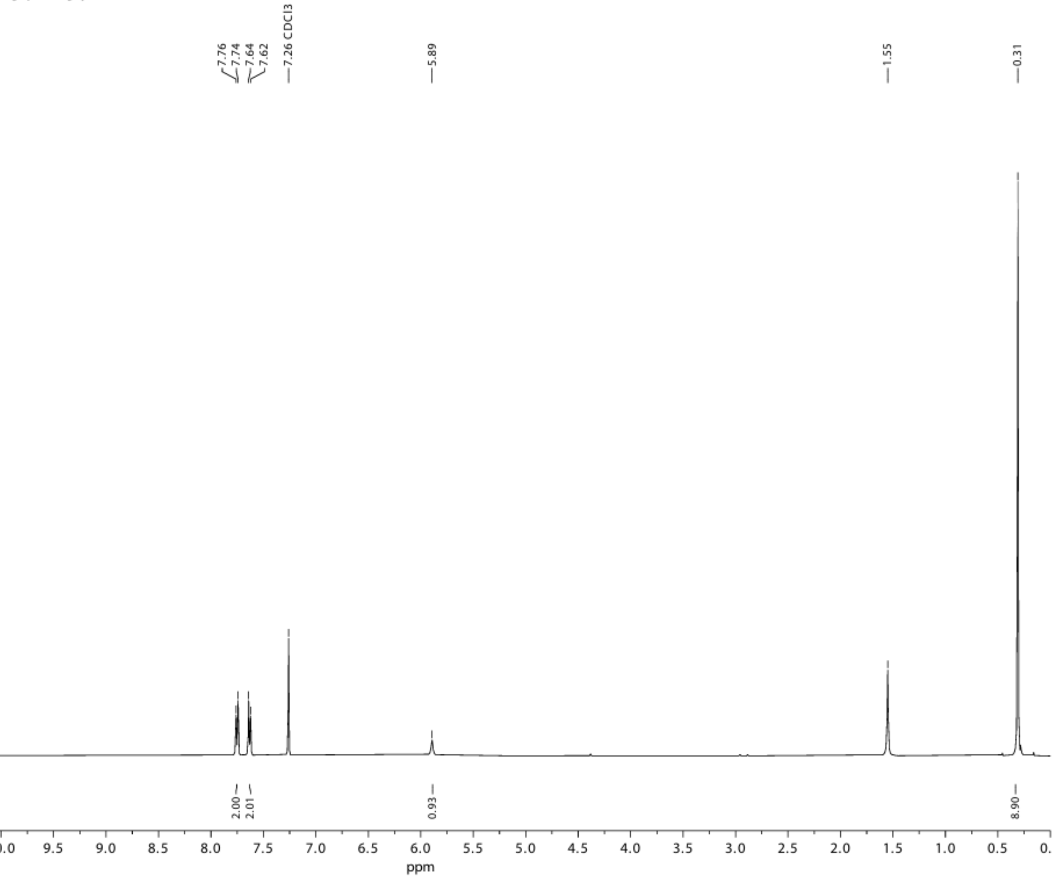

***N*-H-*B,B′,B″*-tri(4-trimethylsilylphenyl)borazine**

^{11}B NMR (CDCl$_3$**,** 128 MHz)

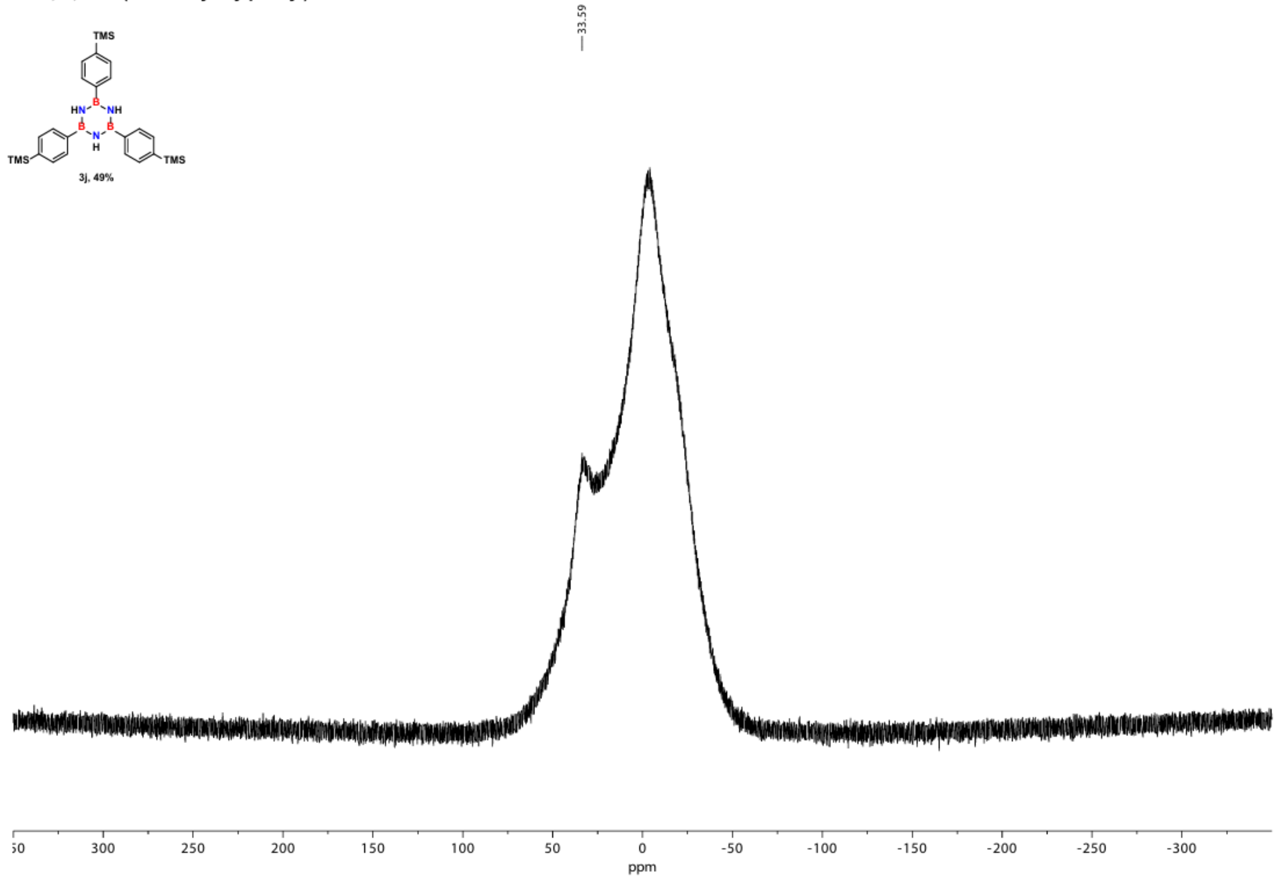

***N*-H-*B,B′,B″*-tri(4-trimethylsilylphenyl)borazine**

^{13}C NMR (CDCl$_3$, 100.6 MHz)

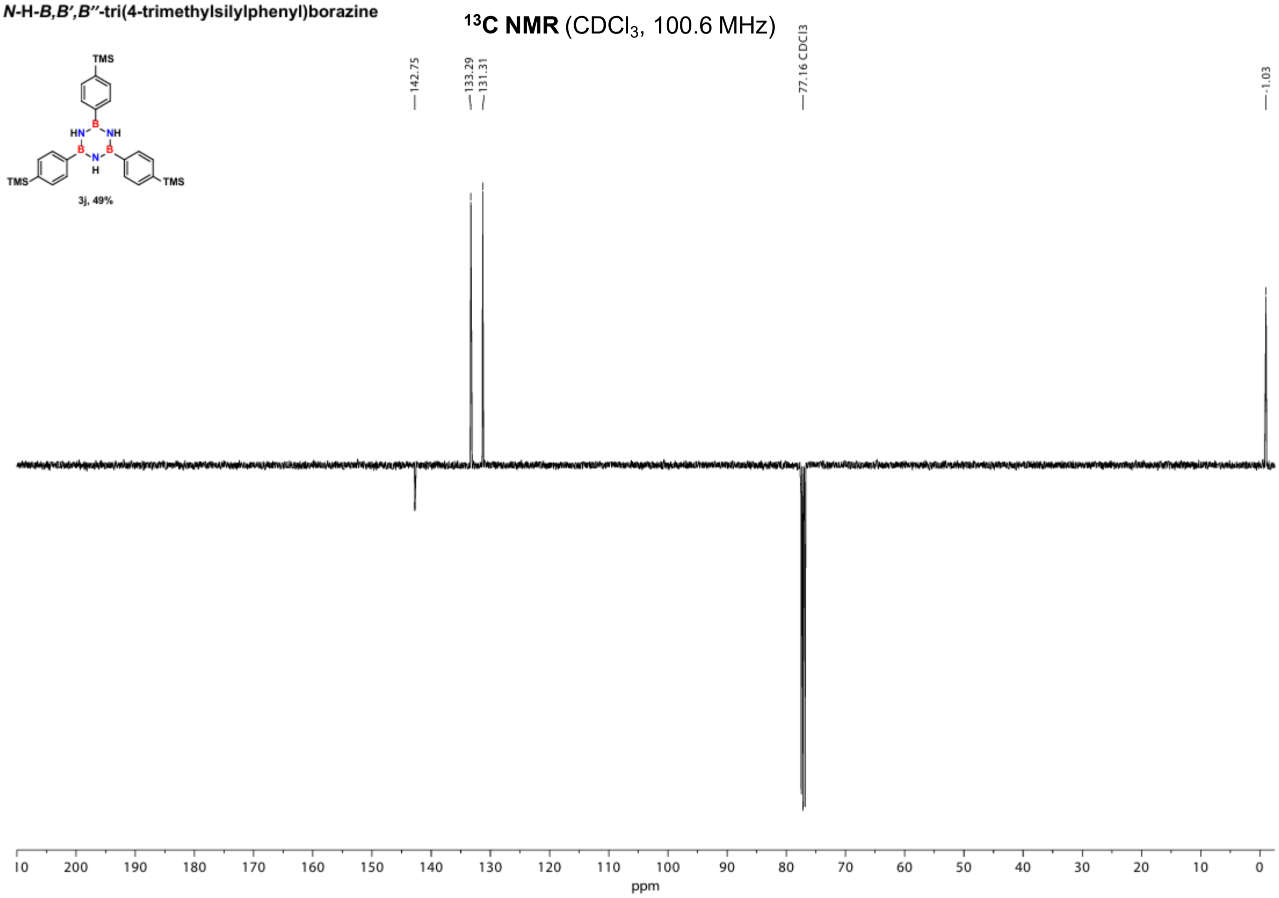

2-(4-tert-butylphenyl)-1,3,2-dioxaborolane

^{1}H NMR ($CDCl_3$, 400 MHz)

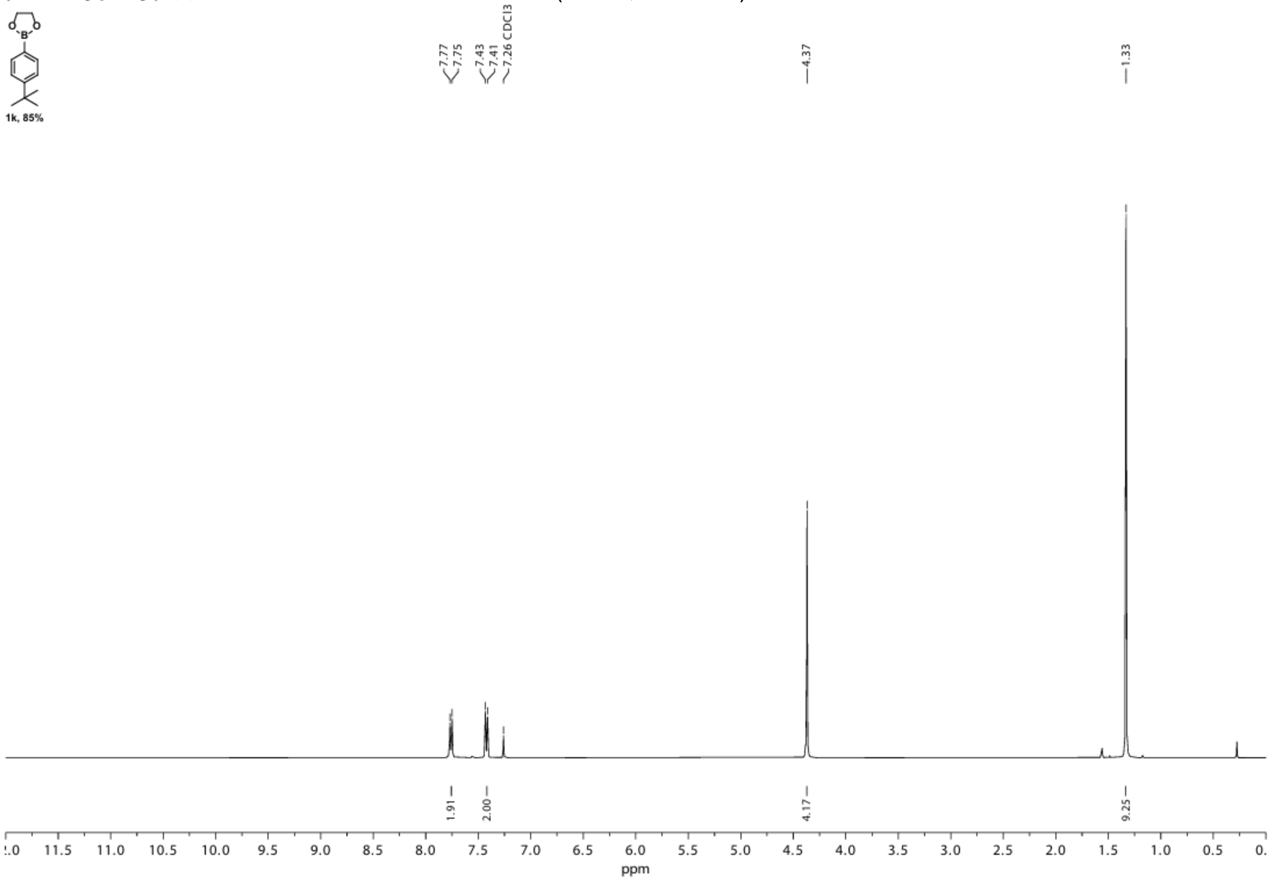

2-(4-tert-butylphenyl)-1,3,2-dioxaborolane

^{11}B NMR ($CDCl_3$, 128 MHz)

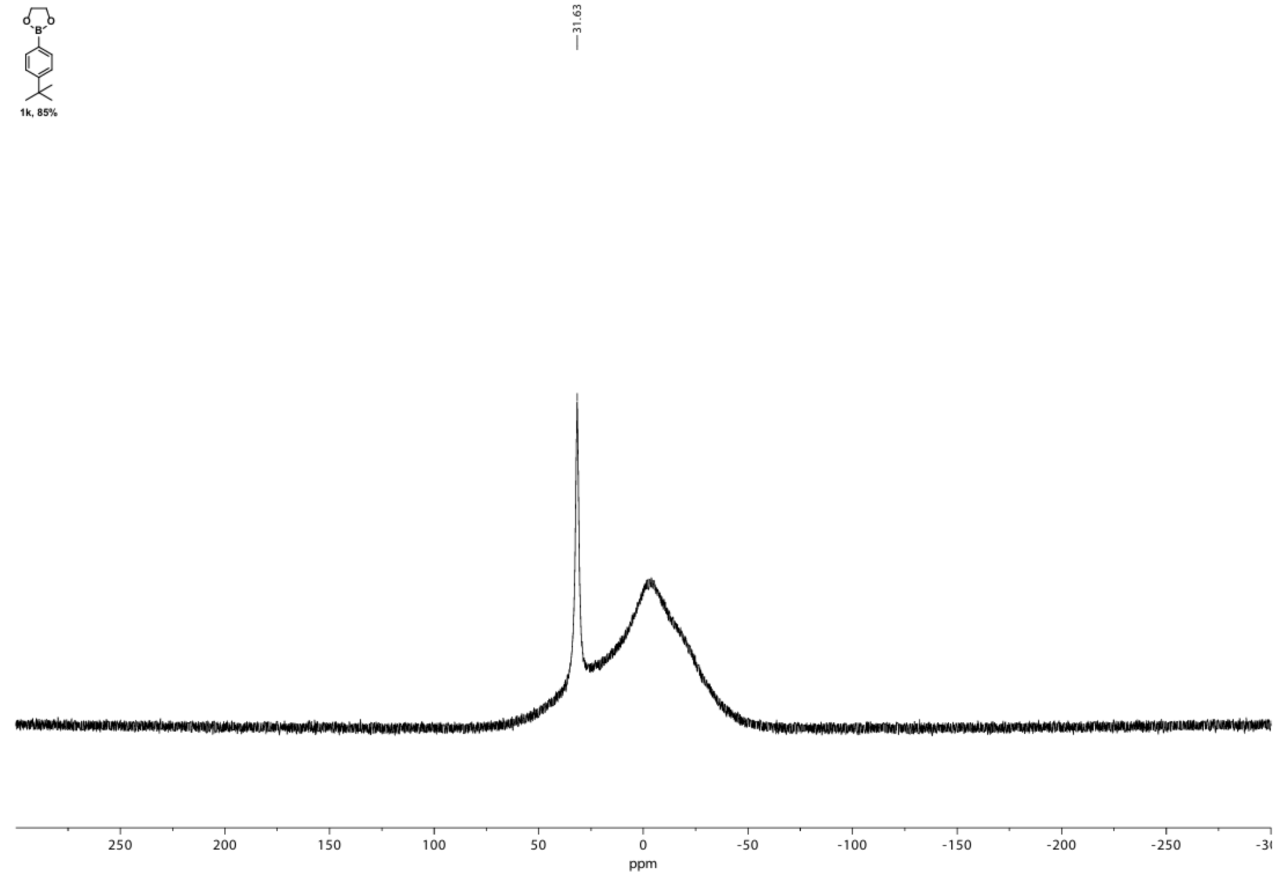

2-(4-tert-butylphenyl)-1,3,2-dioxaborolane

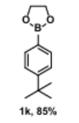

^{13}C NMR ($CDCl_3$, 100.6 MHz)

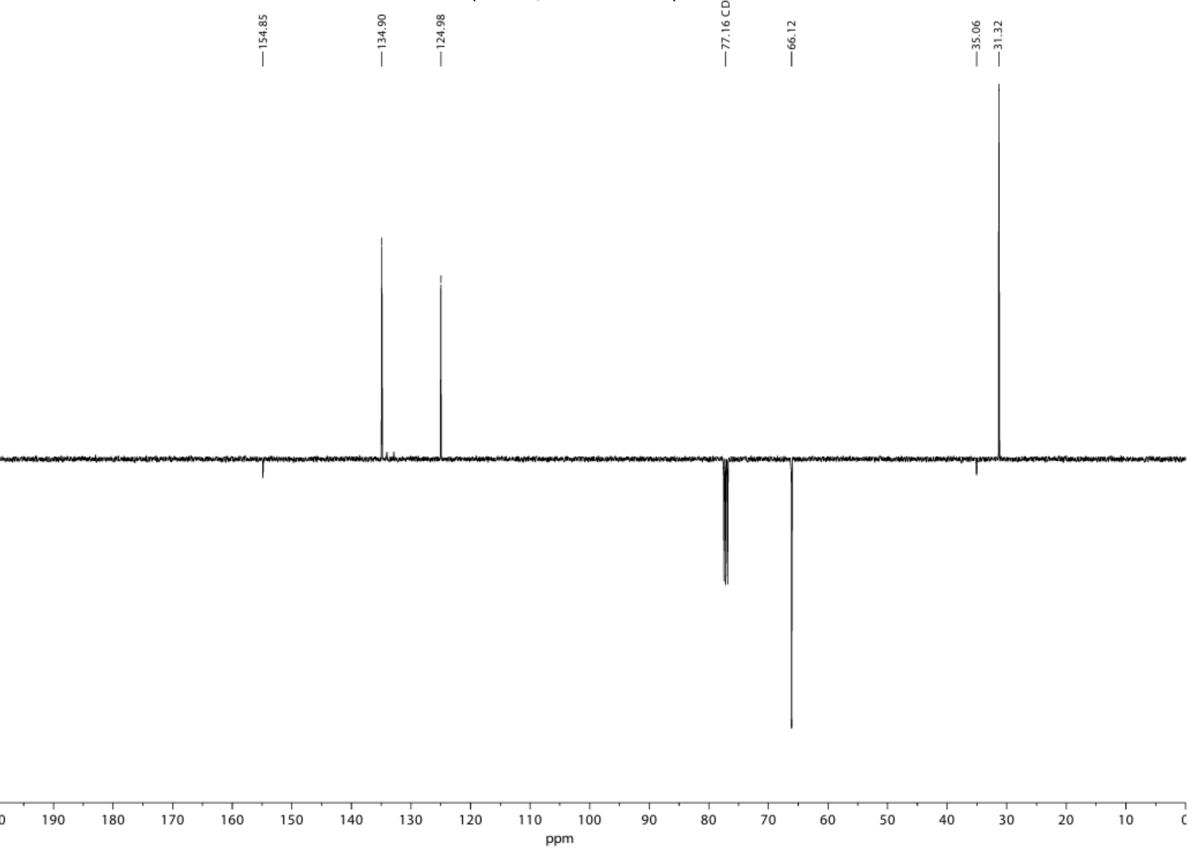

***N*-H-*B,B′,B′′*-tri(4-tert-butylphenyl)borazine**

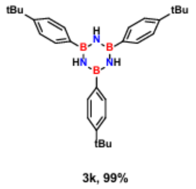

^{1}H NMR ($CDCl_3$, 400 MHz)

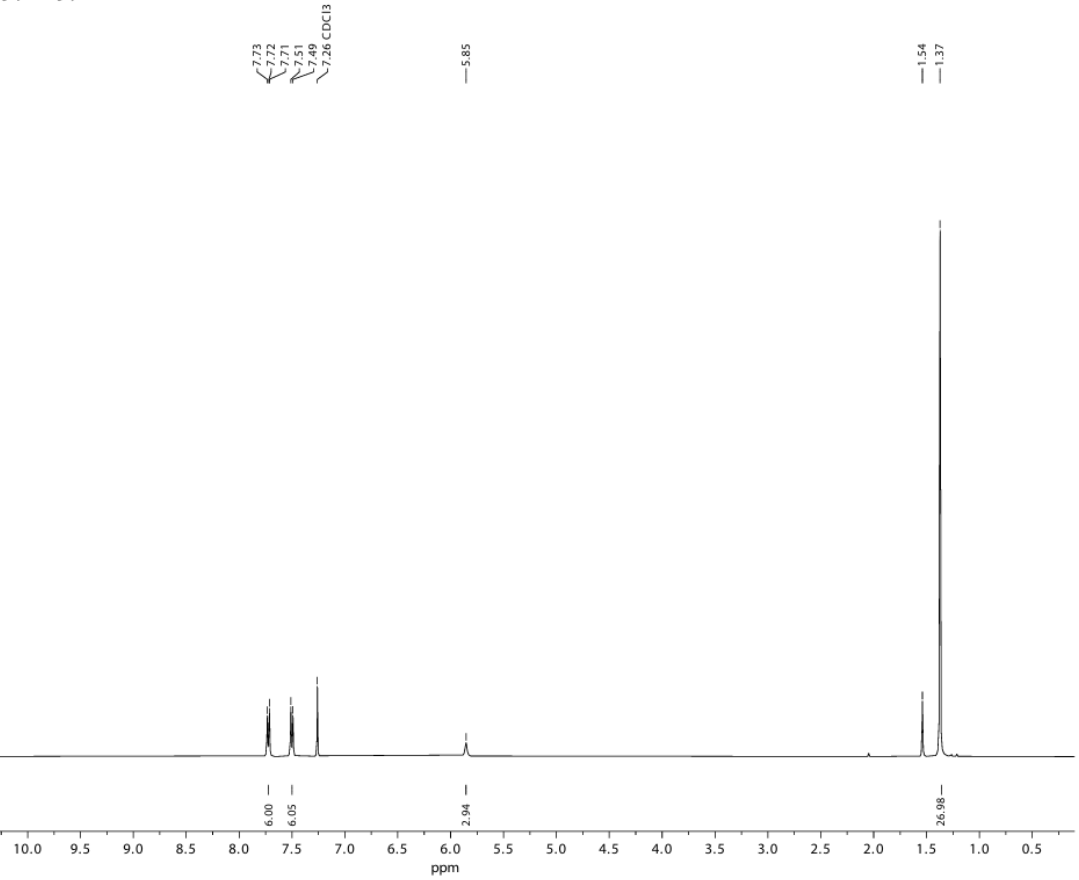

N-H-*B,B′,B″*-tri(4-tert-butylphenyl)borazine

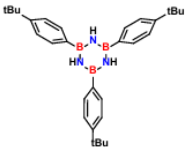

3k, 99%

^{11}B NMR ($CDCl_3$, 128 MHz)

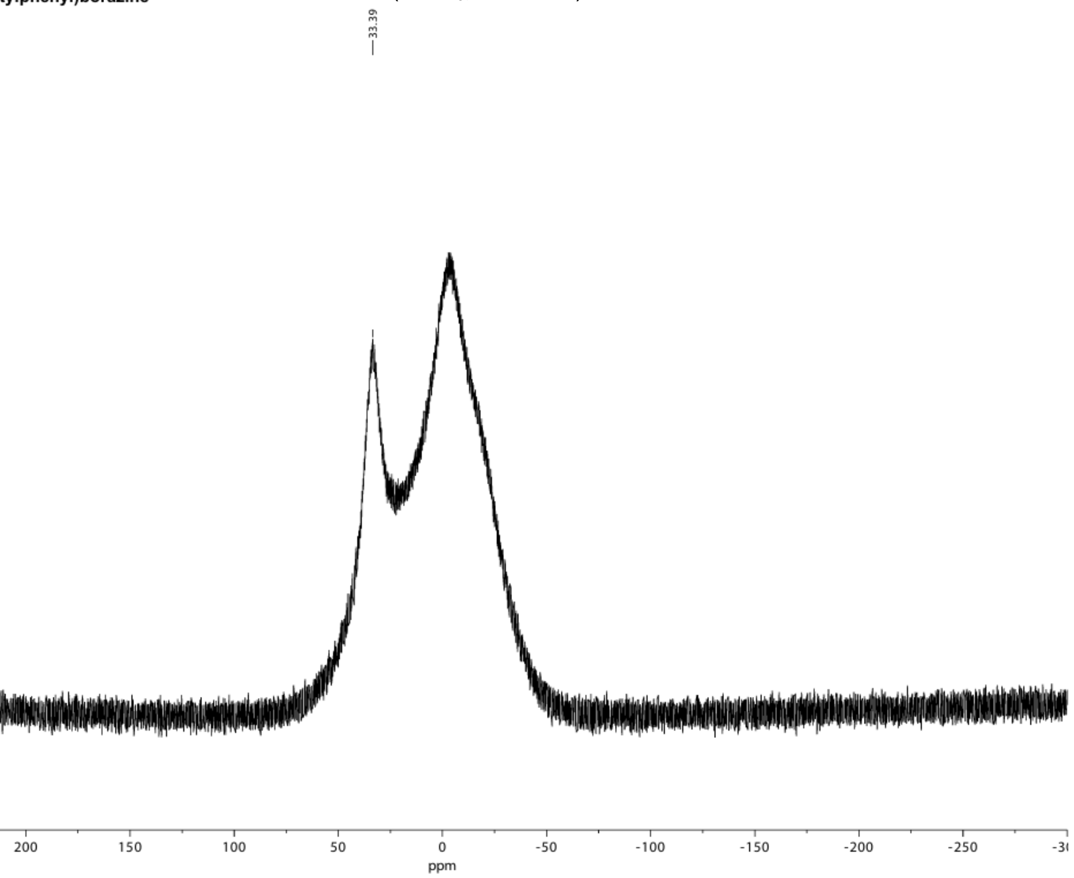

N-H-*B,B′,B″*-tri(4-tert-butylphenyl)borazine

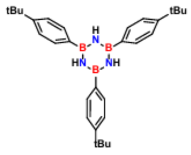

3k, 99%

^{13}C NMR ($CDCl_3$, 100.6 MHz)

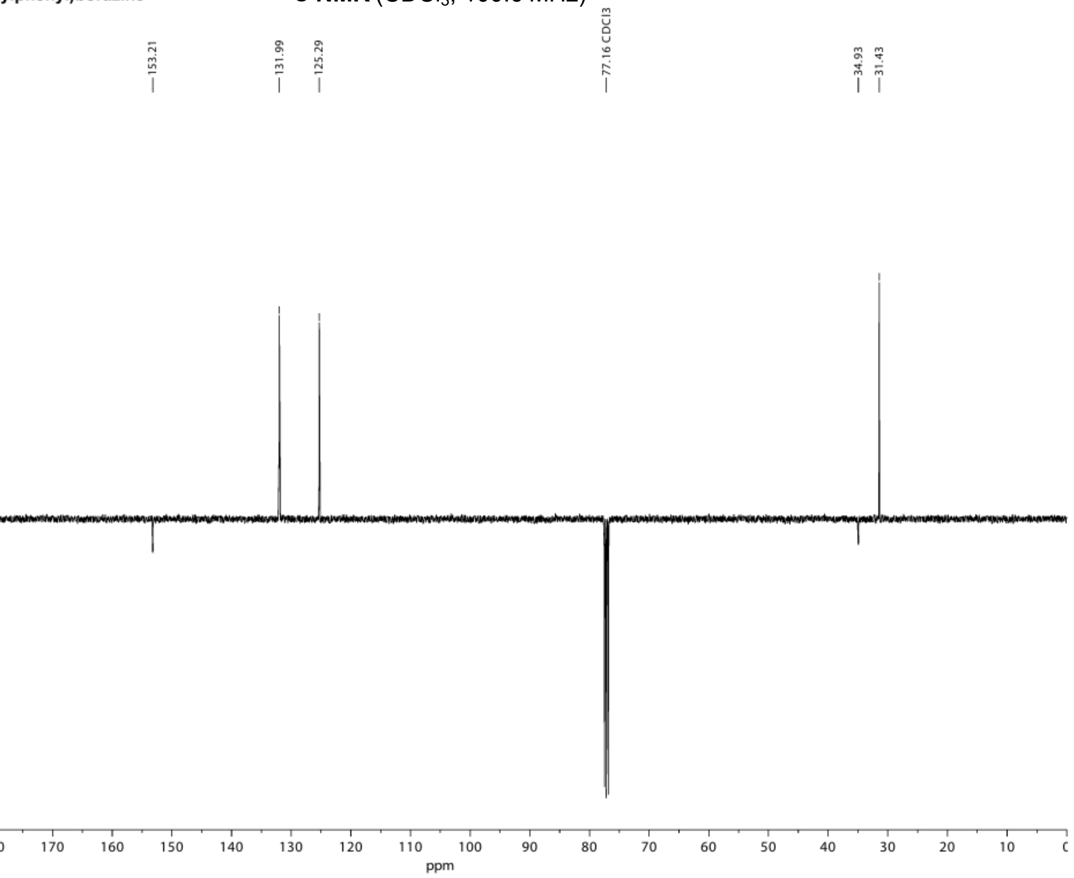

N-H-B,B′,B″-tri(4-methylphenyl)borazine

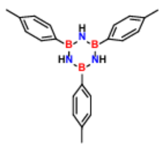

3l, 48%

^{1}H NMR ($CDCl_3$, 400 MHz)

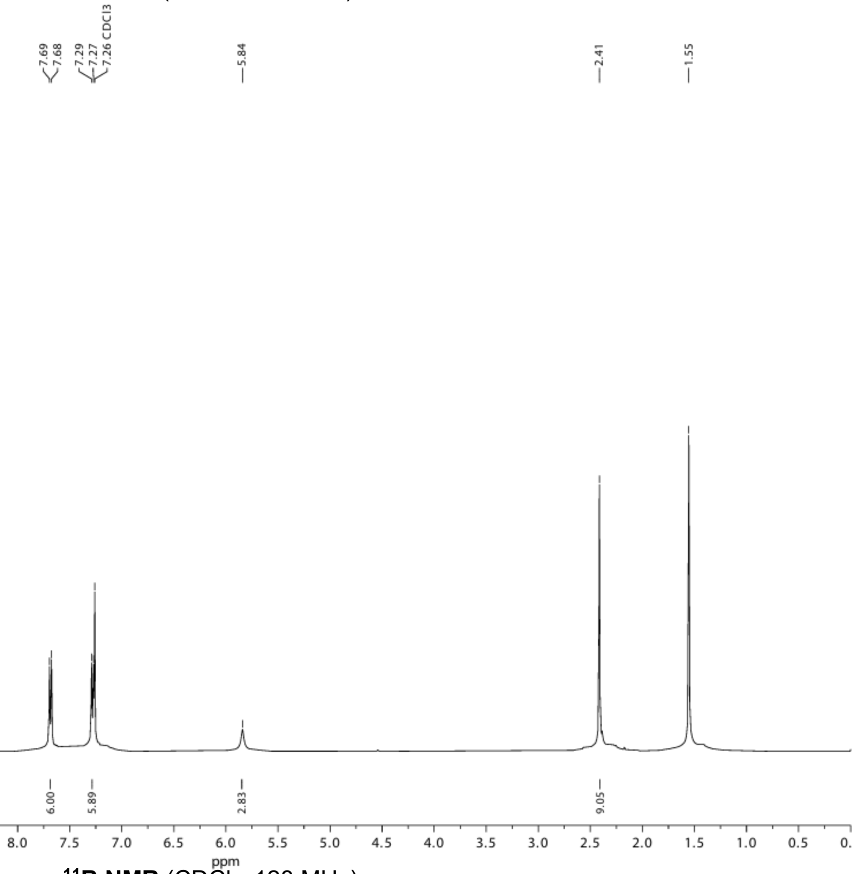

N-H-B,B′,B″-tri(4-methylphenyl)borazine

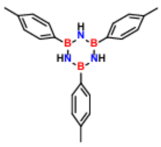

3l, 48%

^{11}B NMR ($CDCl_3$, 128 MHz)

***N*-H-*B*,*B*′,*B*″-tri(4-methylphenyl)borazine**

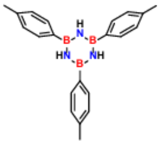

3l, 48%

^{13}C NMR (CDCl$_3$, 100.6 MHz)

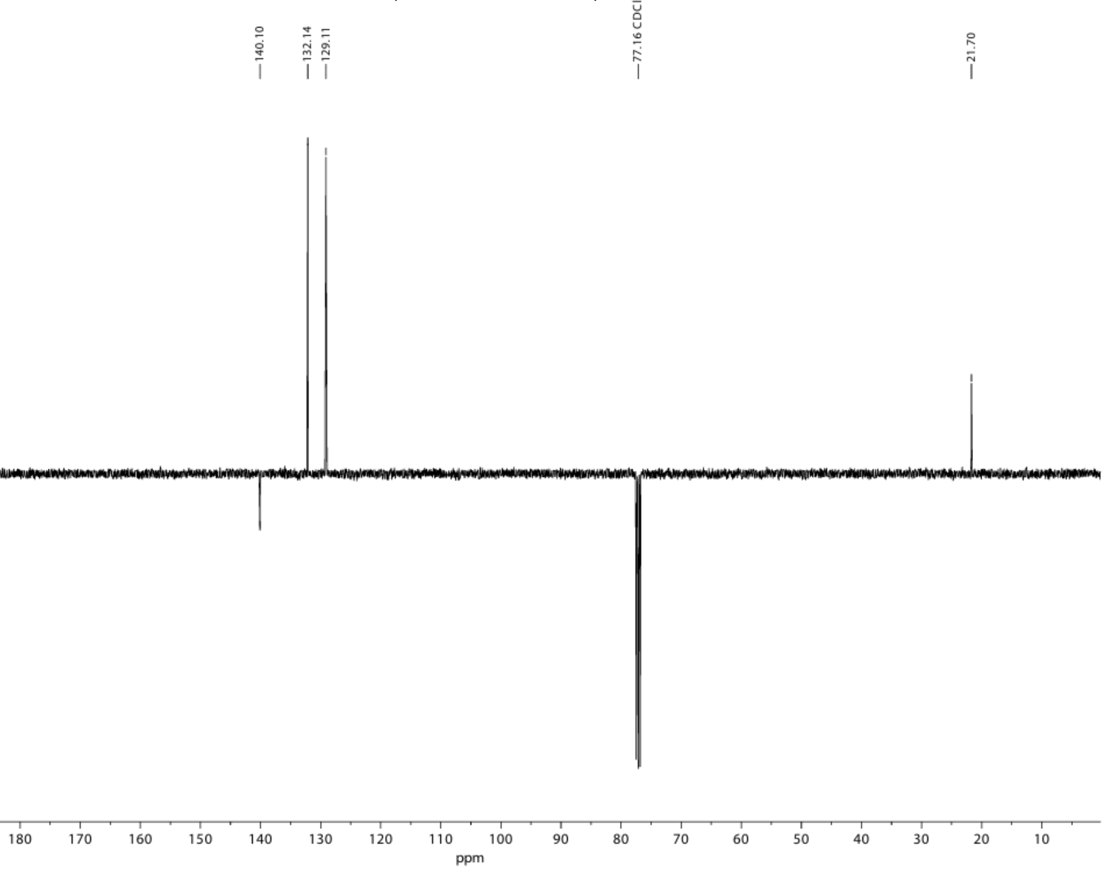

2-(2,4-difluorophenyl)-1,3,2-dioxaborolane

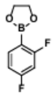

1m, 88%

^{1}H NMR (CDCl$_3$, 400 MHz)

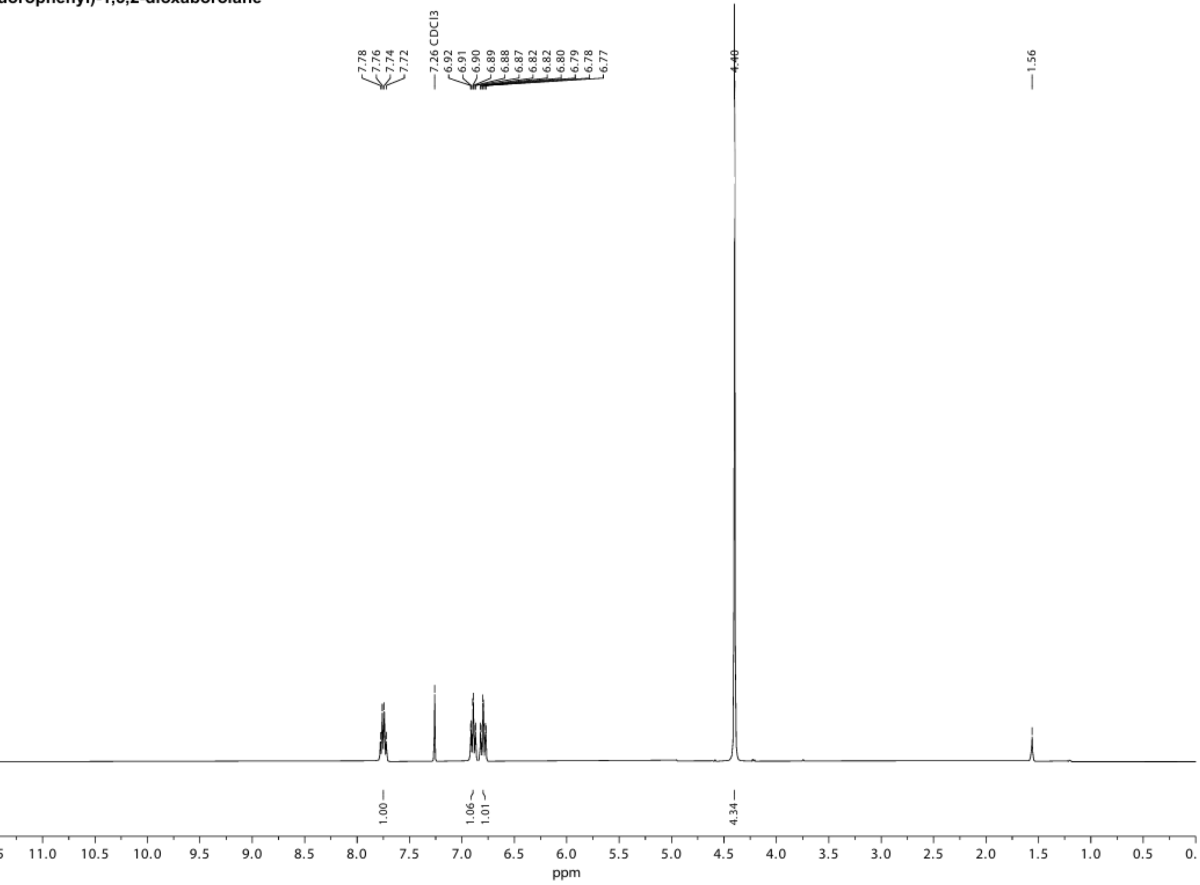

2-(2,4-difluorophenyl)-1,3,2-dioxaborolane ^{11}**B NMR** ($CDCl_3$, 128 MHz)

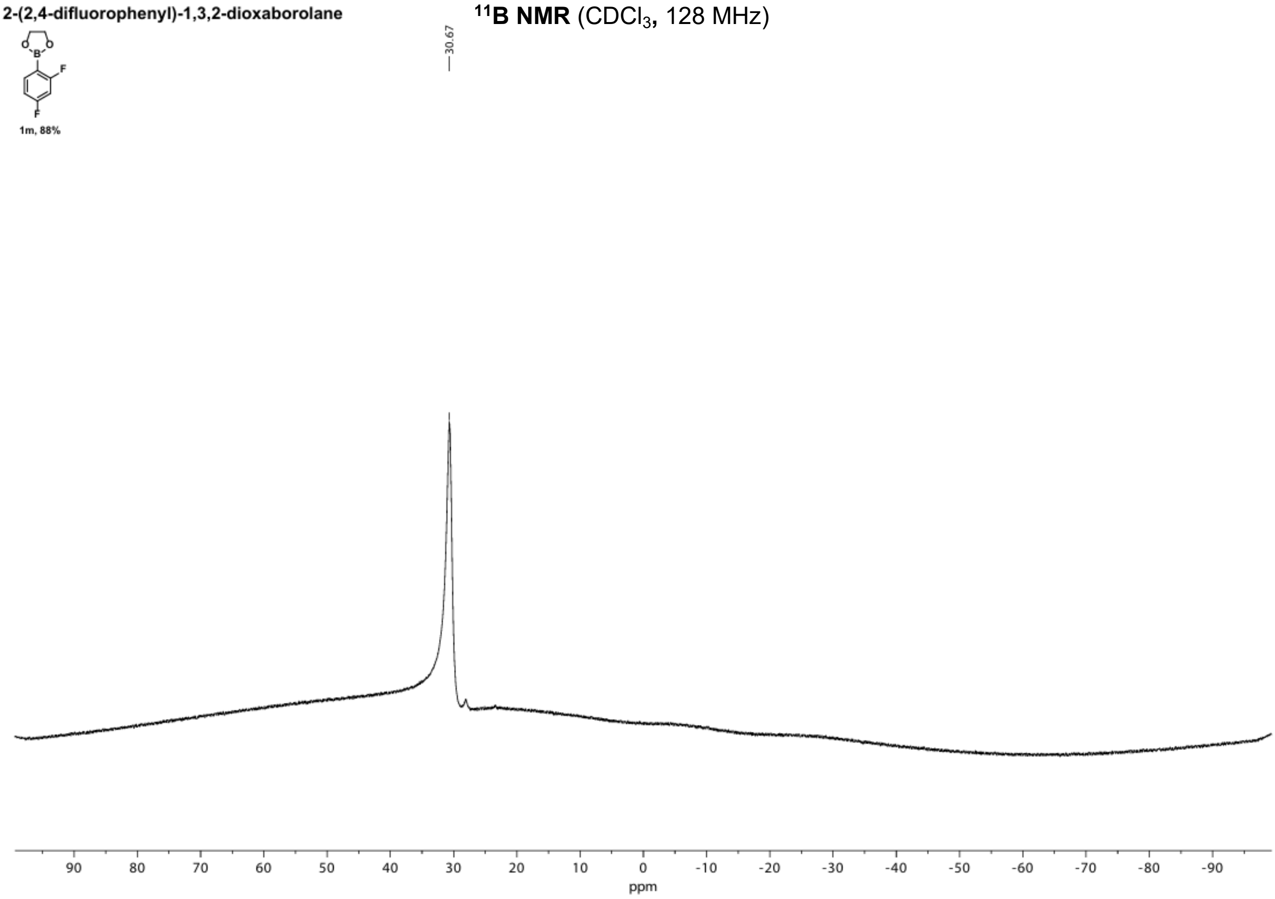

2-(2,4-difluorophenyl)-1,3,2-dioxaborolane ^{13}**C NMR** ($CDCl_3$, 100.6 MHz)

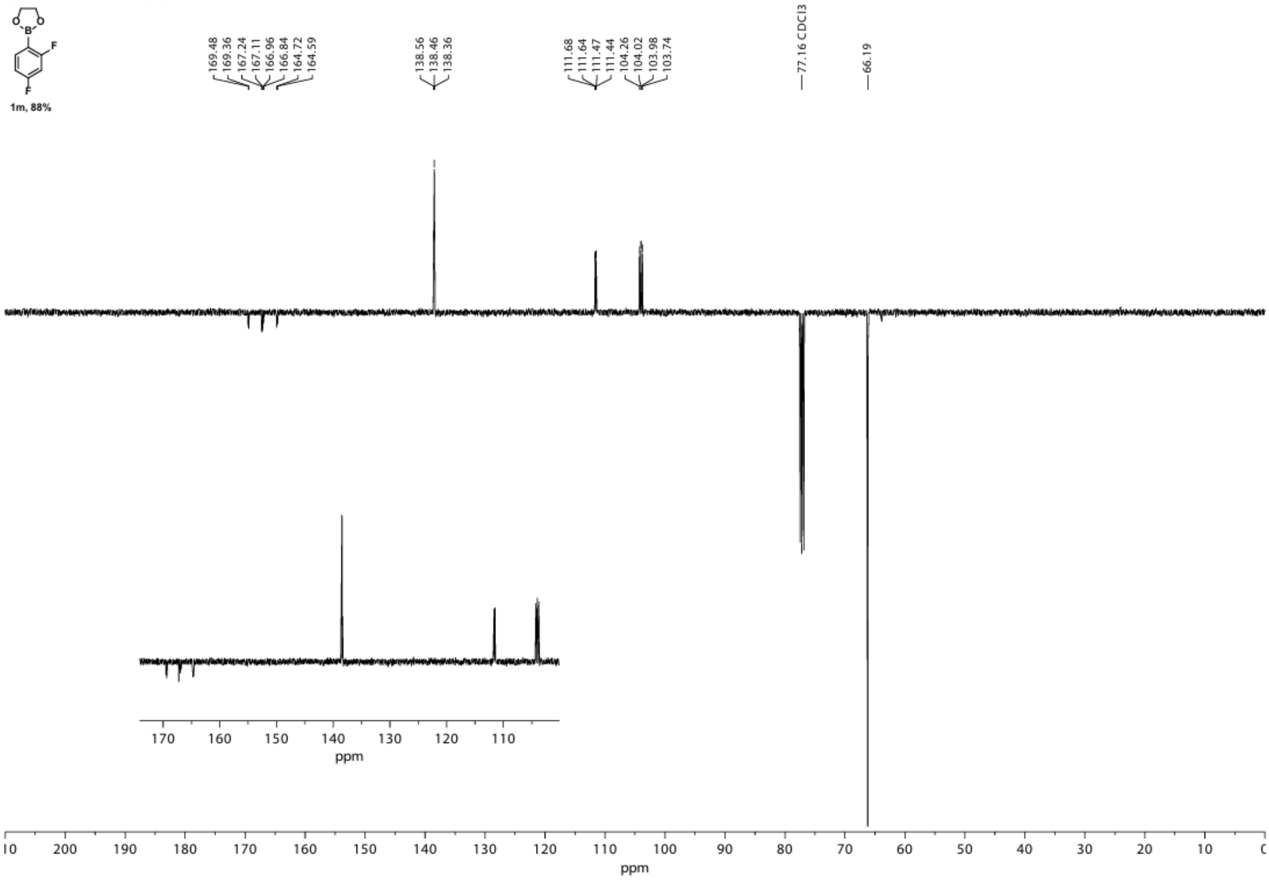

2-(2,4-difluorophenyl)-1,3,2-dioxaborolane

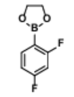

1m, 88%

^{19}F NMR ($CDCl_3$, 376.4 MHz)

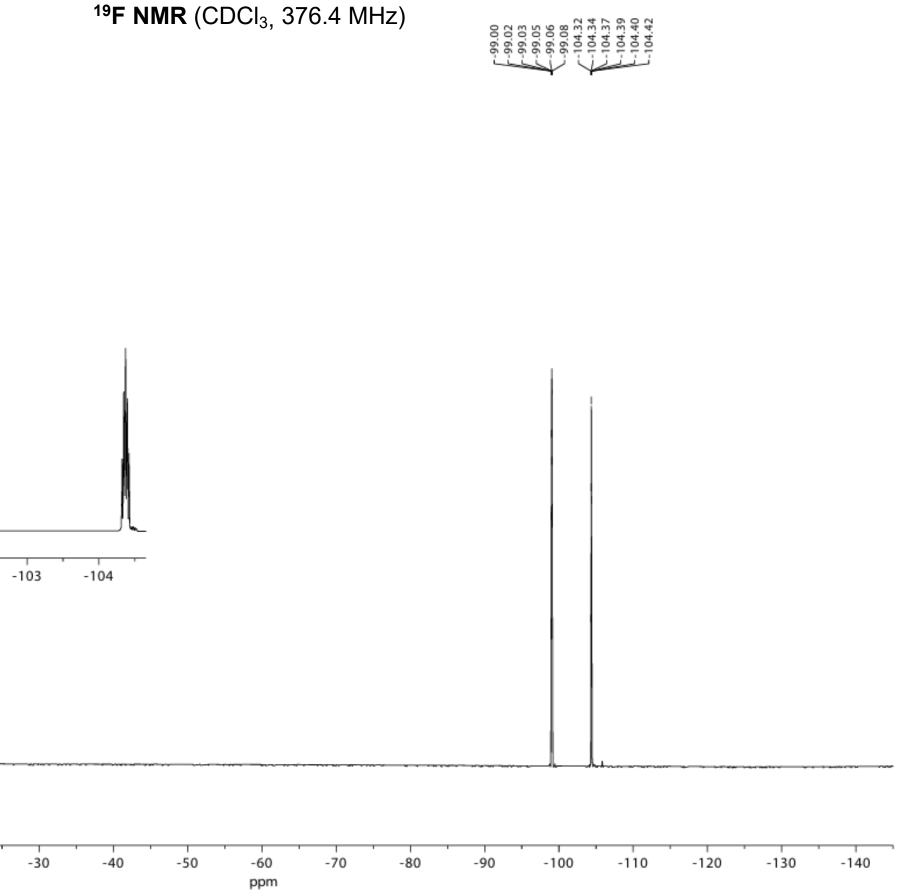

***N*-H-*B,B′,B′′*-tri(2,4- difluorophenyl)borazine**

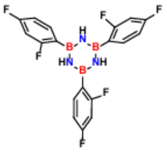

3m, 62%

^{1}H NMR ($CDCl_3$, 400 MHz)

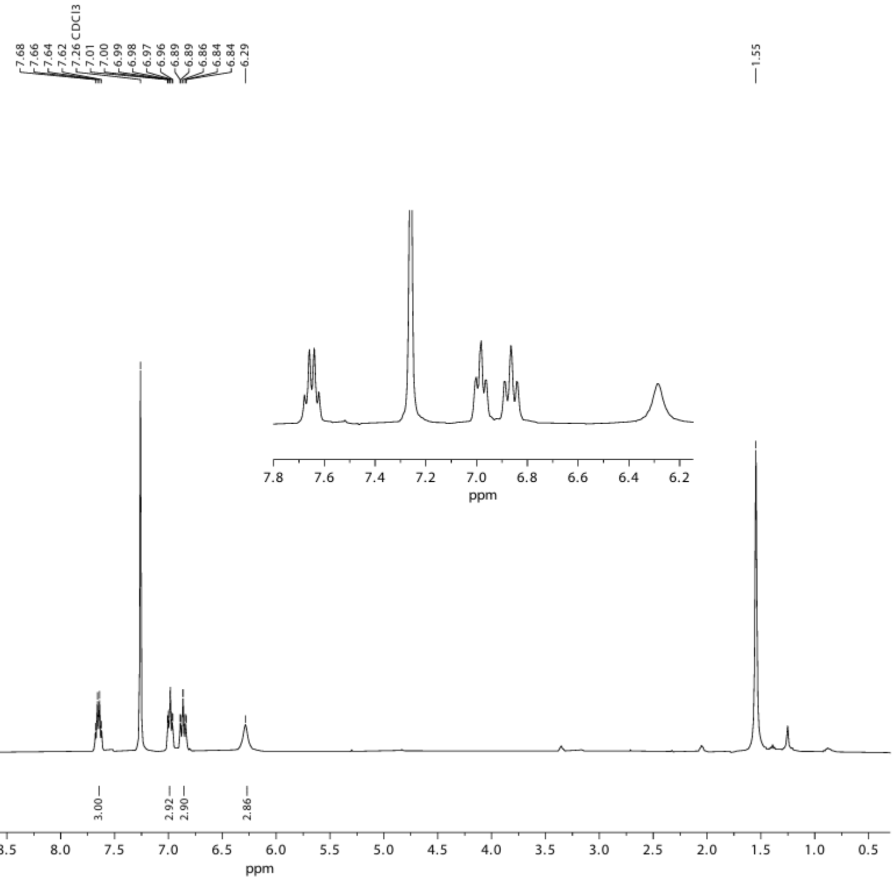

^{11}B NMR (CDCl$_3$, 128 MHz)

***N*-H-*B*,*B′*,*B′′*-tri(2,4- difluorophenyl)borazine**

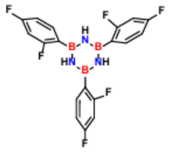

3m, 62%

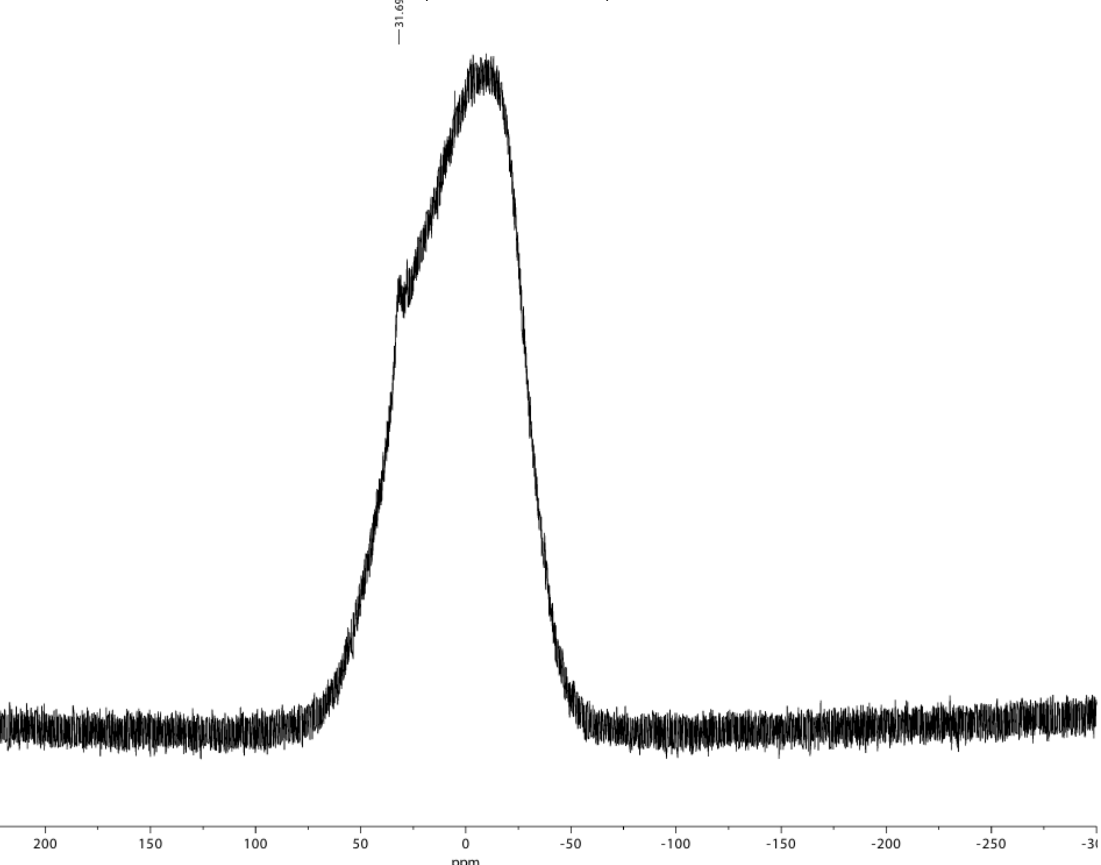

^{19}F NMR (CDCl$_3$, 376.4 MHz)

***N*-H-*B*,*B′*,*B′′*-tri(2,4- difluorophenyl)borazine**

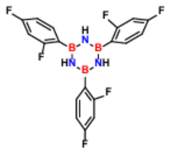

3m, 62%

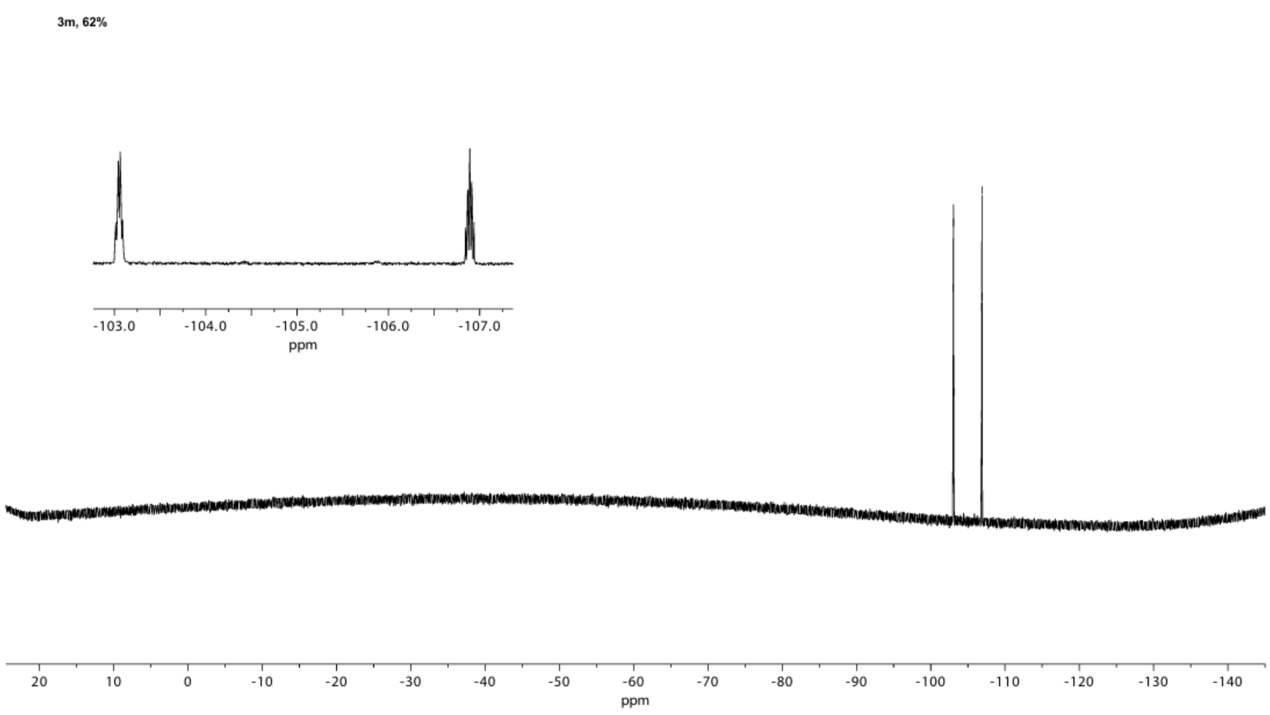

^{1}H NMR ($CDCl_3$, 400 MHz)

***N*-H-*B,B′,B″*-tri(3-fluorophenyl)borazine**

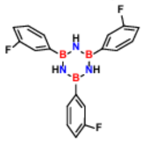

3n, 75%

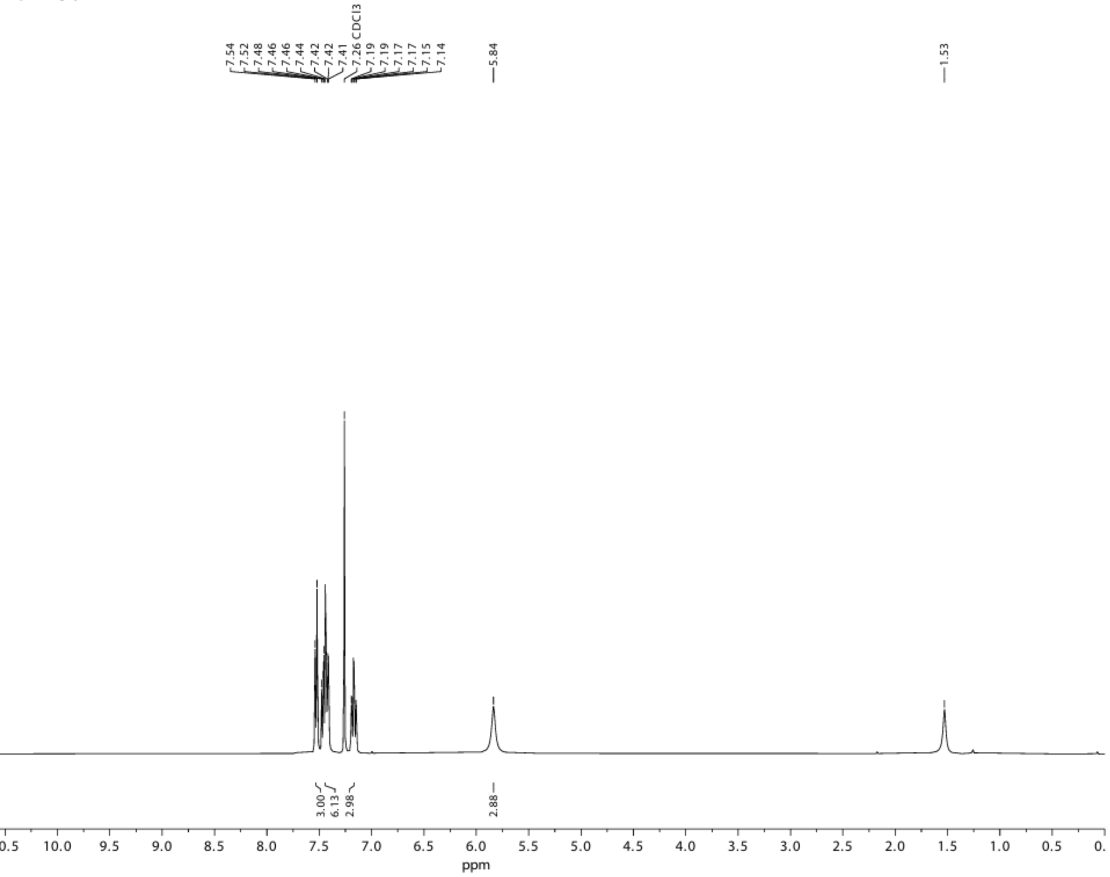

^{11}B NMR ($CDCl_3$, 128 MHz)

***N*-H-*B,B′,B″*-tri(3-fluorophenyl)borazine**

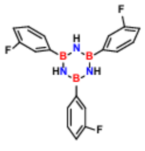

3n, 75%

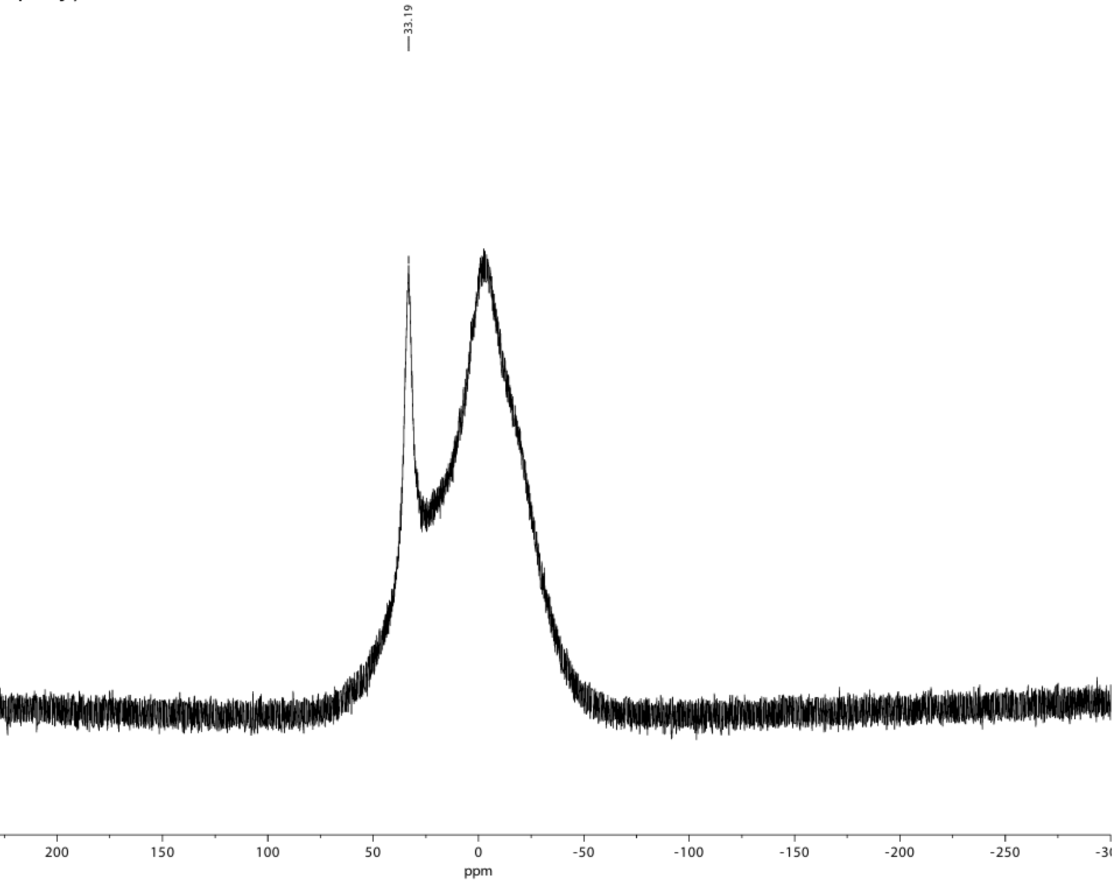

^{13}C NMR ($CDCl_3$, 100.6 MHz)

***N*-H-*B,B′,B′′*-tri(3-fluorophenyl)borazine**

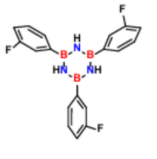

3n, 75%

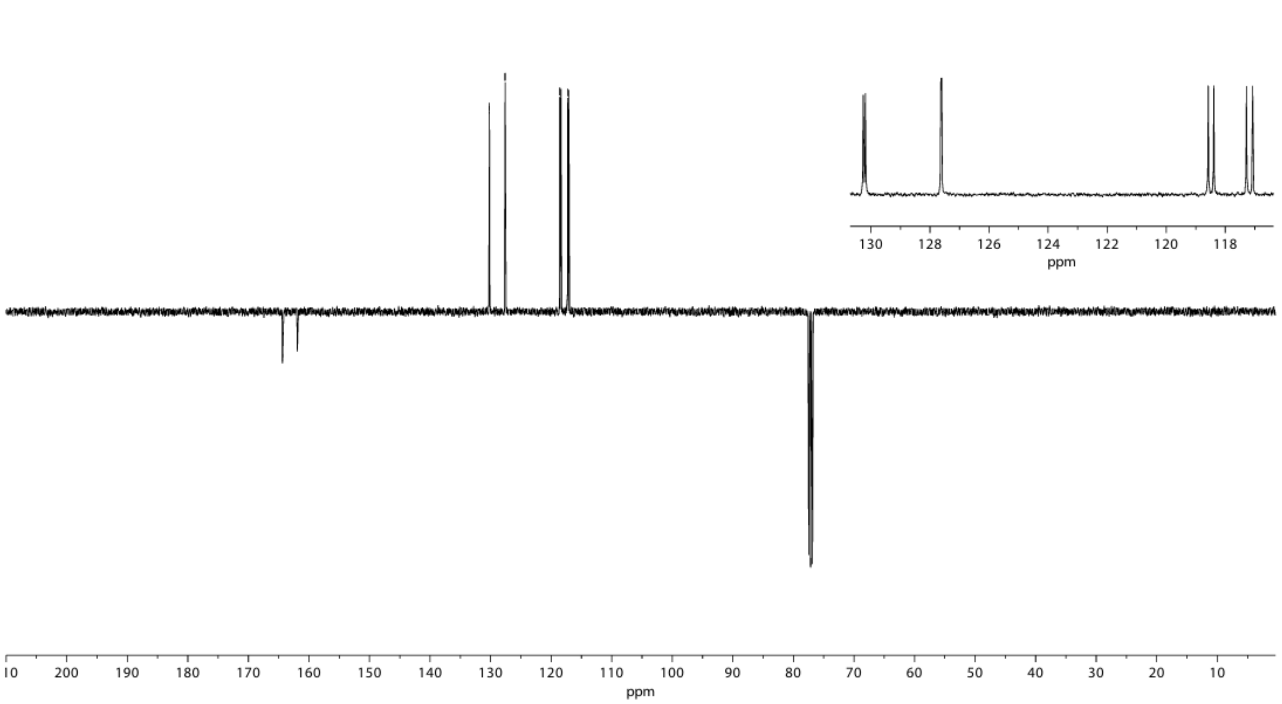

^{19}F NMR ($CDCl_3$, 376.4 MHz)

\-H-*B,B′,B′′*-tri(3-fluorophenyl)borazine

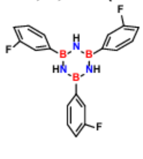

3n, 75%

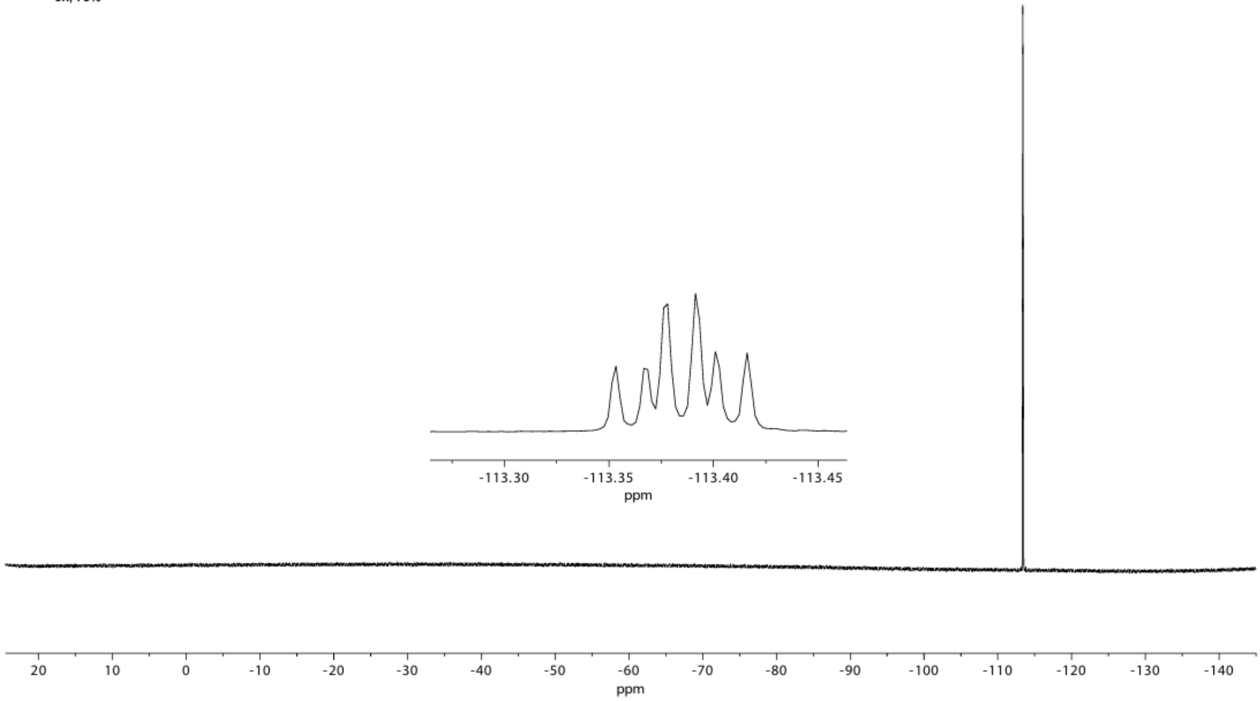

***N*-H-*B,B′,B′′*-tri(m-tolyl)borazine**

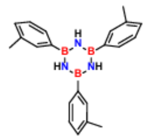

3o, 55%

^{1}H NMR ($CDCl_3$, 400 MHz)

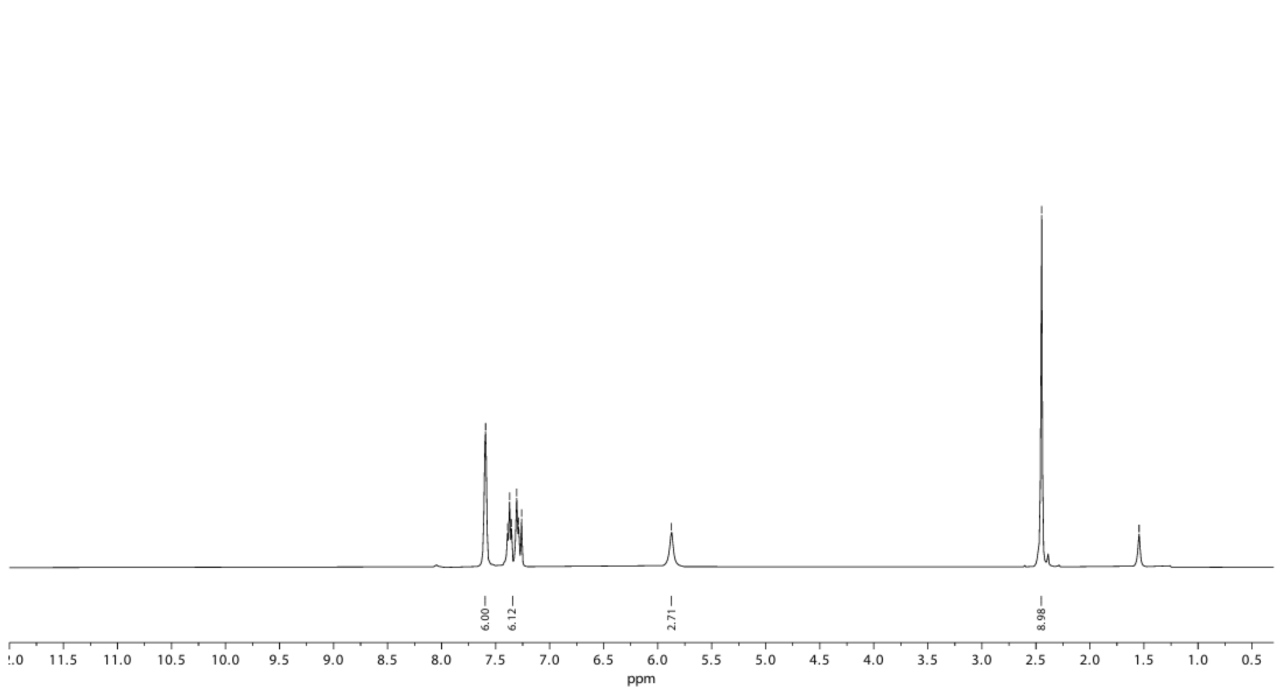

***N*-H-*B,B′,B′′*-tri(m-tolyl)borazine**

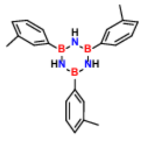

3o, 55%

^{11}B NMR ($CDCl_3$, 128 MHz)

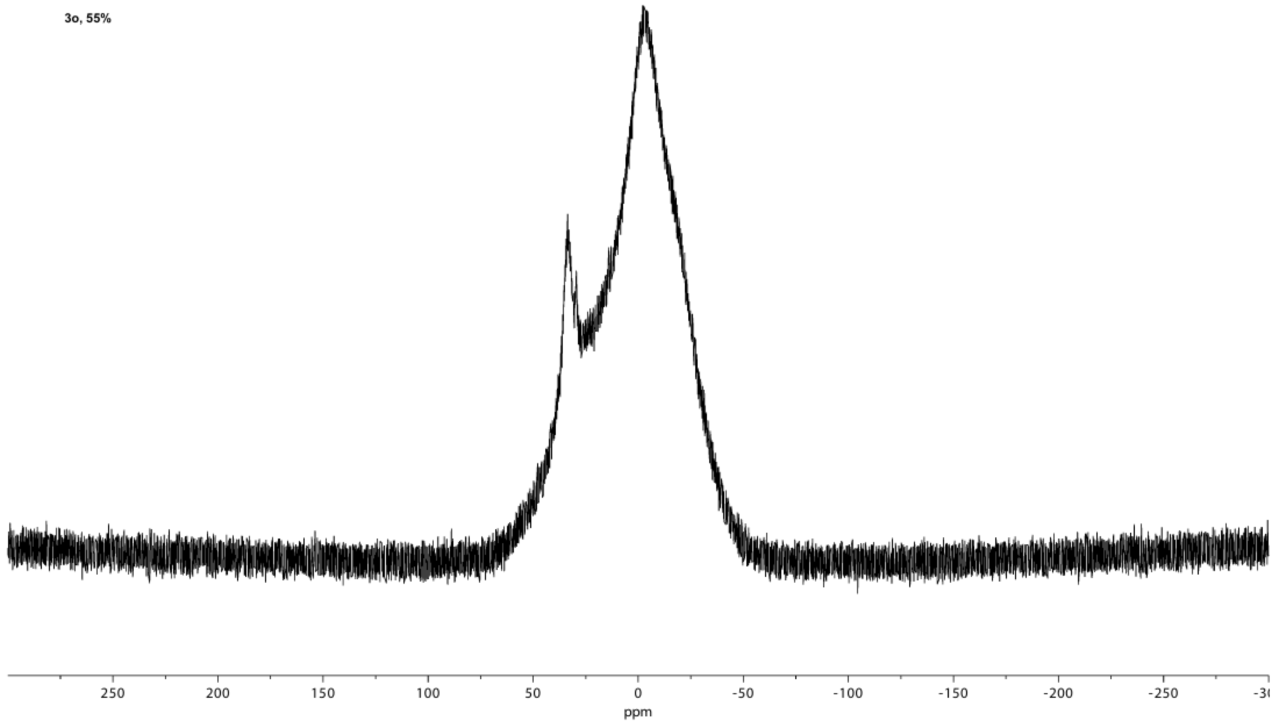

***N*-H-*B*,*B′*,*B″*-tri(m-tolyl)borazine**

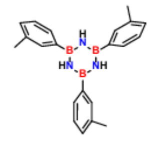

3o, 55%

^{13}C NMR ($CDCl_3$, 100.6 MHz)

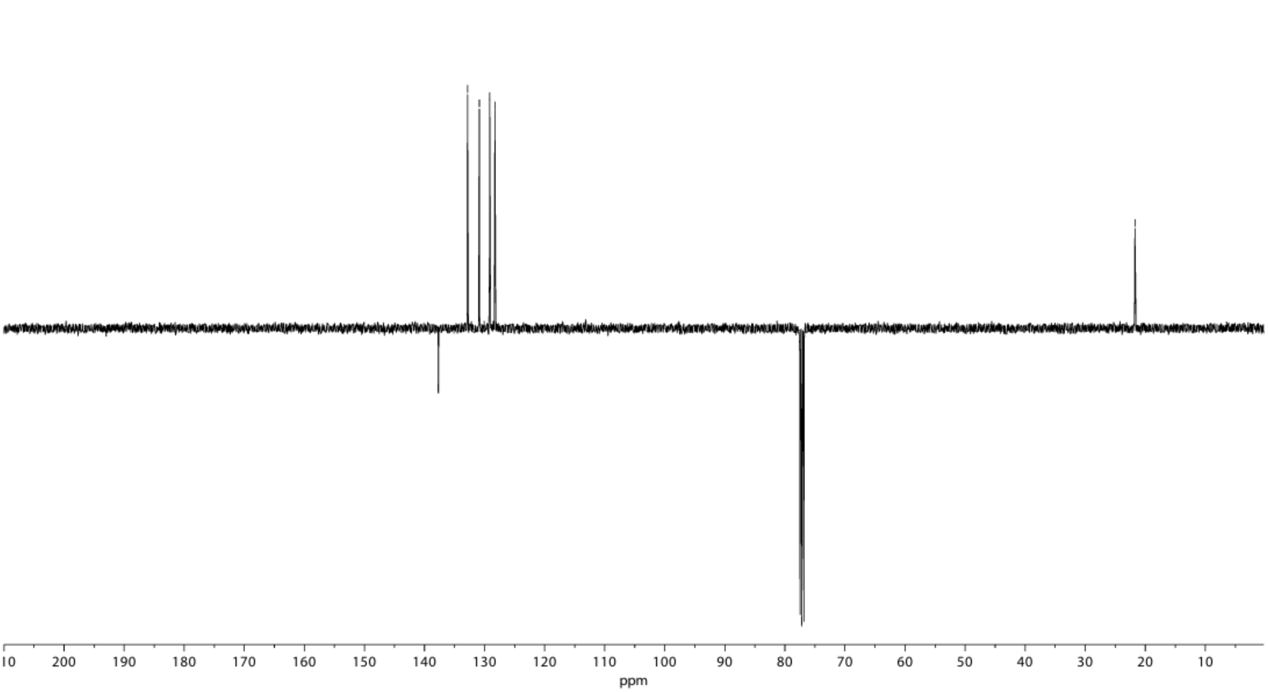

^{1}H NMR ($CDCl_3$, 400 MHz)

***N*-H-*B*,*B′*,*B″*-tri(2-bromophenyl)borazine**

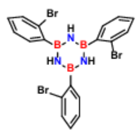

3p, 46%

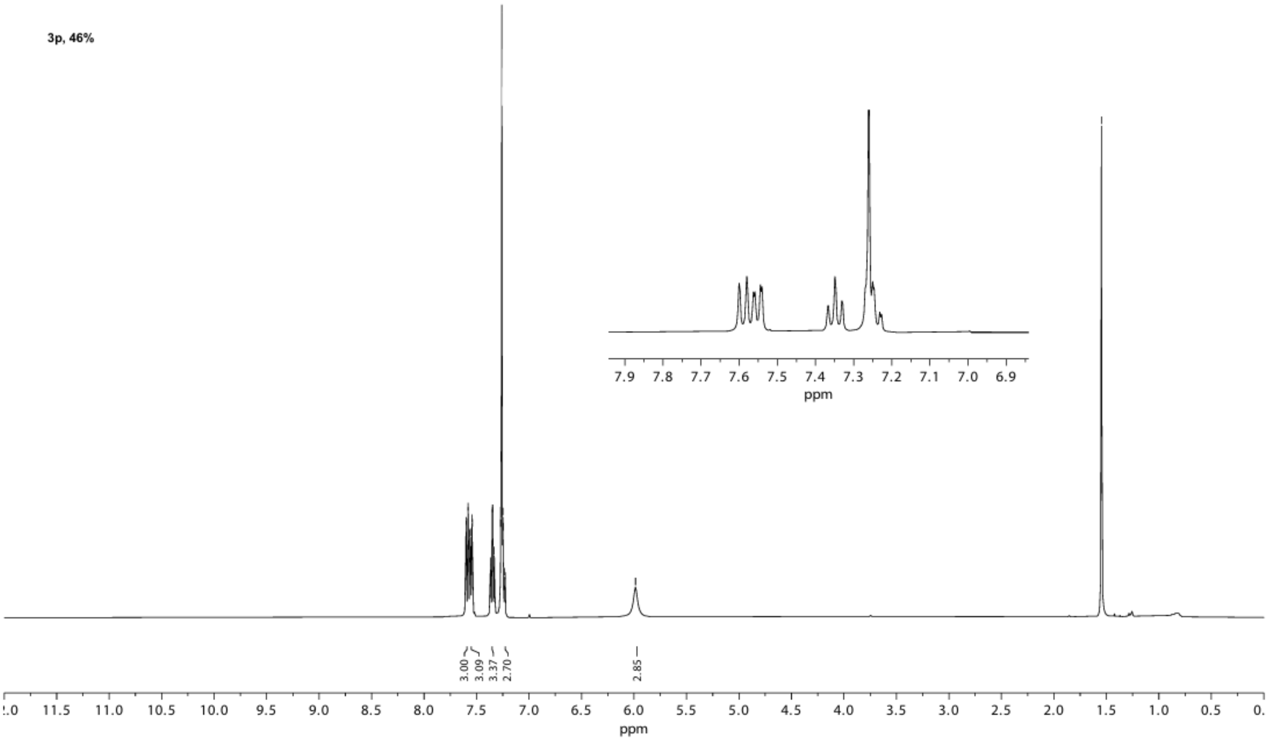

^{11}B NMR (CDCl$_3$, 128 MHz)

***N*-H-*B,B′,B″*-tri(2-bromophenyl)borazine**

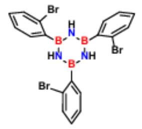

3p, 46%

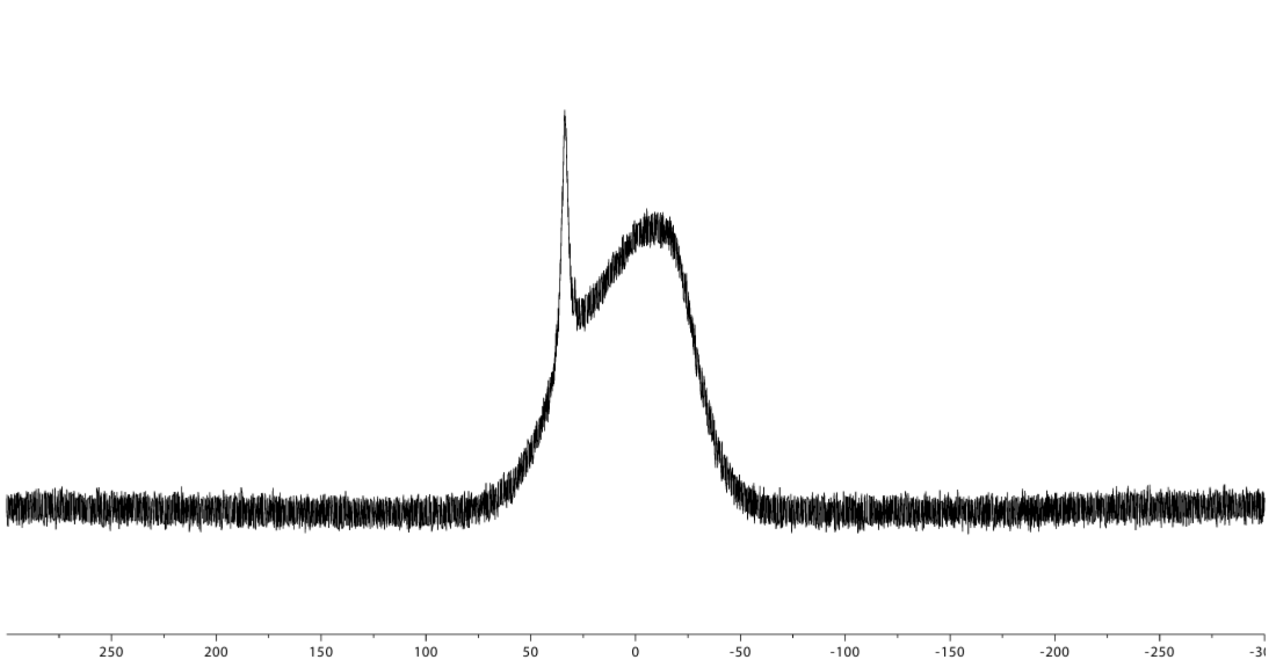

^{13}C NMR (CDCl$_3$, 100.6 MHz)

***N*-H-*B,B′,B″*-tri(2-bromophenyl)borazine**

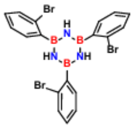

3p, 46%

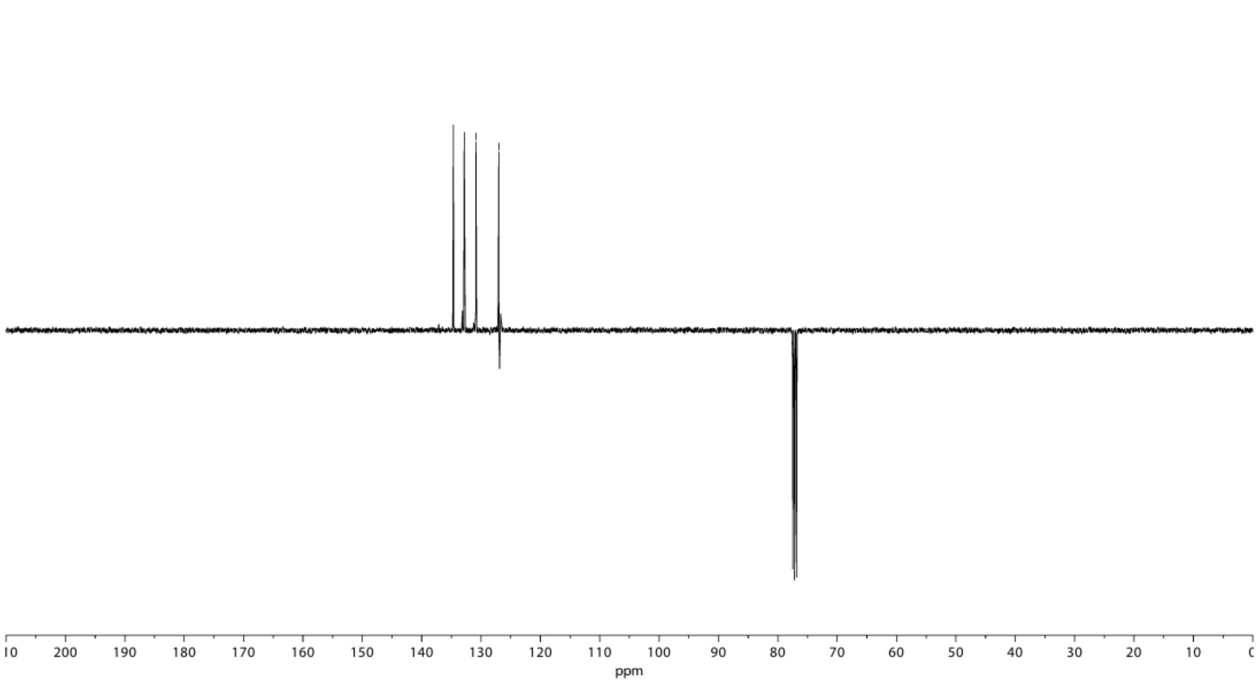

2-(2,6-difluorophenyl)-1,3,2-dioxaborolane

^{1}H NMR ($CDCl_3$, 400 MHz)

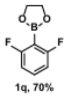

1q, 70%

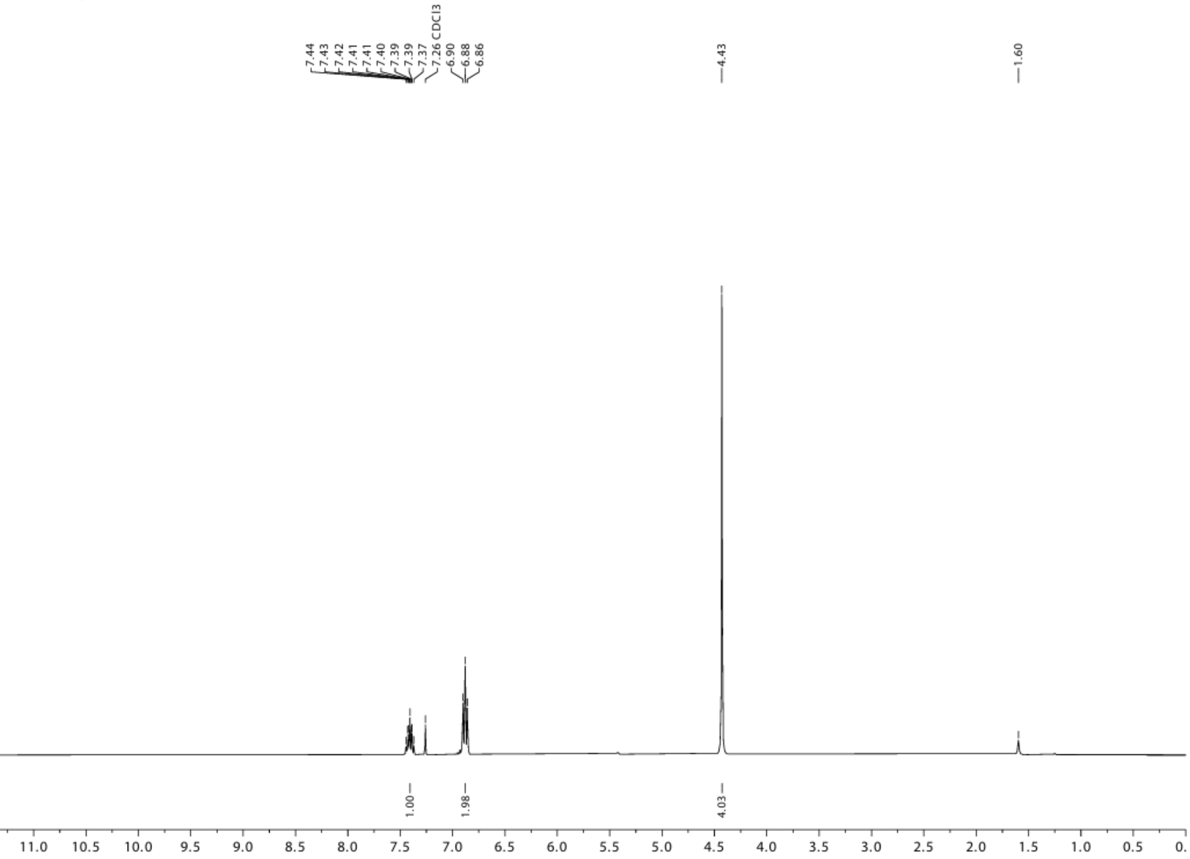

^{11}B NMR ($CDCl_3$**,** 128 MHz)

2-(2,6-difluorophenyl)-1,3,2-dioxaborolane

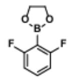

1q, 70%

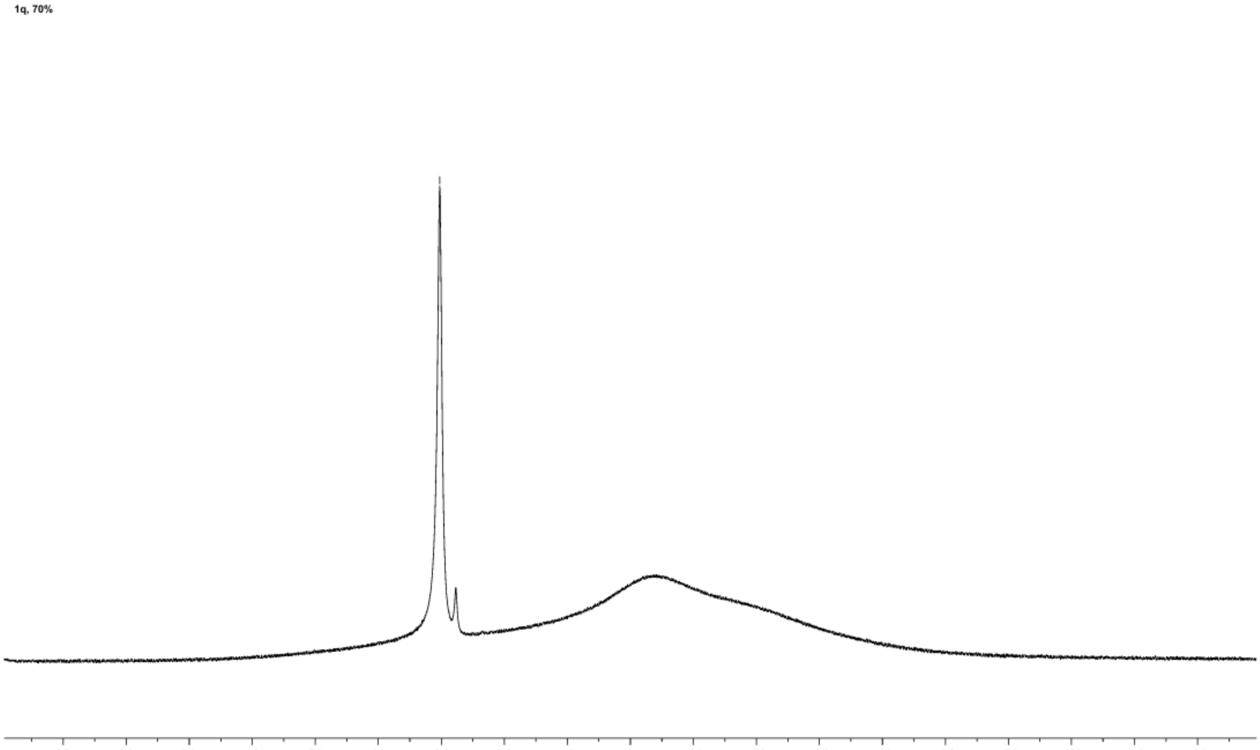

2-(2,6-difluorophenyl)-1,3,2-dioxaborolane

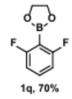

1q, 70%

^{13}C NMR (CDCl$_3$, 100.6 MHz)

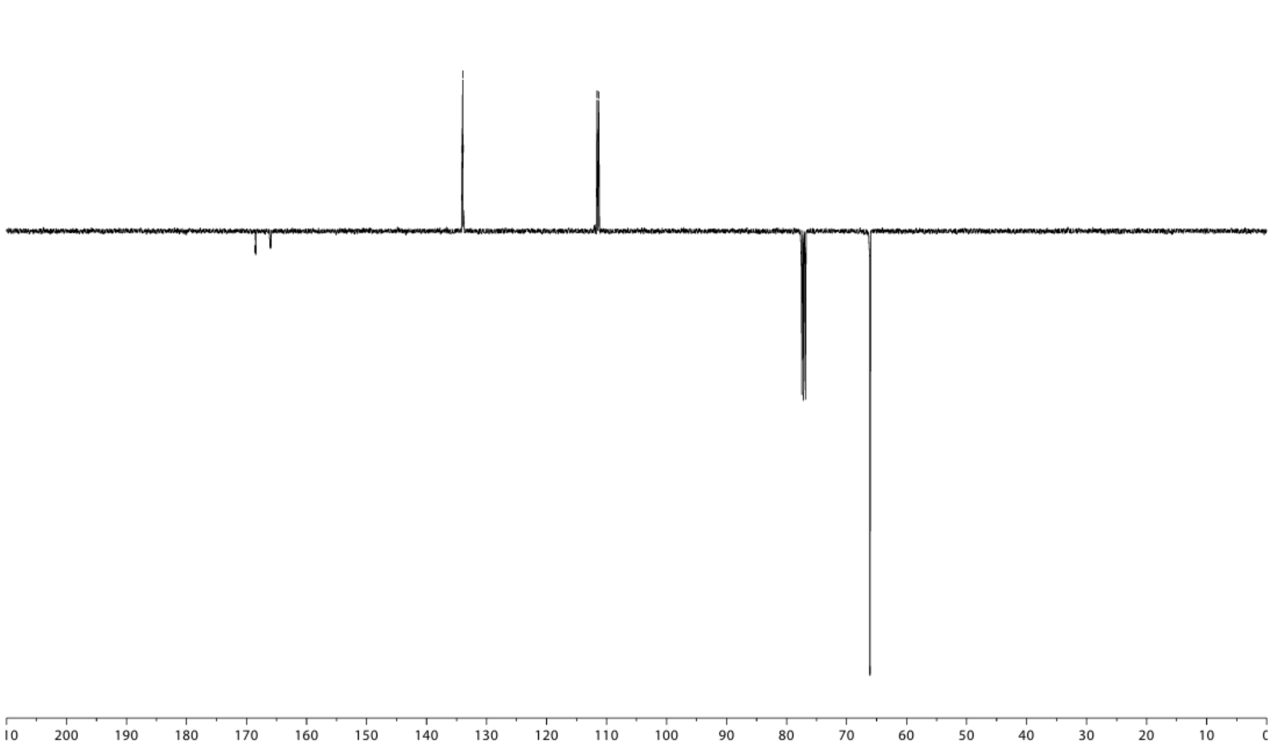

2-(2,6-difluorophenyl)-1,3,2-dioxaborolane

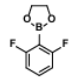

1q, 70%

^{19}F NMR (CDCl$_3$, 376.4 MHz)

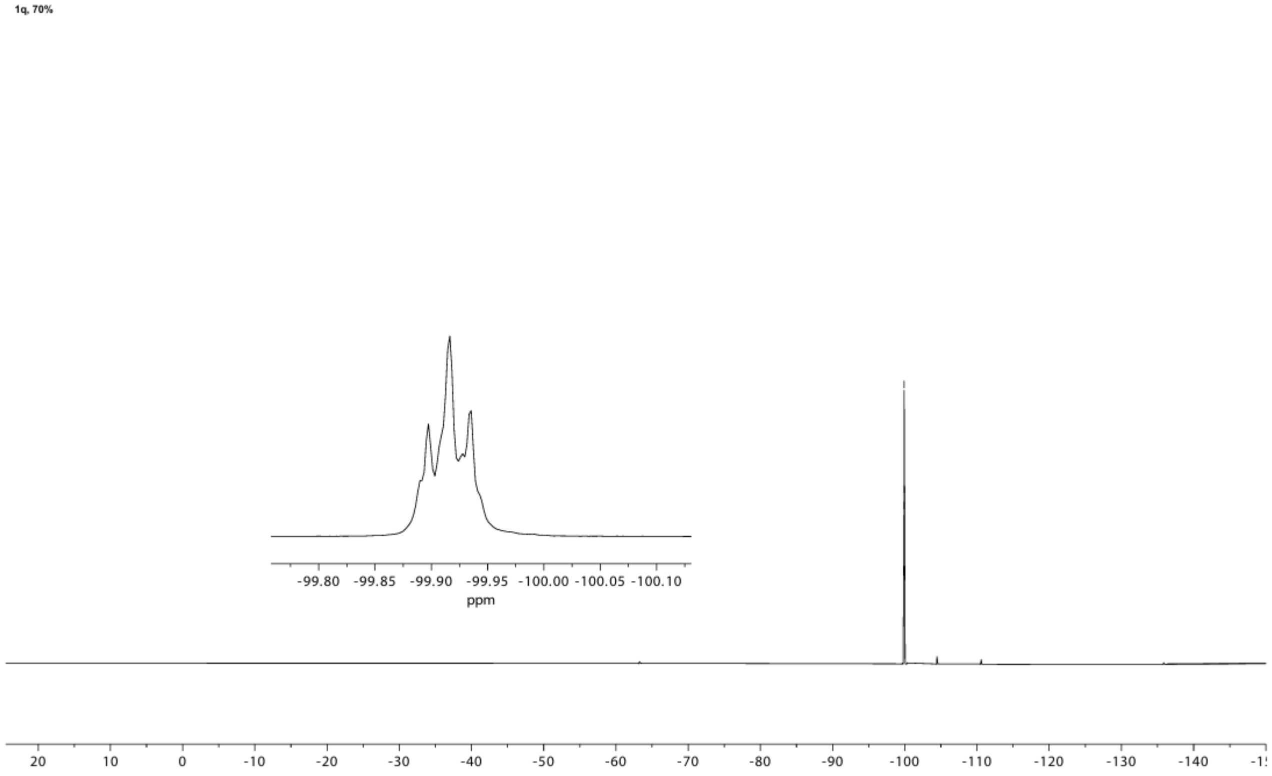

***B,B′,B″*-tri(2,6-difluorophenyl)borazine**

^{1}H NMR ($CDCl_3$, 400 MHz)

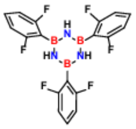

3q, 82%

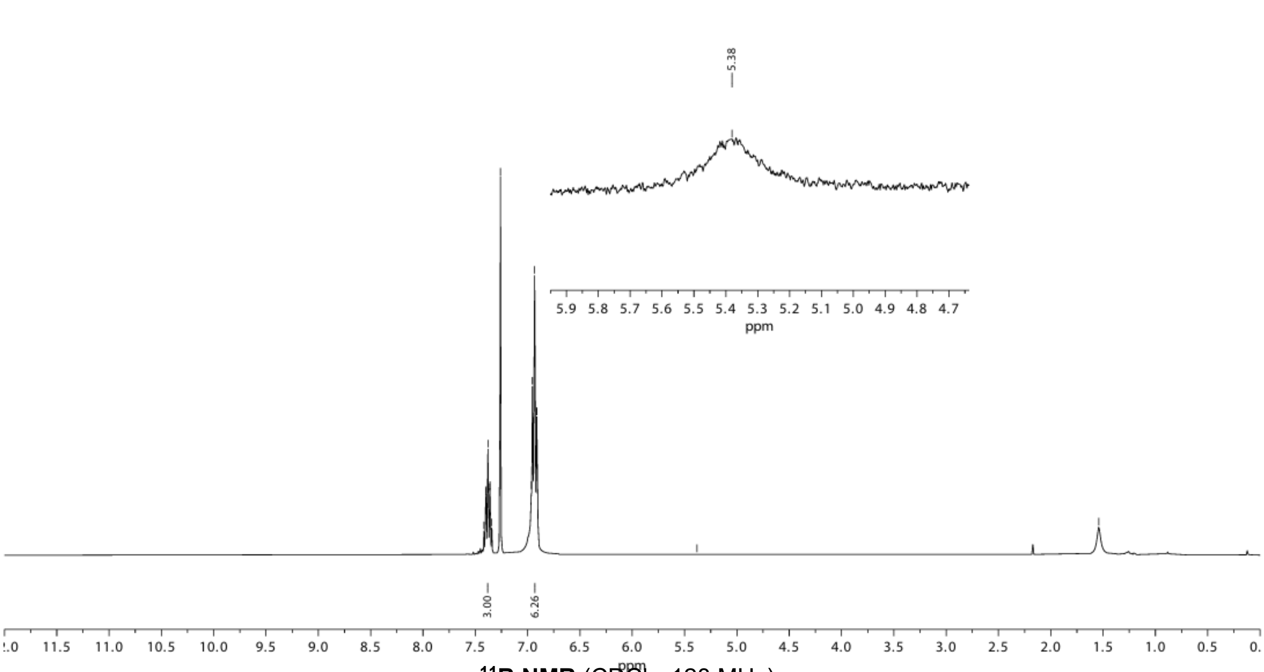

^{11}B NMR ($CDCl_3$**,** 128 MHz)

***B,B′,B″*-tri(2,6-difluorophenyl)borazine**

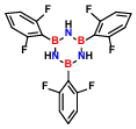

3q, 82%

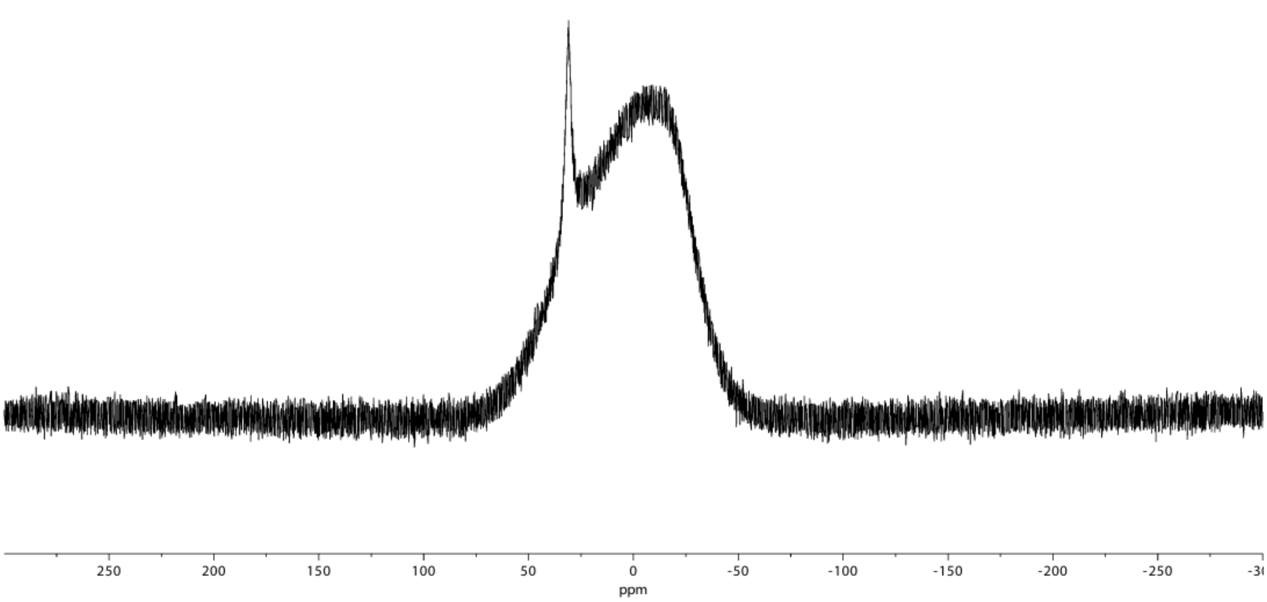

***B,B′,B″*-tri(2,6-difluorophenyl)borazine**

^{13}C NMR ($CDCl_3$, 100.6 MHz)

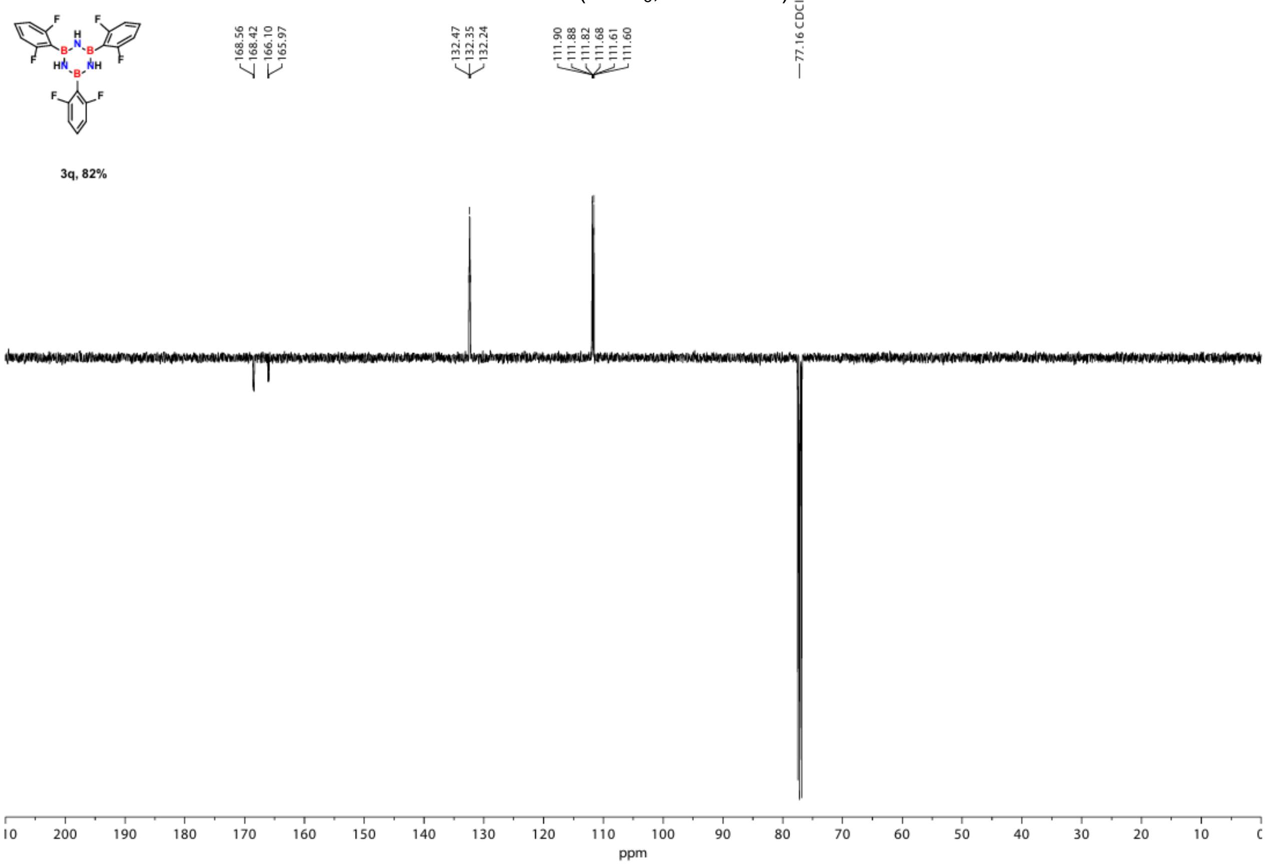

3q, 82%

^{19}F NMR ($CDCl_3$, 376.4 MHz)

***B,B′,B″*-tri(2,6-difluorophenyl)borazine**

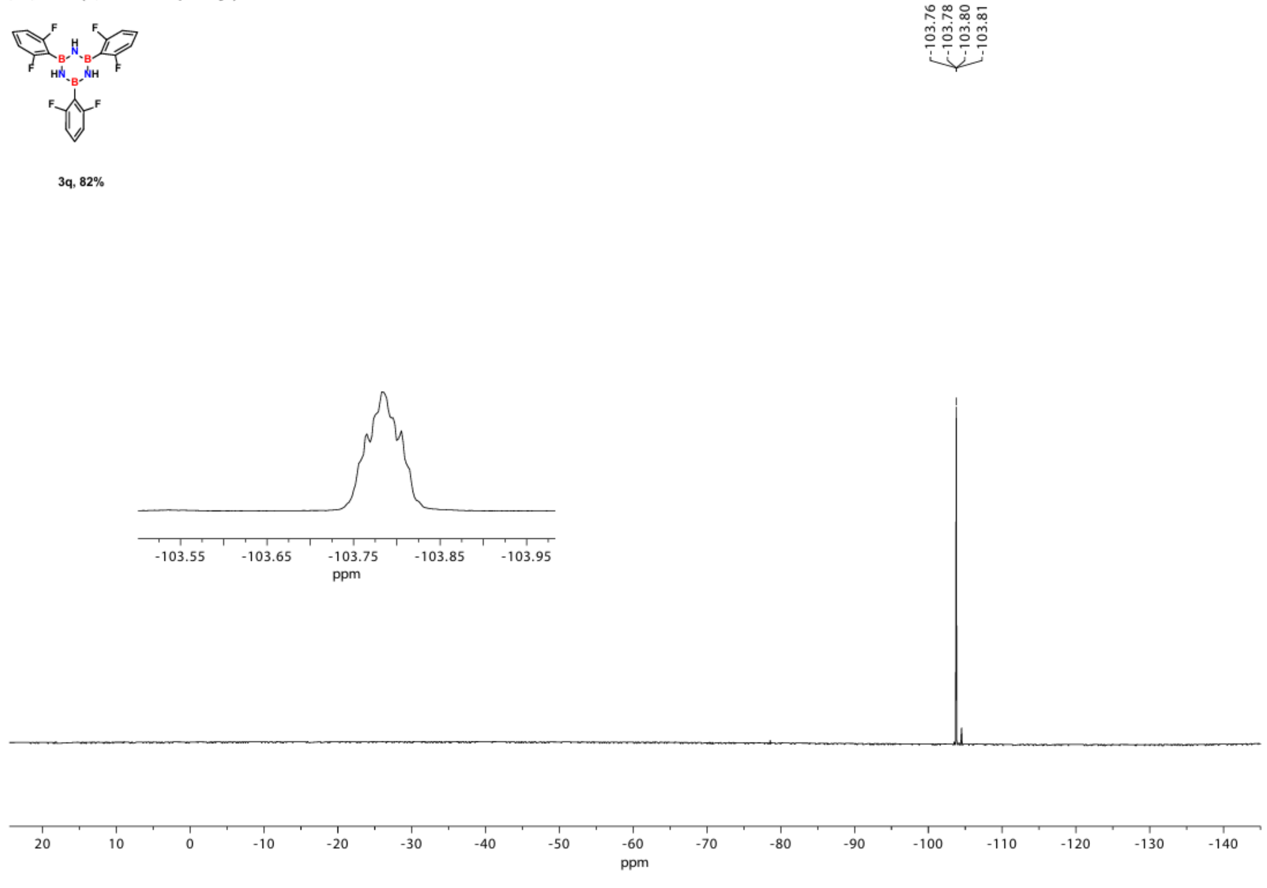

3q, 82%
